# TM7SF2-induced lipid reprogramming promotes cell proliferation and migration via CPT1A/Wnt/β-Catenin axis in cervical cancer cells

**DOI:** 10.1038/s41420-024-01975-8

**Published:** 2024-05-01

**Authors:** Hejing Liu, Yi Liu, Yujia Zhou, Xin Chen, Shuya Pan, Qingfeng Zhou, Huihui Ji, Xueqiong Zhu

**Affiliations:** Zhejiang Provincial Clinical Research Center for Obstetrics and Gynecology, Department of Obstetrics and Gynecology, The Second Affiliated Hospital of Wenzhou Medical University, Wenzhou, 325027 China

**Keywords:** Cervical cancer, Cancer metabolism

## Abstract

Cervical cancer poses a serious threat to women’s health globally. Our previous studies found that upregulation of TM7SF2, which works as an enzyme involved in the process of cholesterol biosynthesis expression, was highly correlated with cervical cancer. However, the mechanistic basis of TM7SF2 promoting cervical cancer progression via lipid metabolism remains poorly understood. Therefore, quantification of fatty acids and lipid droplets were performed in vitro and in vivo. The protein-protein interaction was verified by Co-IP technique. The mechanism and underlying signaling pathway of TM7SF2 via CPT1A associated lipid metabolism in cervical cancer development were explored using Western blotting, IHC, colony formation, transwell assay, and wound healing assay. This study reported that overexpression of TM7SF2 increased fatty acids content and lipid droplets both in vivo and in vitro experiments. While knockout of TM7SF2 obviously attenuated this process. Moreover, TM7SF2 directly bonded with CPT1A, a key enzyme in fatty acid oxidation, and regulated CPT1A protein expression in cervical cancer cells. Notably, the proliferation and metastasis of cervical cancer cells were elevated when their CPT1A expression was upregulated. Then, rescue assay identified that CPT1A overexpressed could enhance the cell viability and migration in TM7SF2-knockout cells. Furthermore, depletion of TM7SF2 significantly inhibited WNT and β-catenin proteins expression, which was enhanced by CPT1A-overexpressed. The proliferation and migration of cervical cancer cells were reversed in CPT1A-overexpressed cells with the treatment of MSAB, an inhibitor of Wnt/β-Catenin pathway. This study put forward an idea that TM7SF2-induced lipid reprogramming promotes proliferation and migration via CPT1A/Wnt/β-Catenin axis in cervical cancer, underlying the progression of cervical cancer.

## Introduction

Cervical cancer, the fourth most common cancer, threatens women’s health globally [1]. In China, female cervical cancer accounts for 4.21% of cancer deaths yearly [2]. Cervical cancer is mainly associated with HPV (human papillomavirus) infection, driving several critical molecular events in cervical cancer development [3]. With the promotion of the HPV vaccine globally, cervical cancer has become the most promising malignancy disease to be eliminated [4]. However, significant gaps in diagnosis and treatment between high-income countries and low- and middle-income countries exist persistently [5]. In addition, the constantly changing virus subtypes and drug resistance inducing failure of tumor treatment prevention bring many challenges for research workers [6]. Investigating cervical tumorigenesis mechanisms is therefore important to provide better knowledge to identify potential biomarkers and develop new therapies for cervical cancer.

Transmembrane 7 superfamily member 2 (TM7SF2) is involved in the process of cholesterol biosynthesis [7]. We have previously identified 7 key genes including TM7SF2 that were significantly elevated in HPV-negative cervical cancer cells and tissues [8]. Subsequently, the oncogenic role of the upregulation of TM7SF2 in cervical cancer C33A and SiHa cell lines was also identified by our previous study, in which TM7SF2 regulated proliferation and apoptosis of cervical cancer cells via C-Raf/ERK pathway regulation [9]. However, there is no clear understanding of how TM7SF2 regulates cervical cancer lipid reprogramming.

Over the past decade, the field of cancer metabolism has become a topic of renewed interest. The energy metabolism reprogramming in cancer cells is regarded as a hallmark of cancer [10, 11]. Carnitine palmitoyl transferase 1 A (CPT1A) controls the entry of fatty acids into the mitochondria for oxidation as the first rate-limiting enzyme in fatty acid oxidation [12]. A growing body of evidence associates CPT1A with the malignant progression of various cancers, for CPT1A may participate in the regulation of intracellular neutral lipid content, which is associated with the genesis of aggressive forms of cancers [13]. However, whether TM7SF2 remodels lipid metabolism by mediating CPT1A and thus promotes tumor occurrence have not been unequivocally identified.

In this study, the molecular mechanisms of TM7SF2 via CPT1A in the process of cervical tumorigenesis were explored. The present study demonstrated the content of fatty acids and lipid droplets regulated by TM7SF2. Further, the potential correlation between CPT1A and TM7SF2 was detected. And the expression levels of CPT1A gene in cervical tissues were detected. Moreover, how CPT1A influenced the biological functions including cell viability and migration in cervical cancer was investigated. Furthermore, whether WNT/β-catenin signaling pathway participated in TM7SF2 mediated cervical tumorigenesis via CPT1A was explored. Finally, the potential mechanism of TM7SF2 mediating cervical tumorigenesis via CPT1A was studied. Our results supported a possible new mechanism of lipid reprogramming in cervical cancer progress.

## Results

### Correlation of TM7SF2 and fatty acid metabolism in cervical cancer

**Fig. 1:**
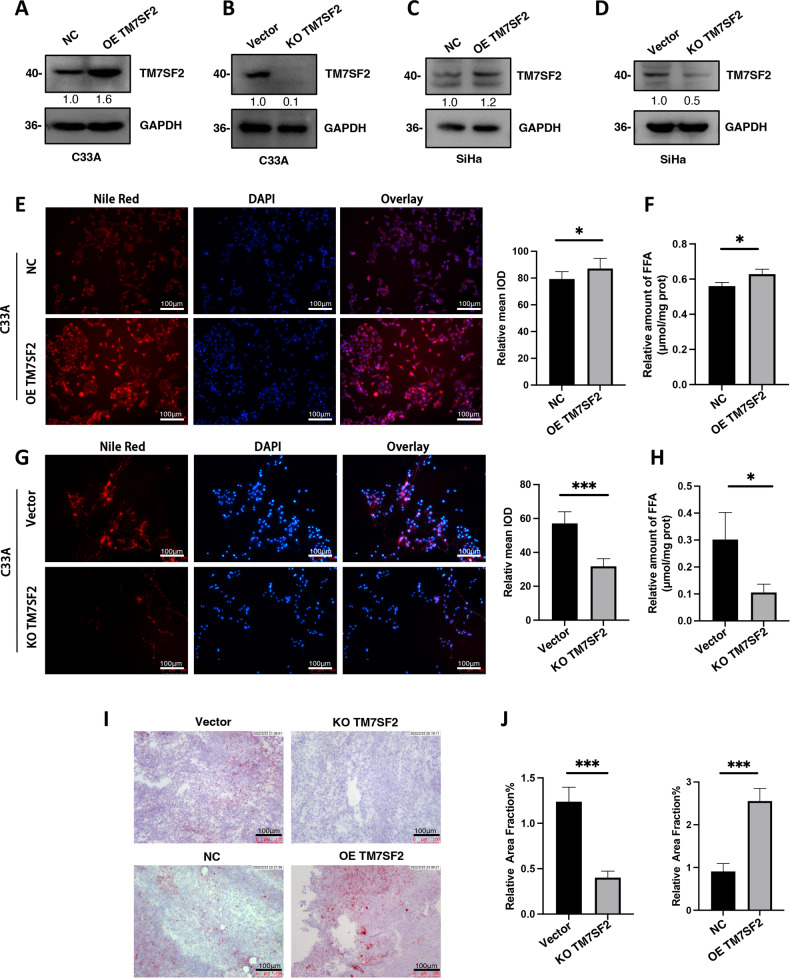
Correlation of TM7SF2 with fatty acid metabolism in cervical cancer. **A** The efficacy of TM7SF2 overexpression in C33A cells. **B** The efficacy of TM7SF2 knock out in C33A cells. **C** The efficacy of TM7SF2 overexpression in SiHa cells. **D** The efficacy of TM7SF2 knock out in SiHa cells. **E** The effect of TM7SF2 upregulated on lipid droplets accumulation in cervical cancer C33A cells (*n* = 5). **F** The effect of TM7SF2 upregulated on isolated fatty acid formation in cervical cancer C33A cells (*n* = 3). **G** The effect of TM7SF2-slienced on lipid droplets accumulation in cervical cancer C33A cell (*n* = 5). **H** The effect of TM7SF2-slienced on isolated fatty acid formation in cervical cancer C33A cells (*n* = 3). **I** Representative images of O-red staining from sections of xenografted tumors established in nude mice. **J** The quantitative results of (**I**) (*n* = 3). *P < 0.05, ***P < 0.001.

The study was initiated by successfully establishing TM7SF2-overexpressed (Fig. 1A, C) and TM7SF2-knockout (Fig. 1B, D) cell lines in C33A and SiHa cervical cancer cell lines. To investigate TM7SF2’s influence on fatty acid metabolism in cervical cancer, the formation of lipid droplets and fatty acids was examined in cervical cancer cells expressing TM7SF2 ectopically. Lipid droplet levels were visualized using Nile red staining, and their presence in the cytoplasm of C33A cells was observed. Elevated TM7SF2 expression led to an accumulation of lipid droplets (Fig. 1E), while TM7SF2 knockout resulted in the reduction of lipid droplets in the C33A cell line (Fig. 1G). Importantly, TM7SF2 overexpression showed a significant positive correlation with elevated fatty acids level in C33A cells (Fig. 1F), whereas depletion of TM7SF2 obviously decreased fatty acids level in comparison with the corresponding control group (Fig. 1H). Additionally, oil red was used to stain the TM7SF2-knockout and TM7SF2-overexpressed xenograft cervical cancer tumors of nude mouse models previously established [9]. The data indicated a decrease in lipid droplet level in stable TM7SF2-knockout tumors, whereas TM7SF2-overexpressed tissues exhibited an accumulation of lipid droplets (Fig. 1I, J).

### Interaction and regulation of CPT1A by TM7SF2 in cervical cancer

**Fig. 2:**
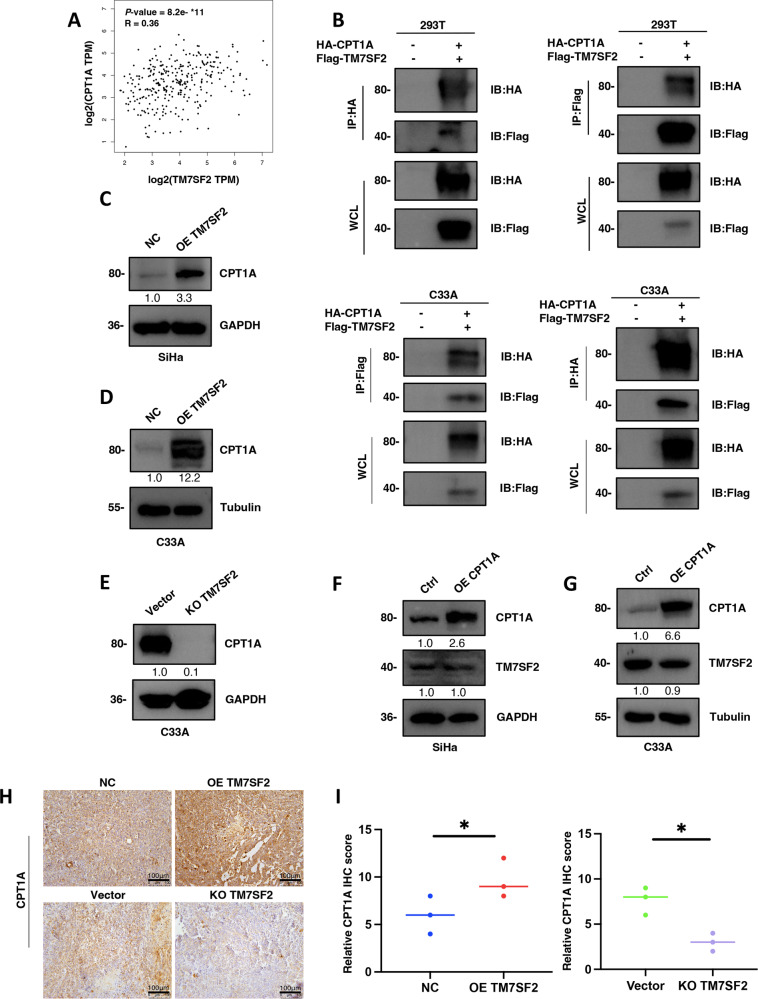
Interaction and regulation of CPT1A by TM7SF2 in cervical cancer. **A** The correlation of TM7SF2 and CPT1A was analyzed by GEPIA database (http://gepia.cancer-pku.cn/index.html). **B** The interaction effect of TM7SF2 protein with CPT1A protein was detected by IP and Co-IP in 293 T and C33A cells. **C** TM7SF2 regulated the expression of CPT1A in SiHa cells. **D**, **E** TM7SF2 regulated the expression of CPT1A in C33A cells. **F**, **G** The expression of TM7SF2 protein in CPT1A upregulated SiHa cells (**F**) and C33A cells (**G**). **H** Representative images of IHC staining for CPT1A in xenografted tumor tissues from TM7SF2-overexpressed and TM7SF2-knockout with corresponding control groups. **I** The quantitative results of (**H**) (*n* = 3). *P < 0.05.

Based on the GEPIA database, TM7SF2 gene expression in cancer was significantly associated with CPT1A (*P* = 8.2 × 10^−11^, *R* = 0.36) (Fig. 2A). As for further confirmation of the interaction between TM7SF2 and CPT1A, exogenous IP and Co-IP assays were conducted, demonstrating the physical interaction of TM7SF2 protein and CPT1A protein both in 293 T and C33A cells (Fig. 2B). Furthermore, TM7SF2-overexpression upregulated CPT1A protein expression level in SiHa and C33A cells compared to corresponding controls (Fig. 2C, D), while TM7SF2-knockout decreased CPT1A protein expression in C33A cells (Fig. 2E). Notably, changes in CPT1A protein expression did not impact TM7SF2 protein expression level when CPT1A was overexpressed in cervical cancer cells, suggesting that CPT1A might function as a downstream effector of TM7SF2 (Fig. 2F, G). In addition, IHC provided further support showing that depletion of TM7SF2 significantly decreased CPT1A protein expression, whereas upregulation of TM7SF2 significantly increased CPT1A protein expression in mouse tumor tissues compared to corresponding controls (Fig. 2H, I). Collectively, these data supported the notion that TM7SF2 regulated CPT1A protein expression by interacting with it.

### Regulation of cell proliferation and migration by CPT1A in cervical cancer

**Fig. 3:**
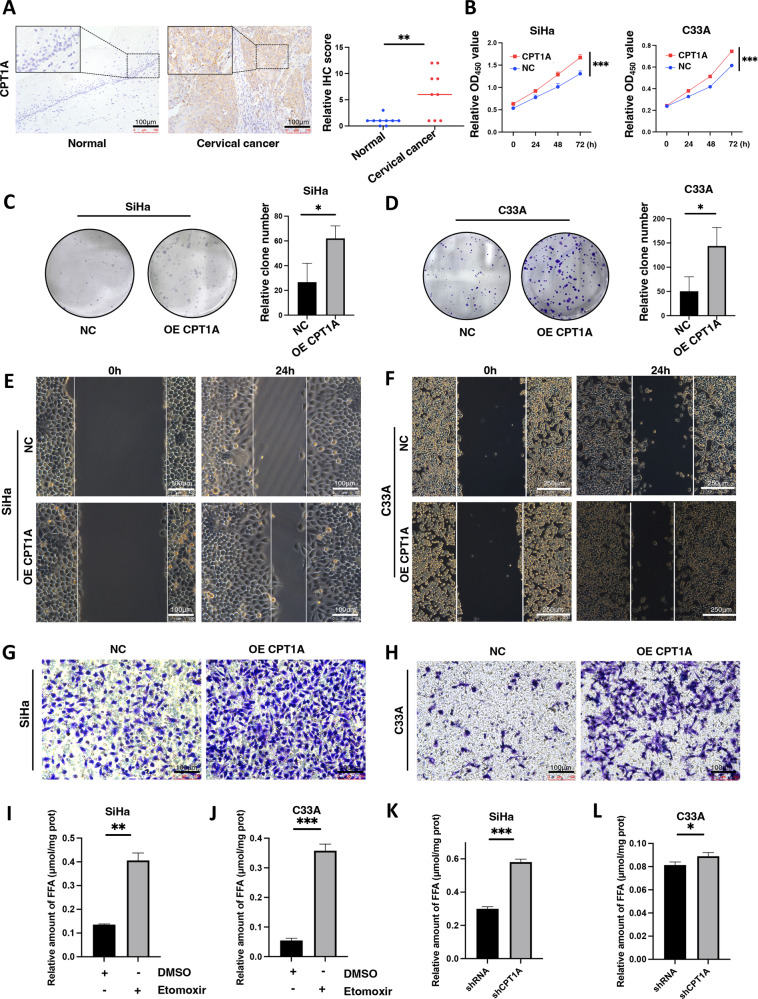
Regulation of cell proliferation and migration by CPT1A in cervical cancer. **A** The protein expression level of CPT1A in human cervical cancer tissues and normal cervix tissues. (*n* = 9) **B** The effect of CPT1A overexpression on cell viability in SiHa and C33A cells (*n* = 6). **C**, **D** The effect of CPT1A overexpression on clonogenicity in SiHa and C33A cells (*n* = 3). **E** The effect of CPT1A overexpression on wound healing assay in SiHa cells. **F** The effect of CPT1A overexpression on wound healing assay in C33A cells. **G** The effect of CPT1A overexpression on migration in SiHa cells. **H** The effect of CPT1A overexpression on migration in C33A cells. **I** The effect of Etomoxir treatment on isolated fatty acid formation in cervical cancer SiHa cells (*n* = 3). **J** The effect of Etomoxir treatment on isolated fatty acid formation in cervical cancer C33A cells (*n* = 3). **K** The effect of CPT1A knockdown on isolated fatty acid formation in cervical cancer SiHa cells (*n* = 3). **L** The effect of CPT1A knockdown on isolated fatty acid formation in cervical cancer C33A cells (*n* = 3). *P < 0.05, **P < 0.01, ***P < 0.001.

Firstly, the protein level of CPT1A was compared in cervical cancer tissues and normal cervix tissues using IHC assay, which revealed a significant increase of CPT1A protein expression in cervical cancer tissues, primarily localized in cytoplasm (Fig. 3A). A CCK-8 assay was conducted to investigate CPT1A whether it affected cervical cancer cell viability in CPT1A-overexpressed SiHa and C33A cells. Cervical cancer cell viability was promoted in both cell lines by upregulating CPT1A (Fig. 3B). Additionally, CPT1A overexpression enhanced cell colony formation in SiHa and C33A cells (Fig. 3C, D). Then, the aspect of CPT1A on cell migration was further studied in cervical cancer cells. As shown in Fig. 3E, F, Fig. S1A–B, overexpression of CPT1A significantly enhanced the migratory capacity, facilitating wound closure in SiHa and C33A cells after 24 h. Moreover, the data from Transwell migration analysis confirmed that CPT1A overexpression significantly promoted cell migration in SiHa and C33A cells comparing with the corresponding control groups (Fig. 3G, H, S1C, D), which suggested that CPT1A regulated the cervical cancer cell proliferation and migration.

Further, the role of CPT1A played in fatty acid oxidation was verified with inhibition of fatty acid oxidation by CPT1A inhibitor Etomoxir. After incubated with Etomoxir with concentration of 50 μM for 48 h, the protein expression of CPT1A in cervical cancer SiHa (Fig. S1E) and C33A cells (Fig. S1F) was inhibited. What’s more, the accumulation of fatty acids was observed in both SiHa (Fig. 3I) and C33A cells (Fig. 3J) with the treatment of Etomoxir, which has been reported in the literature [14]. In addition, to ascertain the function of CPT1A knockdown on fatty acids accumulation of cervical cancer cells, CPT1A-slience SiHa (Fig. S1G) and C33A cells (Fig. S1H) were verified via Western blotting. Compared to the control cervical cancer cells, the enhanced fatty acids were detected in CPT1A-slience SiHa (Fig. 3K) and C33A cells (Fig. 3L).

### Promotion of cell proliferation and migration by TM7SF2 via CPT1A in cervical cancer

**Fig. 4:**
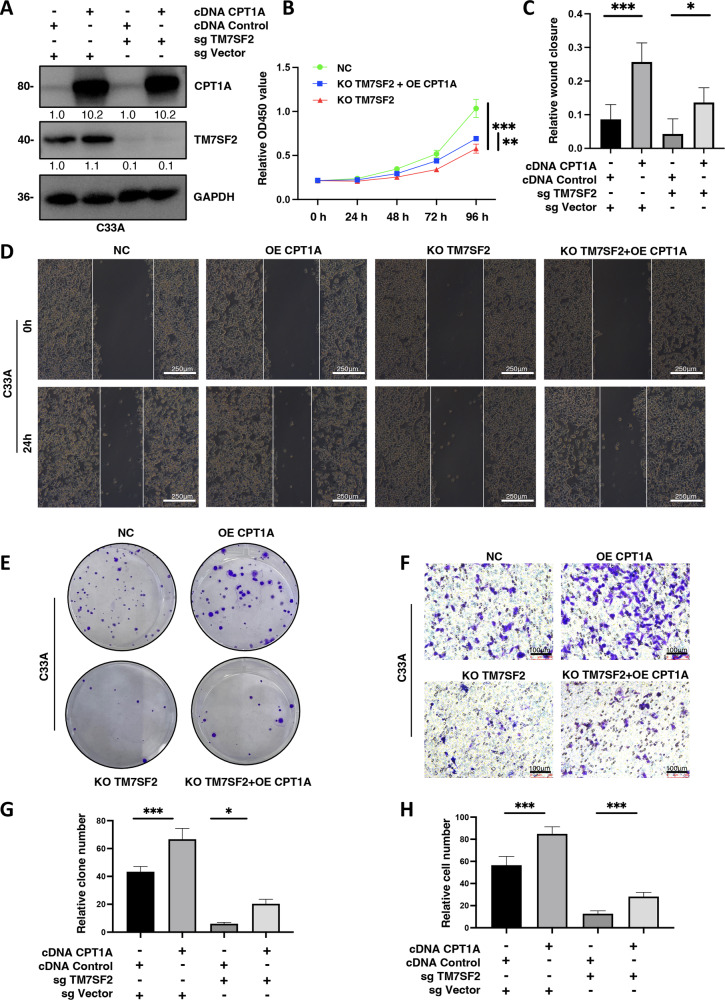
Promotion of cell proliferation and migration by TM7SF2 via CPT1A in cervical cancer. **A** The protein expression of TM7SF2 protein and CPT1A protein in C33A cells. **B** The impact of CPT1A-overexpressed regulated TM7SF2-slienced C33A cells on cell viability (*n* = 6). **C**, **D** The effect of CPT1A-overexpressed regulated TM7SF2-slienced C33A cells on wound healing assay (*n* = 5). **E** The effect of CPT1A-overexpressed regulated TM7SF2-slienced C33A cells on clonogenicity assay. **F** The effect of CPT1A-overexpressed regulated TM7SF2-slienced C33A cells on migration. **G** The quantitative results of **E** (*n* = 3). **H** The quantitative results of **F** (*n* = 3). *P < 0.05, **P < 0.01, ***P < 0.001.

To further validate the role of the TM7SF2-CPT1A axis in cervical cancer, the rescue assays by overexpressing CPT1A in TM7SF2-knockout C33A cells were performed (Fig. 4A). CCK-8 assays indicated accelerated proliferation in TM7SF2-silenced C33A cells with upregulated CPT1A than that in TM7SF2-silenced C33A cells (Fig. 4B). Wound healing assays showed that TM7SF2-knockout reduced wound area closure, while the supplementation of CPT1A enhanced migratory ability (Fig. 4C, D). Additionally, colony formation assays revealed that overexpressed CPT1A in TM7SF2-silenced C33A cells partially rescued proliferation ability (Fig. 4E, G), highlighting the role of CPT1A in promoting proliferation in TM7SF2-knockout C33A cells. As illustrated in Fig. 4F, H, the ability of migration in C33A cells also enhanced when CPT1A upregulated in TM7SF2-knockout cells than that in only TM7SF2-knockout C33A cells. These results implied that TM7SF2 was bound with CPT1A to promote cell proliferation and migration in cervical cancer.

### Regulation of WNT/β-catenin signaling by TM7SF2 via CPT1A in cervical cancer

**Fig. 5:**
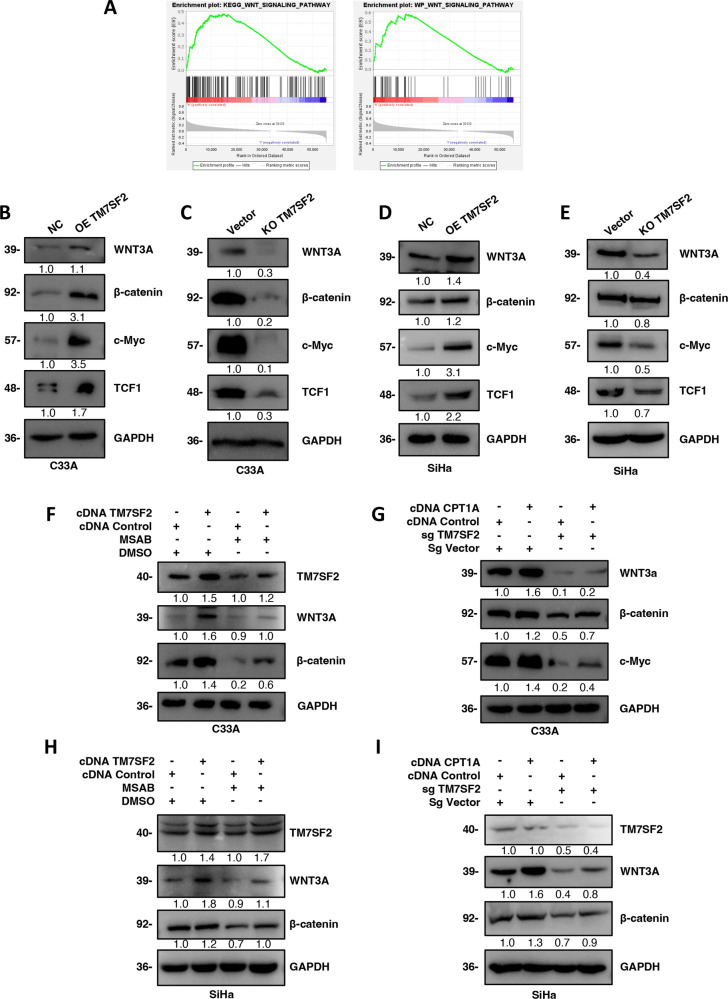
Regulation of WNT/β-catenin signaling by TM7SF2 via CPT1A in cervical cancer. **A** The gene set enrichment analysis (GSEA) was exerted to explore the signaling pathways related to CPT1A. **B**, **C** Western blotting analysis of WNT/β-catenin signaling pathway-related protein expression after TM7SF2 overexpression and TM7SF2 knockdown in C33A cells. **D**, **E** Western blotting analysis of WNT/β-catenin signaling pathway-related protein expression after TM7SF2 overexpression and TM7SF2 knockdown in SiHa cells. **F** Western blotting analysis of WNT3A and β-catenin protein expression in TM7SF2-overexpressed C33A cells after MSAB treatment. **G** Western blotting analysis of the effect of CPT1A-overexpressed regulated the protein expression of WNT3A, β-catenin and c-Myc in TM7SF2-slienced C33A cells. **H** Western blotting analysis of WNT3A and β-catenin protein expression in TM7SF2-overexpressed SiHa cells after MSAB treatment. **I** Western blotting analysis of the effect of CPT1A-overexpressed regulated the protein expression of WNT3A, β-catenin in TM7SF2-slienced SiHa cells.

To gain insights into the underlying mechanisms of TM7SF2 in cervical cancer, we conducted a GSEA analysis, which revealed a significant association between CPT1A and the WNT signaling pathway (Fig. 5A, Supplementary Table 1). Subsequent experiments confirmed proteins such as WNT3A, β-catenin, c-Myc and TCF1, typical proteins in the WNT/β-catenin signaling pathway, were upregulated in TM7SF2-overexpressed C33A and SiHa cell lines (Fig. 5B, D). In contrast, these proteins were downregulated in the TM7SF2-knockout C33A and SiHa cell lines (Fig. 5C, E). MSAB, which could inhibit the WNT/β-catenin signaling pathway, was also employed to study deeper into this pathway. Treatment with MSAB attenuated the expression of WNT3A and β-catenin proteins in the WNT/β-catenin signaling pathway compared to control groups without MSAB treatment. Interestingly, TM7SF2 overexpression partially restored the WNT3A and β-catenin protein expression (Fig. 5F, H). Lastly, upregulation of CPT1A partially reversed the suppression of WNT3A, β-catenin, and c-Myc protein expression mediated by TM7SF2-knockout (Fig. 5G, I), suggesting that TM7SF2 regulated the WNT/β-catenin signaling via CPT1A in cervical cancer cells.

### Promotion of cell proliferation and migration by CPT1A via WNT/β-catenin signaling in cervical cancer

**Fig. 6:**
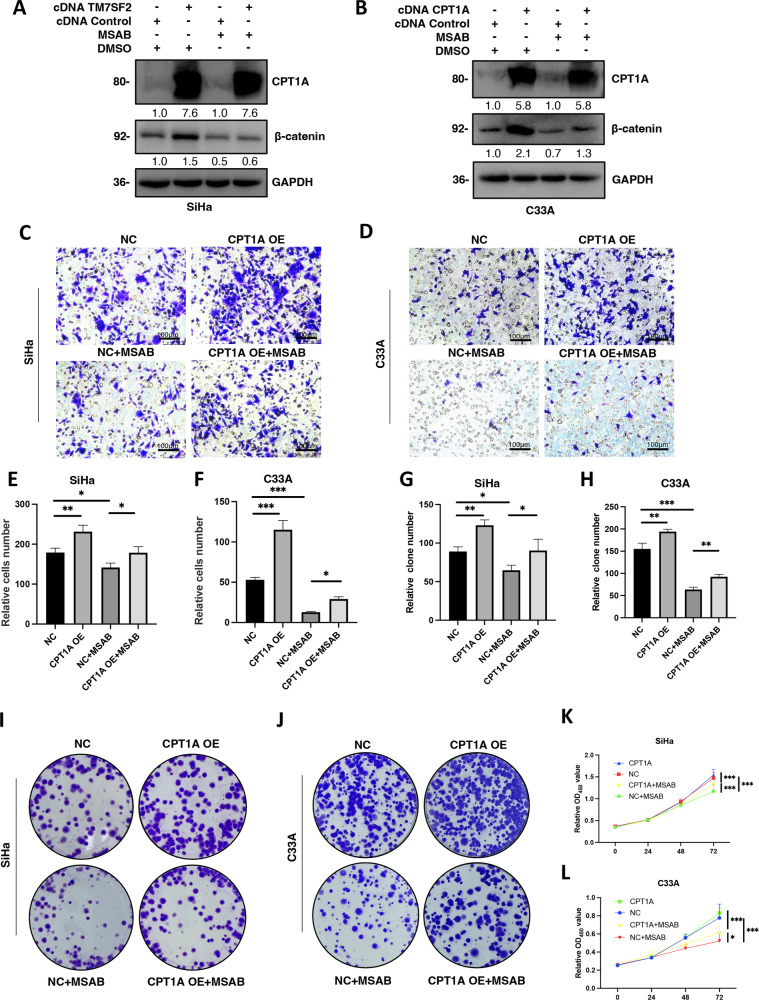
Promotion of cell proliferation and migration by CPT1A via WNT/β-catenin signaling in cervical cancer. **A** The protein expression of CPT1A protein and β-catenin in SiHa cells. **B** The protein expression of CPT1A protein and β-catenin in C33A cells. **C** The effect of MSAB treatment regulated CPT1A-overexpressed SiHa cells on migration assay. **D** The effect of MSAB treatment regulated CPT1A-overexpressed C33A cells on migration assay. **E** The quantitative results of **C** (*n* = 3). **F** The quantitative results of **D** (*n* = 3). **G** The quantitative results of **I** (*n* = 3). **H** The quantitative results of **J** (*n* = 3). **I** The effect of MSAB treatment regulated CPT1A-overexpressed SiHa cells on clonogenicity assay. **J** The effect of MSAB treatment regulated CPT1A-overexpressed C33A cells on clonogenicity assay. **K** The effect of MSAB treatment regulated CPT1A-overexpressed SiHa cells on cell viability (*n* = 6). **L** The effect of MSAB treatment regulated CPT1A-overexpressed C33A cells on cell viability (*n* = 6). *P < 0.05, **P < 0.01, ***P < 0.001.

Intend to explore the role of CPT1A in regulation of the WNT/β-catenin pathway, the inhibitor of WNT/β-catenin was used in CPT1A-overexpressed cervical cancer cell lines. Firstly, the activity of β-catenin was inhibited with MSAB treatment compared with the groups without MSAB treatment in SiHa and C33A cell lines. CPT1A overexpressed partially recovered the expression of β-catenin in SiHa cells treated with MSAB, which was also consistent in C33A cells (Fig. 6A, B). Furthermore, the decline of cell migration ability with MSAB treatment was partially counteracted when CPT1A overexpression. After the treatment with MSAB, the migratory ability of CPT1A-overexpressed SiHa and C33A cells was reduced compared with those cells without MSAB treatment, but it was enhanced than that in SiHa (Fig. 6C, E) and C33A (Fig. 6D, F) control cells with MSAB treatment. Additionally, cell colony formation and viability abilities were reversed in CPT1A-overexpressed SiHa cells (Fig. 6G, I, K) and C33A cells (Fig. 6H, J, L) with the use of MSAB correspondingly.

## Discussion

TM7SF2, initially recognized for its involvement in cholesterol metabolism [7], has emerged as a multifunctional protein with diverse roles in various physiological and pathological processes [15, 16]. Lei et al. [17] perceived TM7SF2’s effect in the burn wound healing process assessed in SD rats that underwent electrical burns, confirming that it took part in the process of burn wound repairing by working with LC3‑II and Beclin1. Also, TM7SF2’s involvement in tumorigenesis, particularly in differentiating non-aggressive and aggressive follicular carcinomas in the thyroid, underscored its potential as a diagnostic biomarker in cancer management [18]. Our study built upon this expanding understanding of TM7SF2’s functions by shedding light on its significant contributions to cervical cancer progression. Our previous findings elucidated a novel mechanism of TM7SF2 expediting the disease progression that TM7SF2 overexpression promoted cervical cancer cell proliferation and invasion in vitro and in vivo [9]. To determine whether TM7SF2 affected the metabolism of fatty acids in facilitating neoplastic progression, detailed investigations into intracellular fatty acids level and lipid droplet accumulation were conducted. This study found that TM7SF2 could promote the fatty acids level and lipid droplet accumulation in cervical cancer, implying the novel role of TM7SF2 in the lipid reprogramming of tumorigenesis.

Tumor progression is profoundly affected by alterations of intracellular metabolic intermediates and reprogramming of cancer-related metabolism [19, 20]. A comprehensive understanding of lipid metabolism encompasses both fatty acid synthesis and fatty acid oxidation. The balance between these processes is contingent upon the status of nutritional and tissue mitochondrial metabolism in normal cells [12]. However, within the context of cancer, fatty acids assume critical roles in membrane formation, signaling pathways, and post-translational modifications of proteins, all of which are pivotal for cancer cell proliferation [21]. Using plasma samples from women at high risk of breast cancer, whole metabolite and protein profiling could be performed. Madak-Erdogan et al. [22] have reported that there was a correlation between free fatty acids in the plasma and increased proliferation and aggressiveness in ER+ breast cancer cells, in which fatty acids activated both ERα and mTOR pathways and influenced mammary epithelial cell tumorigenicity and aggressiveness. It was reported that lipid droplets accumulation promoted cell metastasis in cervical cancer modulated by FASN [23]. Another study revealed the mechanism that FABP5 promoted fatty acid synthesis and lipolysis, which led to an increase in intracellular fatty acids thus inducing lymph node metastasis in cervical cancer [24]. Beyond that, fatty acid activation is the first stage of fatty acid oxidation. Fatty acid oxidation acts to feed the tumor growth by increasing ATP and NADPH production to get out of the conditions of metabolic stress [12]. CPT1A, a rate-limiting enzyme in fatty acid oxidation, undergoes upregulation in various tumor types [25–27]. Its overexpression in cancer cells can activate fatty acid oxidation, thereby enhancing ATP production. Tan et al. [14] found a hyperactivate fatty acid oxidation activity in radiation-resistant nasopharyngeal cancer with the up-regulated CPT1A in these cells, which mediated radiation resistance by facilitating fatty acids trafficking. Similarly, CPT1A expression level was elevated in colorectal cancer, particularly within metastatic sites. In-depth investigations have revealed that CPT1A-mediated fatty acid oxidation activation enabled colorectal cancer cells to resist anoikis [25]. Consistent with these findings, CPT1A was found to be upregulated in cervical cancer in this study, and its overexpression promoted the migration and proliferation of cervical cancer cells in vitro. Interestingly, more free fatty acids, lipid droplets, and increased CPT1A-mediated activity fatty acid oxidation were found in our study. Similarly with our findings, You et al. [28] reported the Paclitaxel-tolerant persisted cancer cells with more free fatty acids, lipid droplets, and fatty acid oxidation than their parental cells which showed sensitivity to ferroptosis inducers with PGRMC1 upregulation. Furthermore, free fatty acid, lipid droplets and lipid deposition were modestly increased by inhibiting fatty acid oxidation and activating fatty acid synthesis with Etomoxir treatment in Paclitaxel-tolerant persisted cancer cells. Another important finding was the lipid-dependent metabolic reprogramming triggered by antiangiogenic drug which then caused antiangiogenic drug resistance. The tumor hypoxia induced by antiangiogenic drug initiated the fatty acid oxidation reprogramming but at the same time increased the free fatty acid intake and stimulated cancer cell proliferation [29]. Mechanically, cancer cells acquired exogenous fatty acids released by cancer-associated adipocytes through cell surface fatty acid translocase such as CD36, and then stored excessive energy as lipid droplets that could be further broken down into free fatty acids entering fatty acid oxidation [30]. Next, excessive free fatty acids might trigger the activation of CPT1A in cancer cells [31]. In support of this view, combination of antiangiogenic drug with a β-oxidation inhibitor such as the CPT1 inhibitor etomoxir produced greater anticancer effects in an animal model of hepatocellular cancer grown in liver [30]. In the present study, lipid metabolic reprogramming was triggered by TM7SF2 upregulation, that excessive lipid droplets could break down free fatty acids which could transported into the mitochondria accelerating fatty acid oxidation.

Crucially, our research identified a central role for TM7SF2 in mediating CPT1A’s function in cervical cancer. Studies demonstrated that TM7SF2 directly interacted with CPT1A, thereby regulating its protein expression. Conversely, CPT1A-overexpressed did not influence the protein expression of TM7SF2. These results supported the hypothesis that CPT1A might function as a downstream effector of TM7SF2 and TM7SF2-mediated CPT1A regulation synergistically influenced the progression of cervical cancer. Splicing of mRNA, promoter activity and protein post-translational modification may influence the protein expression. As a physical link between proteins and their partners, protein-protein interactions (PPIs) can show heterogeneities and complexity in macromolecular structures, including long chains, multicomponent complexes, or protein dimers. A PPI system controls the activity of proteins by connecting enzymes to their substrates [32]. Evidences have been mounting that the formation of protein-protein complexes is critical for efficient post-translationally modified peptide biosynthesis [33]. It has been reported that hyperacetylation of CPT1A in mice contributed to reduced CPT1A enzymatic activity in isolated mitochondria from the mice by fructose or glucose supplementation on the chow diet [34]. Therefore, it is reasonable to assume that TM7SF2 directly interacted with CPT1A leading to post-translational modification in CPT1A protein and regulating its function which need thoroughly researched.

Evolutionarily conserved and ancient, the WNT signaling contributes to tumorigenesis in different organs and affects the tumor cells and immune microenvironment, which has been proved [35]. Numerous cancers were found to exhibit mutations in the WNT/β-catenin pathway which were tightly linked with the progression of malignant diseases, poor prognoses and even an increase in cancer-related deaths [36]. Additionally, it was highly linked to the development of adipose tissue. Human and mouse genetic studies suggested that WNT signaling played a central role in the distribution of body fat, obesity and dysfunction of metabolism [37]. Notably, Xiong et al. [38] reported that the knockdown of CPT1A significantly decreased downstream gene expression associated with WNT/β-catenin signaling in colon cancer stem cells. Mechanically, CPT1A-dependent fatty acid oxidation activated the acetylation and nuclear translocation of β-catenin. In our finding, GSEA analysis revealed a correlation between CPT1A and the WNT/β-catenin signaling pathway in cervical cancer. Furthermore, TM7SF2 overexpression promoted WNT/β-catenin signaling and downstream genes which were inhibited by the TM7SF2-knockout cell line. MSAB was used for further investigation of TM7SF2’s impact on WNT/β-catenin signaling. MSAB could inhibit the increased protein expression of WNT3A and β-catenin resulting from TM7SF2 overexpression. Our additional experiments confirmed that TM7SF2 regulated WNT/β-catenin signaling via CPT1A. The upregulation of CPT1A partially reversed the inhibition of WNT3A, β-catenin, and downstream genes caused by TM7SF2 knockout. These findings underscored the pivotal importance of investigating the crosstalk between WNT signaling, lipid reprogramming and tumorigenesis.

**Fig. 7:**
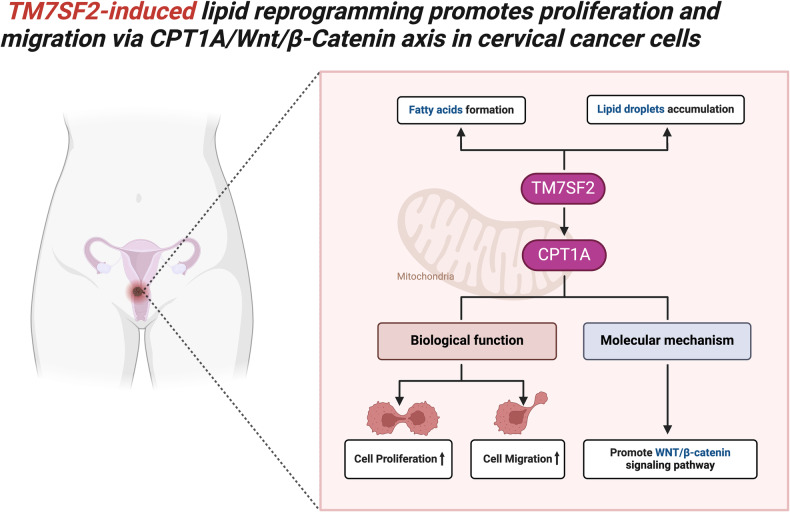
Regulation of WNT/β-catenin signaling by TM7SF2 via CPT1A in cervical cancer. A schematic diagram displays TM7SF2 overexpression promotes the progression of cervical cancer and upregulates WNT/β-catenin signaling via CPT1A.

Generally, our study highlights the significance of TM7SF2 in cervical cancer tumorigenesis through its role in regulating lipid metabolism. Mechanistically, TM7SF2 promotes the accumulation of lipid droplets and increases intracellular fatty acids. Additionally, TM7SF2 directly interacts with and activates CPT1A, a key enzyme in fatty acid oxidation. TM7SF2 further facilitates neoplastic progression by enhancing the WNT/β-catenin signaling pathway through the regulation of CPT1A (Fig. 7). These findings underscore the critical role of TM7SF2 in cervical cancer and its involvement in lipid metabolism regulation. The potential therapeutic targeting in the context of lipid reprogramming in cervical cancer warrants further investigation.

However, there are still some limitations in this study. The evidence is inadequate in how TM7SF2 regulates CPT1A protein expression via interacting with it. In the future, a systematic study of these will be conducted which may make some interesting findings.

## Materials and methods

### Cell culture

Human cervical cancer cell lines C33A and SiHa were obtained from the American Type Culture Collection (ATCC, USA). In both cell lines, Dulbecco’s modified Eagle’s medium (DMEM, Meilunbio, China) with 10% fetal bovine serum (FBS, Gibco, USA) was used as a culture medium under 5% CO_2_ at 37 °C.

### CRISPR-Cas9 knockout

TM7SF2-knockout (KO-TM7SF2) cells were established using the CRISPR-Cas9 system. Specific sequences could refer to the previous study [9]. Cervical cancer cell C33A was transfected with sgTM7SF2 for 48 h. After selected by puromycin, the number of cells was counted by cell-counting and diluted into a single cell. A single cell was then seeded into each well of the 96-well plate. Finally, western blotting assay was performed to verify the knockdown efficacy.

### Cell transfection and plasmid construction

All the overexpression cDNA including OE-TM7SF2 (TM7SF2-overexpressed) and OE-CPT1A (CPT1A-overexpressed) and knockdown shRNA including shCPT1A were designed and synthesized by Youbio biosciences lnc (Changsha, China). The TM7SF2 and CPT1A cDNA sequence were cloned into pcDNA3.1-3xFlag-C vector (Flag-TM7SF2) and pcDNA3.1-3xHA-C vector (HA-CPT1A) respectively. The shCPT1A sequence were cloned into pLVX-shRNA-Puro. And the plasmid was transfected into cancer cells using Lipofectamine (Meilunbio, China, MA0672) at 1:1000. In brief, DNA dilution was prepared by mixing Opti-MEM Serum Medium (Gibco, USA), relative cDNA and Lipofectamine. After 20 min of quiescence at room temperature, the mixture was added to the target cells. After prepared by mixing Opti-MEM Serum Medium (Gibco, USA), relative shRNA, lentivirus packaging helper plasmids and Lipofectamine, the mixture was added to the 293 T cells. Then, collected the medium with lentiviral particles and infected the target cells after culturing 48 h and 72 h. Western blotting assay was performed to verify the transfection efficacy.

### Antibodies and reagents

Primary antibodies containing rabbit anti-human β-catenin (#8480), anti-c-Myc (#18583), anti-TCF1 (#2203), anti-GAPDH (#5174), and anti-Tubulin (#2146) antibodies were bought from Cell Signaling Technology (Danvers, USA). Sigma-Aldrich (St. Louis, MO, USA) provided rabbit anti-Flag (F7425) and anti-HA (H6908) antibodies. Rabbit anti-human CPT1A (ab220789) antibody was obtained from Abcam (Cambridge, UK). Rabbit anti-human TM7SF2 (orb4574) antibody was obtained from Biobyt (UK). Rabbit anti-human WNT3A (A0642) antibody was obtained from ABclonal (China). The inhibitor of WNT/β-catenin signaling named MSAB was obtained from MedChemExpress (MCE, NJ, USA), which was dissolved into dimethyl sulfoxide (DMSO) and stored at −20 °C with a concentration of 10 mM. For in vitro assays, a final concentration of 5 μM was used. The inhibitor of fatty acid oxidation named Etomoxir was obtained from MedChemExpress (MCE, NJ, USA), which was dissolved into dimethyl sulfoxide (DMSO) and stored at −20 °C with a concentration of 10 mM. For in vitro assays, a final concentration of 50 μM was used.

### Animal assay and tissue slice preparation

The in vivo xenograft tumor tissues were obtained from a previous study established by our group [9]. In brief, TM7SF2-knockdown or TM7SF2-overexpressed tumor models with corresponding control tumor models were established by injecting C33A cells subcutaneously into each BALB/c nude mouse. For the IHC assay, all the tissues were paraffin-embedded using tissue processor (Taiva, China) after formalin-fixed overnight. And then sliced into 5 μm using an Ultra-Thin Microtome (Leica, Japan). The temperature was controlled at about 37–40 °C, which was conducive to the spread of paraffin sections. The pasted sections were placed in a 60 °C constant temperature box and dried for 2 h before usage. For specific dying assay, freezing sections were used. Xenograft tumor samples were frozen immediately after dissection. Then the frozen samples were cut into 10 μm using a freezing microtome (Thermo, USA), affixed to microscope slides, and dipped in formalin at 4 °C for 30 min before usage.

### Immunohistochemistry (IHC)

The cervical cancer case tissues and normal cervix case tissues were obtained from the Second Affiliated Hospital of Wenzhou Medical University. The xenograft tumor tissues were obtained from the previous study [9]. The section preparation was mentioned above. A graded ethanol solution was then used to rehydrate sections after deparaffinization with xylene. After microwaved for antigen retrieval, the slices were incubated with rabbit anti-human CPT1A antibody (1:500, Abcam, ab220789) overnight at 4 °C. After washed with phosphate buffer solution (PBS), the secondary antibody was used to incubate with sections for 30 min. The sections were then stained using 3,3’-diaminobenzidine (DAB) solution (Zhongshan Golden Bridge Biotechnology Co., Ltd, China). Finally, hematoxylin was taken to counterstain the cell nuclei for 20 s. Scores for IHC were calculated by multiplying the staining intensity score (0 = non-staining, 1 = weak staining, 2 = moderate staining, and 3 = strong staining) and the staining area score (0 = non-staining, 1 = less than 25%, 2 = between 26 and 50%, 3 = between 51 and 75% and 4 = between 75 and 100% stained cells). The final immunostaining scores were evaluated for statistical analysis.

### Western blotting

Cell Radioimmunoprecipitation assay buffer (RIPA) lysis buffer (P0013B, Beyotime, Shanghai) containing phenylmethanesulfonylfluoride (PMSF) was used to lyse the cells. Afterward, 20–40 g of proteins were electrophoresed on sodium dodecyl sulfate-polyacrylamide gel electrophoresis (SDS/PAGE), which were transferred onto the polyvinylidene fluoride (PVDF) membranes subsequently. The corresponding primary antibodies were taken to incubate with membranes overnight after blocking in nonfat milk for 2 h. Followed by a second antibody (1:3000, Biosharp, China) incubation at room temperature for an hour. The primary antibodies were listed as follows: anti-TM7SF2 (1:1000), anti-CPT1A (1:1000), anti-GAPDH (1:3000), anti-Tubulin (1:3000), anti-Flag (0.8 mg/mL), anti-HA (0.8 mg/mL), anti-WNT3A (1:1000), anti-β-catenin (1:1000), anti-c-Myc (1:1000) and anti-TCF1 (1:1000). Protein bands were observed by adding enhanced chemiluminescence (ECL, meilunbio, China) reagent on the bands. Finally, ImageJ software was used to calculate relative protein expression.

### Cell proliferation assay

A density of 8000 cells per well was used for C33A cells and 2000 cells per well for SiHa cells into 96-well plates. After incubated for 24 h, 48 h and 72 h, CCK-8 solution (Beyotime, China) was added to per well (10 μL/well). The Microplate readers (Thermo, USA) detected the plates at absorbance 450 nm after 3 h of incubation at 37 °C.

### Colony formation assay

C33A cells or SiHa cells were seeded into 6-well plates with 800 cells or 200 cells each well respectively and cultured in a 37 °C incubator for 2 weeks. Afterward, the media was discarded, and the cells were washed with PBS. The cells were fixed for 30 min with 4% paraformaldehyde and then stained for 10 min with crystal violet (Solarbio, China). Finally, the number of cell colonies was calculated.

### In vitro wound scratch healing assay and transwell assay

As for the wound healing assay, once the confluence of C33A cells reached 90%, a wound by scratching it with a pipette tip was created. The wound area was photographed by a microplate reader right away and the day after 24 h. The relative wound closure rates were calculated by ImageJ software. The migratory behavior of cervical cancer cells was evaluated using Transwell chambers (Corning, USA). The upper chamber was seeded with cells grown in non-serum culture medium, meanwhile filling the lower chamber with 1 mL DMEM medium containing FBS. After 24 h, 0.1% crystal violet solution (Solarbio, China) was served to colorate the cells in the chamber substrate for 15 min. A microscope (Leica, Japan) was used to count the number of cells that migrated.

### Quantification of lipid metabolites

The Free Fatty Acid Assay Kit (BC0595, Solarbio, China) was used to perform the quantification of fatty acids. The manufacturer’s instructions were followed during the assay. In brief, the cells were resuspended in extraction solution and centrifuged at 8000 × *g* for 10 min at 4 °C. After determining the protein concentration, the supernatant was mixed with the working solution and then centrifuged the mixture for 10 min at 3000 × *g*. At last, a microplate reader (Thermo, USA) was used to quantify the supernatant at 550 nm.

### Immunoprecipitation (IP) and co-immunoprecipitation (Co-IP)

Cells were lysed using NP-40 buffer (P0013F, Beyotime, Shanghai) supplemented with phosphatase inhibitors cocktail (Bimake, USA) and protease inhibitors cocktail (Bimake, USA). The lysates were incubated with 20 μL HA/Flag beads (Bimake, USA) overnight at 4 °C. After being washed 4 times with NETN buffer (NP-40, EDTA, Tris.8.0 and NaCl), HA/Flag beads were mixed with loading buffer and heated denaturing the proteins at 100 °C. Finally, Western blotting assay was next performed.

### Nile red staining and Oil red O staining

Nile red staining was used to quantify cell lipid droplets. Transfected cervical cancer (TM7SF2-overexpressed or TM7SF2-silenced) cells were cultured in 6-well plates that had been pre-coated with sterile glass coverslips. After the cell layer reached 40%, the cells were fixed with 4% formaldehyde for 30 min. Next, Nile red staining solution (ABmole, USA) co-incubated with cells for 20 min and then cells were stained with DAPI (Abcam, USA) at room temperature. Oil Red O (Solarbio, China) staining was performed for tissue lipid droplet quantification. In this assay, frozen sections from mice xenograft tumor tissues were used. A specific slice process was mentioned above. After being fixed in 4% formaldehyde for 30 min and rinsed with 60% isopropanol, the sections were stained with Oil Red O working solution for 15 min and dyed with hematoxylin for 1 min. All pictures were taken with a Leica microscope.

### Gene set enrichment analysis (GSEA) and gene expression profiling integrative analysis (GEPIA)

GSEA analysis [39] of CPT1A was conducted using GSEA 4.3.2 software with gene sets c2.cp.kegg.v2023.1.Hs_symbols.gmt and c2.cp.wikipathways.v2023.1.Hs_symbols.gmt. The TCGA-CESC dataset comprised 306 cervical cancer samples, selected based on their RNA expression profiles. Samples were classified into either ‘high expression’ or ‘low expression’ groups based on the median expression value of CPT1A. Statistical significant was determined when the enrichment score (ES) was >0.4 and false discovery rate (FDR) was <0.05. Based on the above criteria, the activated and inhibited pathways after CPT1A overexpression in cervical cancer were obtained using KEGG or Wiki gene sets, respectively (Supplementary Tables 1). The GEPIA server (http://gepia.cancer-pku.cn/index.html) [40] was intended to analyze the potential correlation of TM7SF2 and CPT1A gene expression levels. Statistical significance was determined with a threshold of *P* *<* 0.05.

### Statistical analysis

Statistical analysis was performed by SPSS 21.0 (SPSS Inc., Chicago, IL, USA) and visualized using Prism 9.0 software (GraphPad). At first, normal distribution of the data was assessed. Levene’s Test was employed to test equality of variance among groups. In the case of two groups, the Student’s *T* Test was used to compare data that followed a normal distribution with equal variance, while the Welch Test was applied for data that confirmed to a normal distribution with unequal variance. ANOVA Test was used for statistical comparisons among multiple groups conforming to normal distribution, and Fisher’s least significant difference Test was used for further pairwise comparisons. the Mann–Whitney Test was applied for non-normally distributed data. A statistical significance level of *P* < 0.05 was considered.

### Supplementary information

Supplementary information

supplemental figure 1

Supplementary table 1

Original Data File

## Data Availability

The data that support the findings of this article are available from the corresponding author upon reasonable request.
