# Supplementary material for: TM7SF2-induced lipid reprogramming promotes cell proliferation and migration via CPT1A/Wnt/β-Catenin axis in cervical cancer cells
Source: Cell Death Discov. 2024 May 1;10:207. doi: 10.1038/s41420-024-01975-8 (PMC11063194; doi:10.1038/s41420-024-01975-8)
Supplement: Supplementary file 1 — Supplementary information [file 41420_2024_1975_MOESM1_ESM.docx]

Supplementary figure 1. (A) The quantitative results of Fig. 3E (n=5). (B) The quantitative results of Fig. 3F (n=5). (C) The quantitative results of Fig. 3G (n=3). (D) The quantitative results of Fig. 3H (n=3). (E) The protein expression of CPT1A protein in SiHa cells. (F) The protein expression of CPT1A protein C33A cells. (G) The protein expression of CPT1A protein in SiHa cells. (H) The protein expression of CPT1A protein C33A cells. ****P* < 0.001.

Supplementary table 1: Gene-Set Enrichment Analysis (GSEA) of Pathways Significantly Represented of CPT1A in cervical cancer.
