# Supplementary figures and images for: TM7SF2-induced lipid reprogramming promotes cell proliferation and migration via CPT1A/Wnt/β-Catenin axis in cervical cancer cells
Source: Cell Death Discov. 2024 May 1;10:207. doi: 10.1038/s41420-024-01975-8 (PMC11063194; doi:10.1038/s41420-024-01975-8)

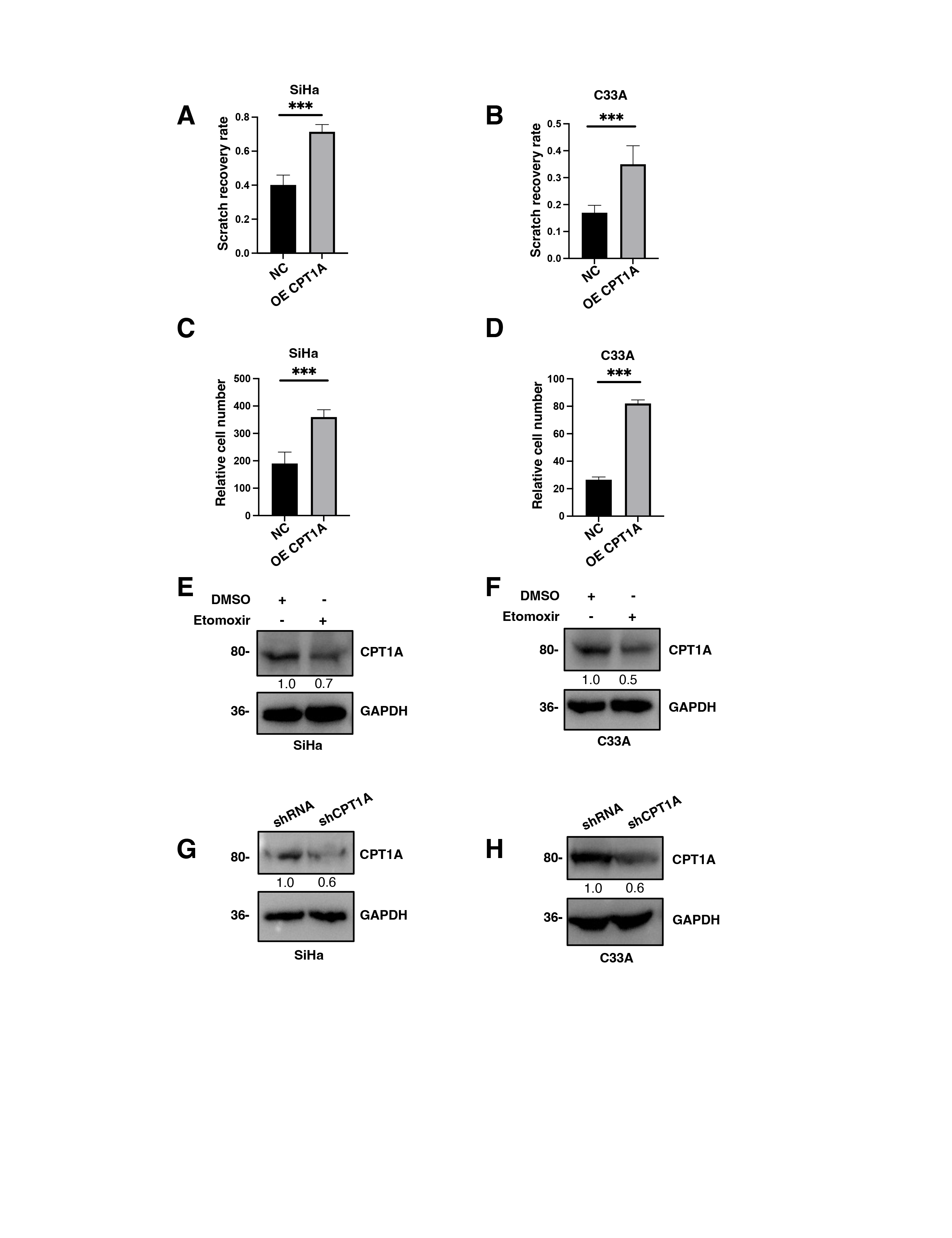

Supplement: Supplementary file 2 — supplemental figure 1 [file 41420_2024_1975_MOESM2_ESM.png]
