# Supplementary material for: TM7SF2-induced lipid reprogramming promotes cell proliferation and migration via CPT1A/Wnt/β-Catenin axis in cervical cancer cells
Source: Cell Death Discov. 2024 May 1;10:207. doi: 10.1038/s41420-024-01975-8 (PMC11063194; doi:10.1038/s41420-024-01975-8)
Supplement: Supplementary file 4 — Original Data File [file 41420_2024_1975_MOESM4_ESM.docx]

**Figure 1A**


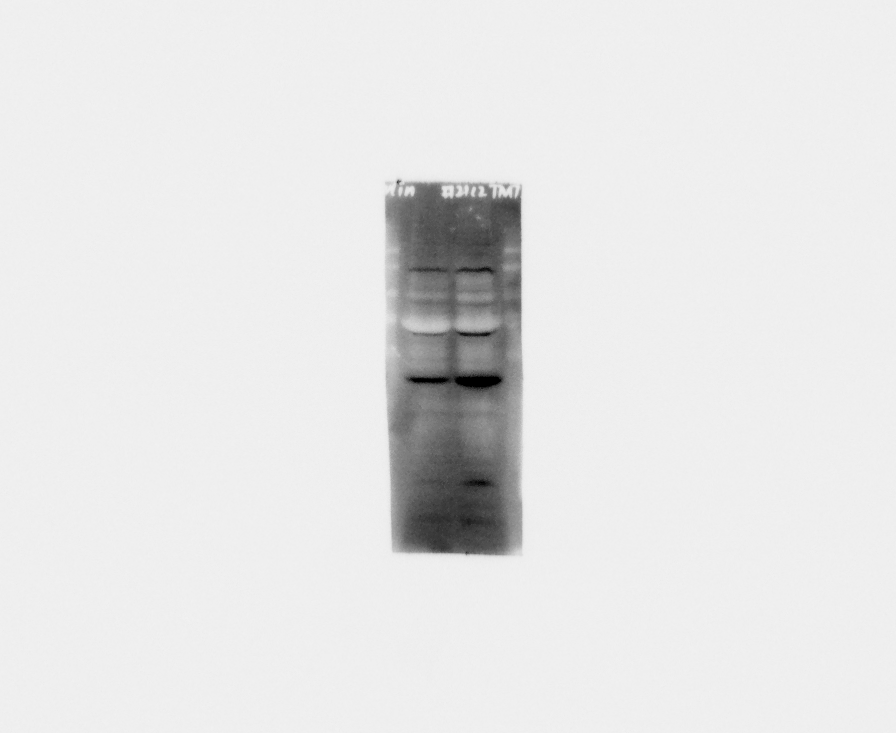

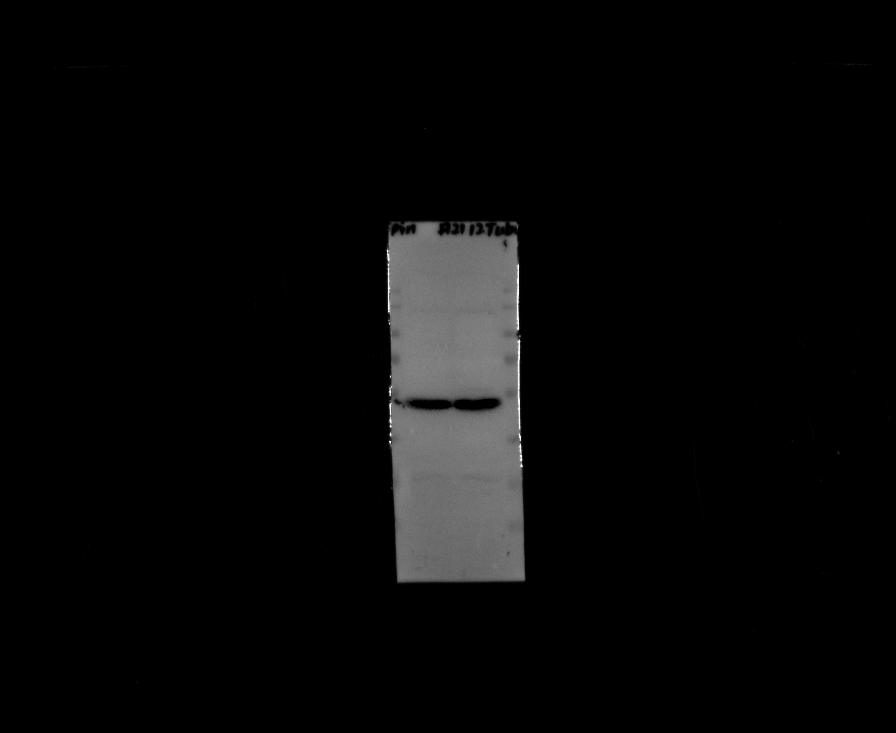


**GAPDH**

**TM7SF2**

**Figure 1B**


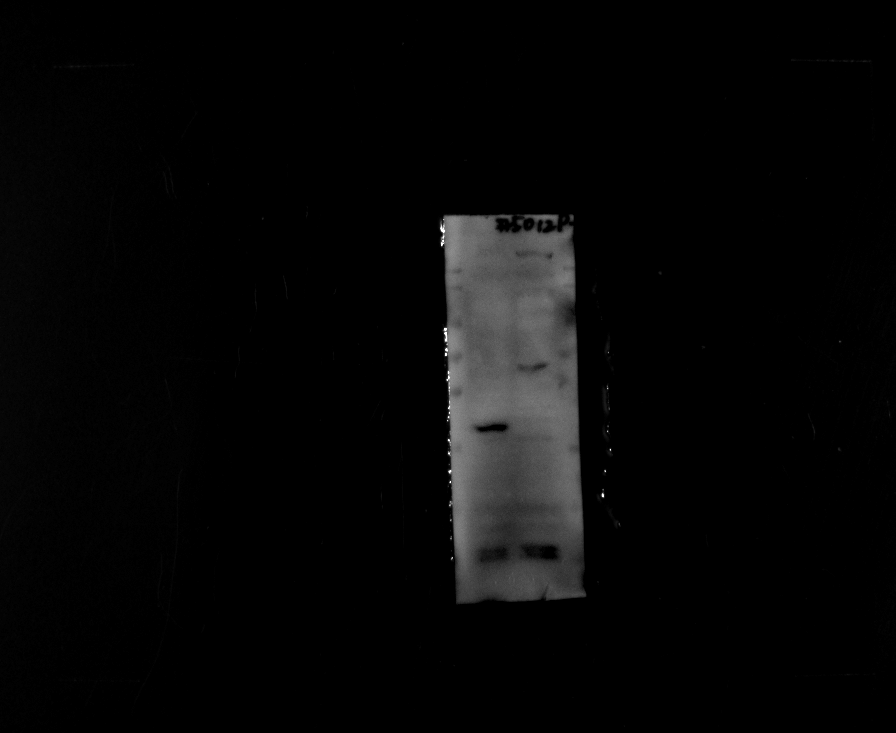

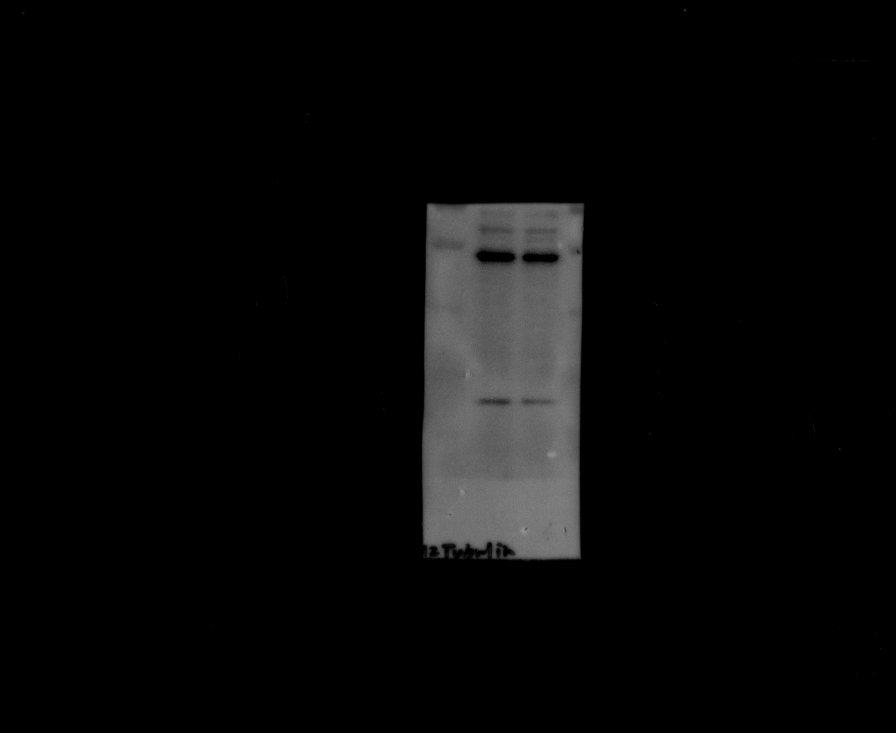


**GAPDH**

**TM7SF2**

**Figure 1C**

**
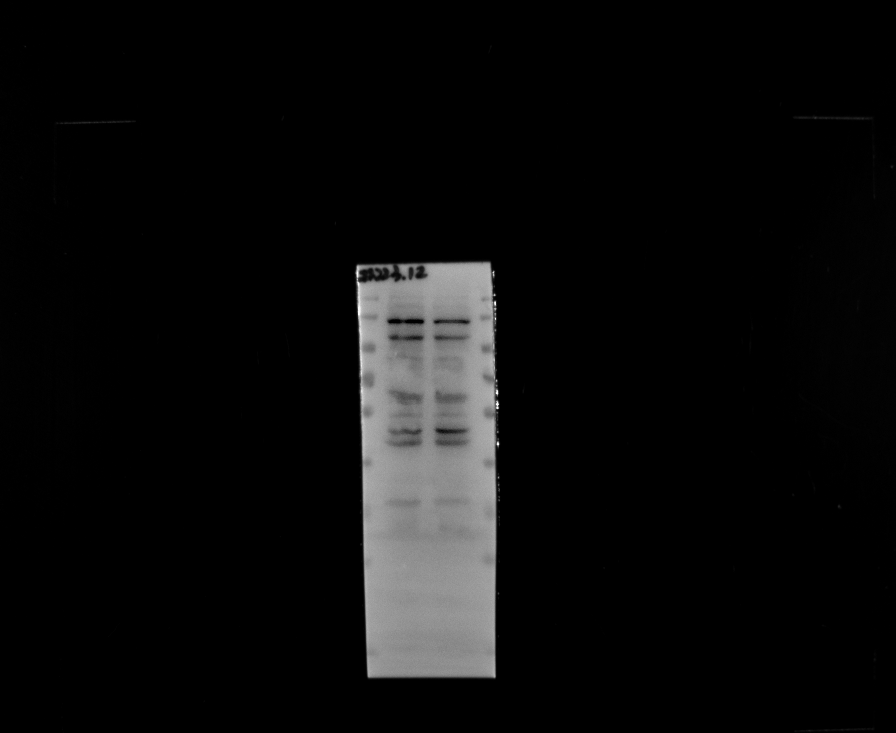

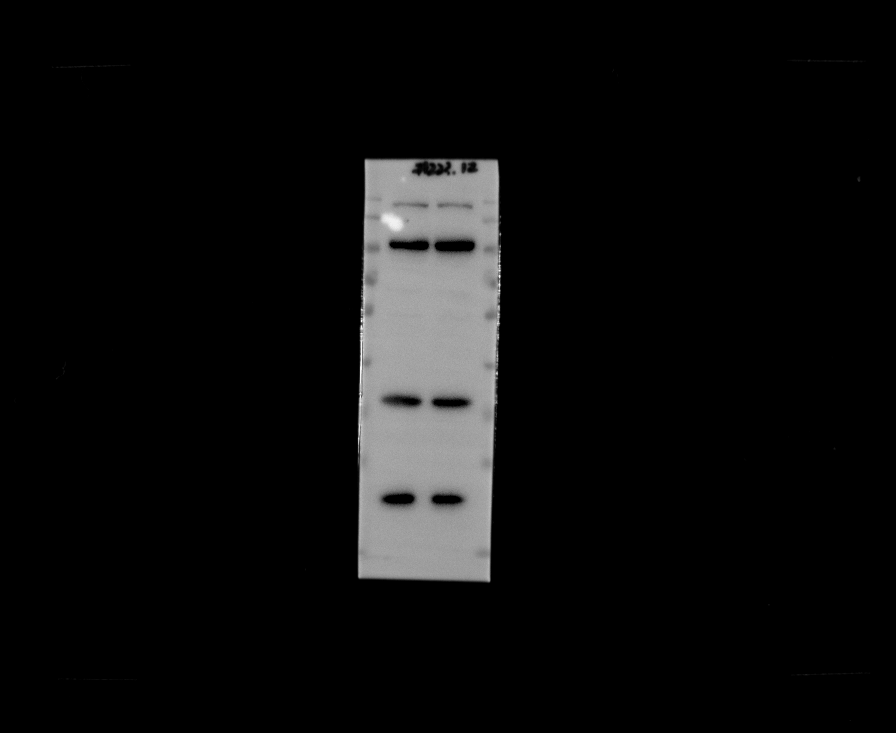
**

**TM7SF2**

**GAPDH**

**Figure 1D**

**
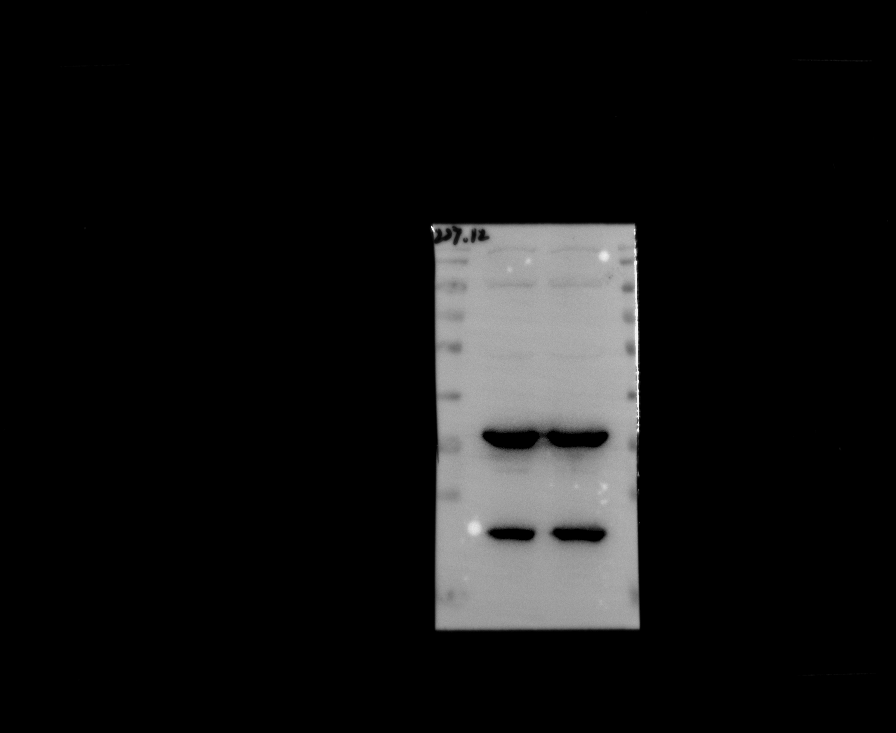

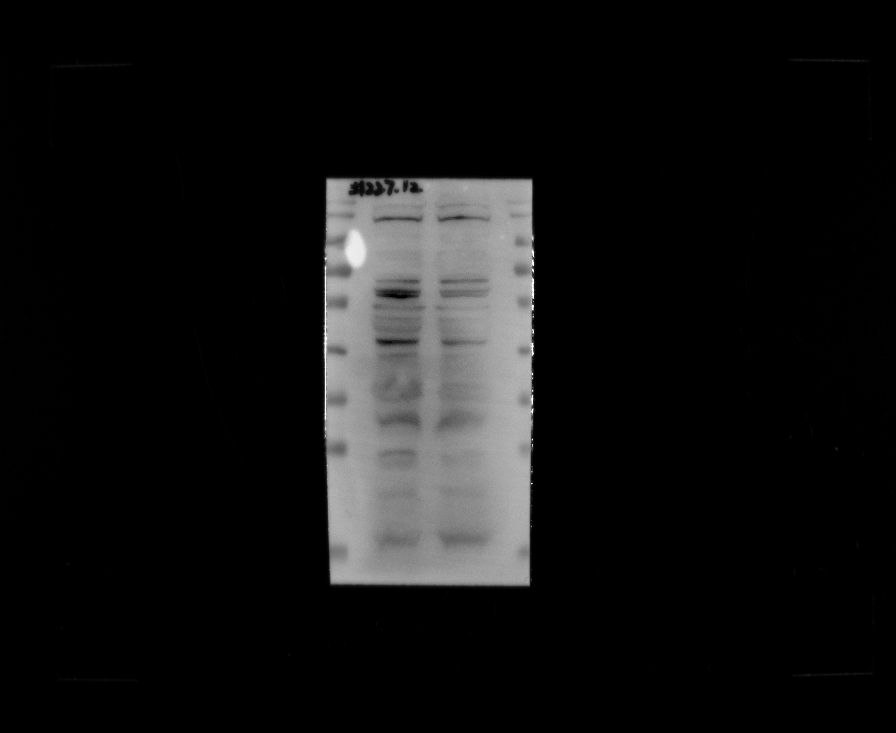
**

**TM7SF2**

**GAPDH**

**Figure 2B**


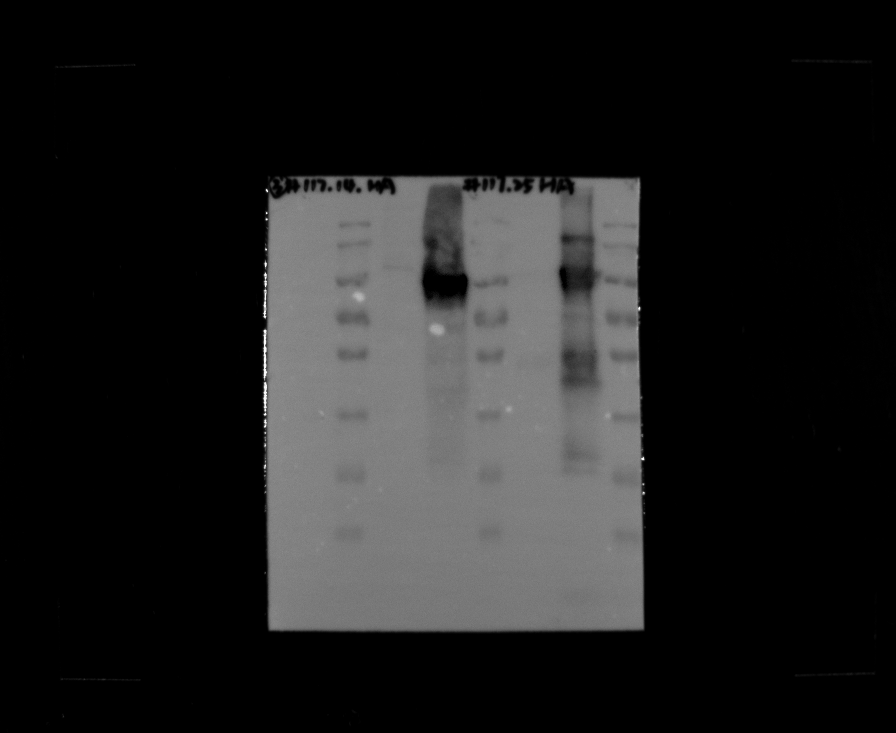

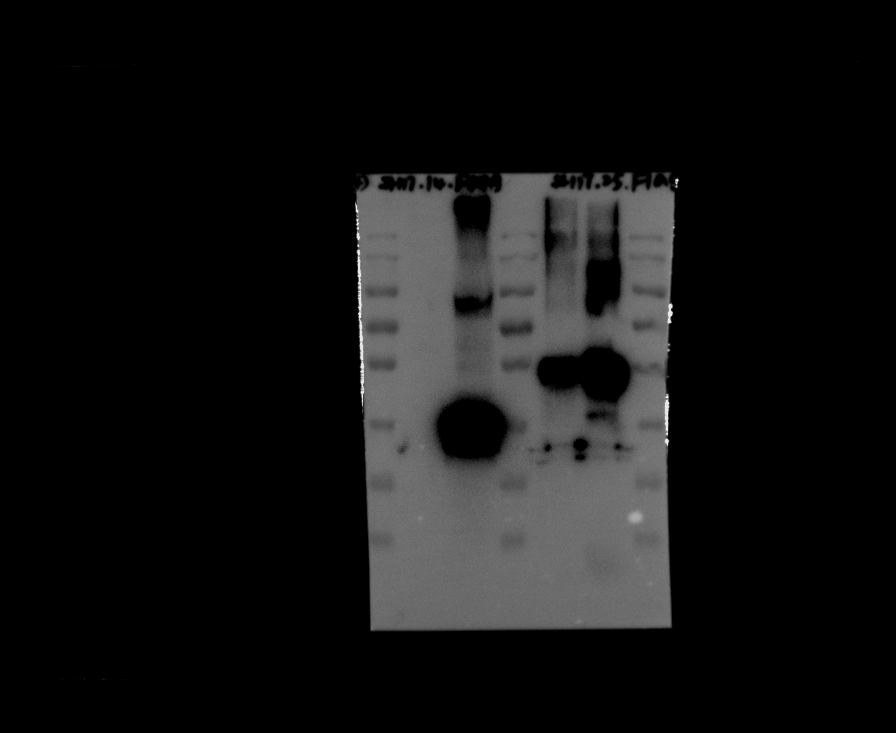

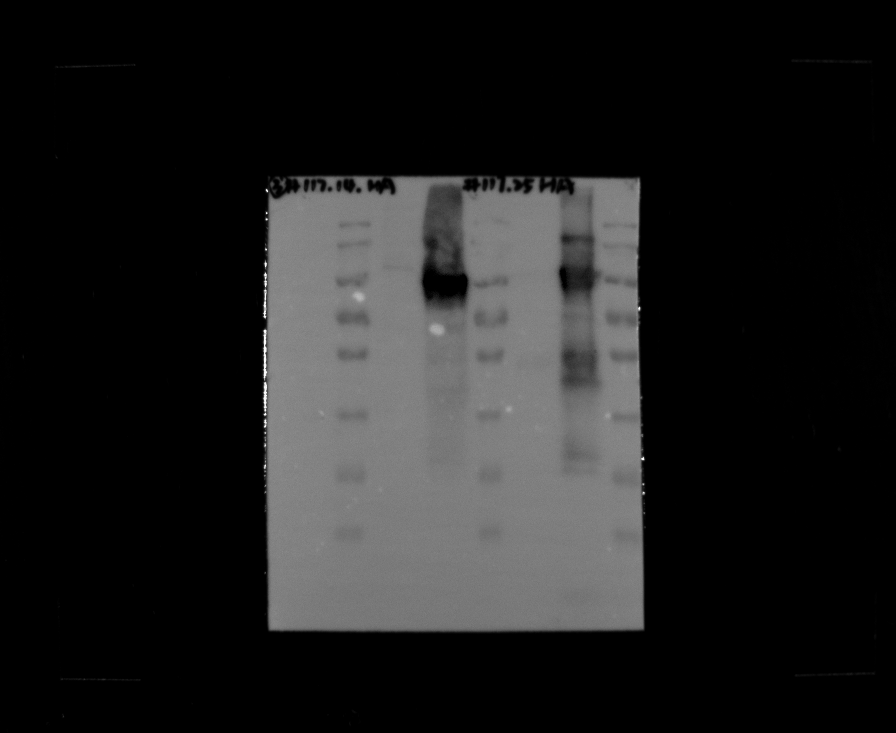

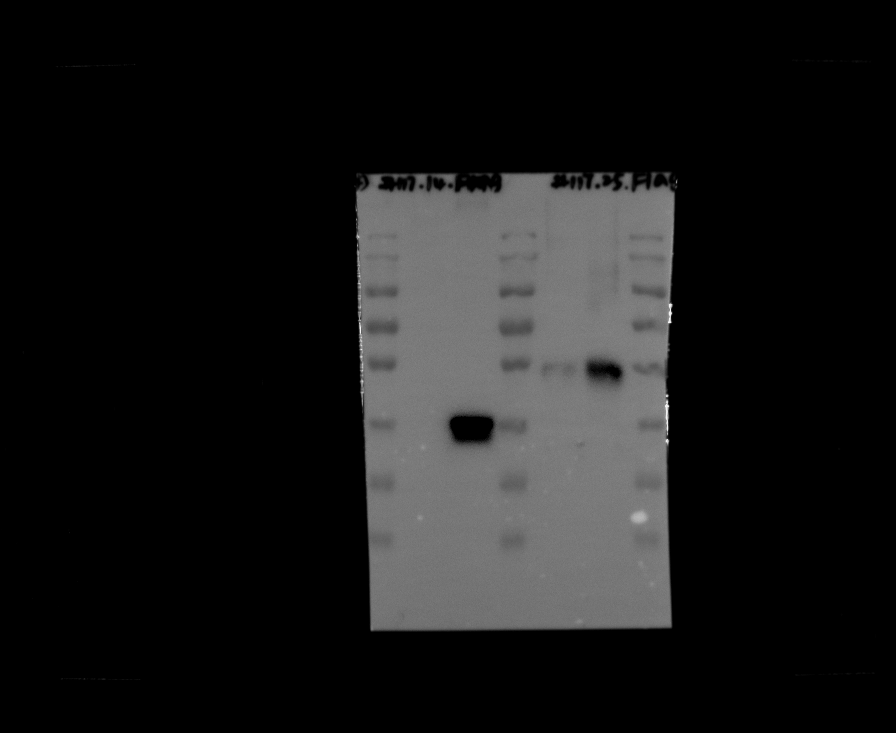


**TM7SF2**

**TM7SF2**

**CPT1A**

**CPT1A**


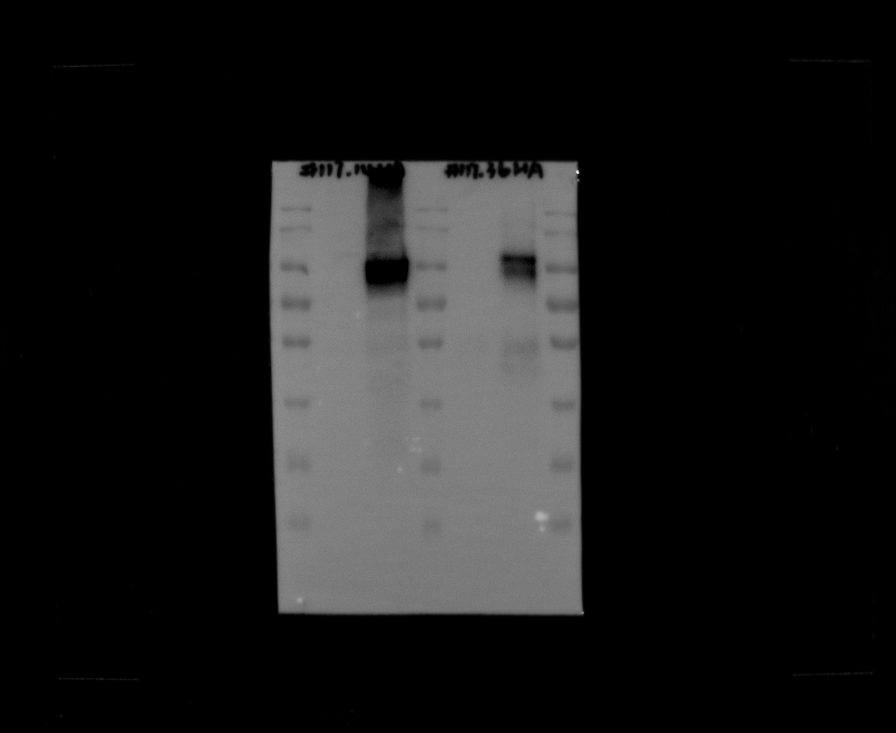

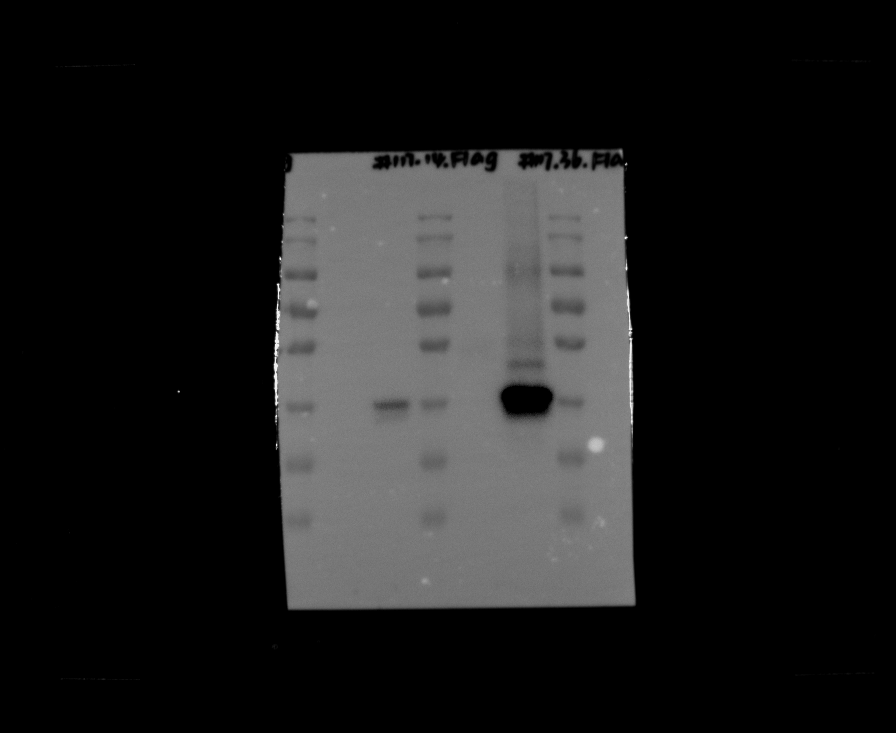

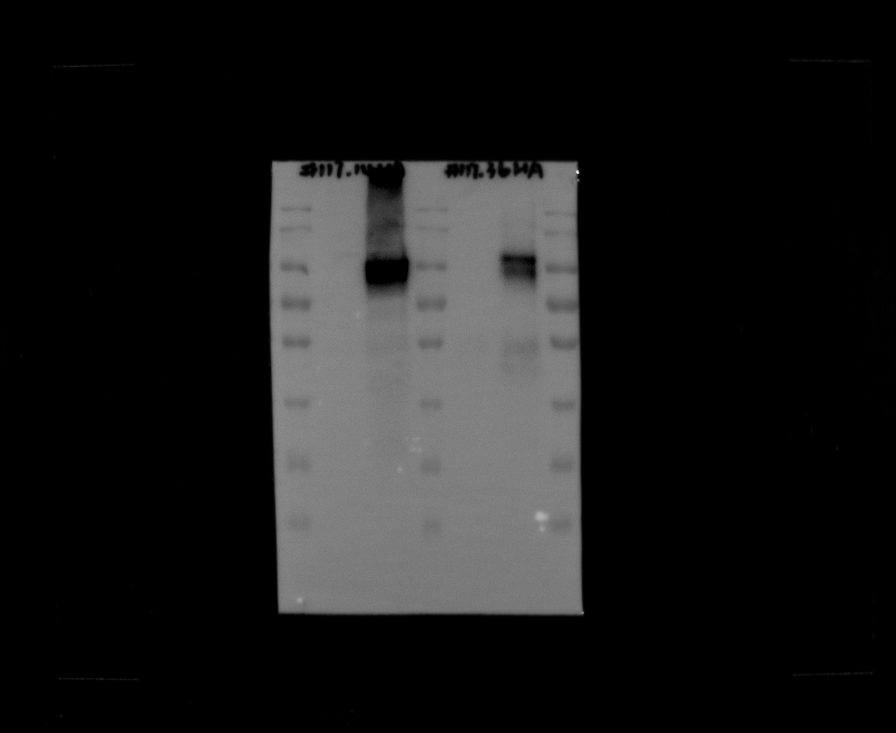

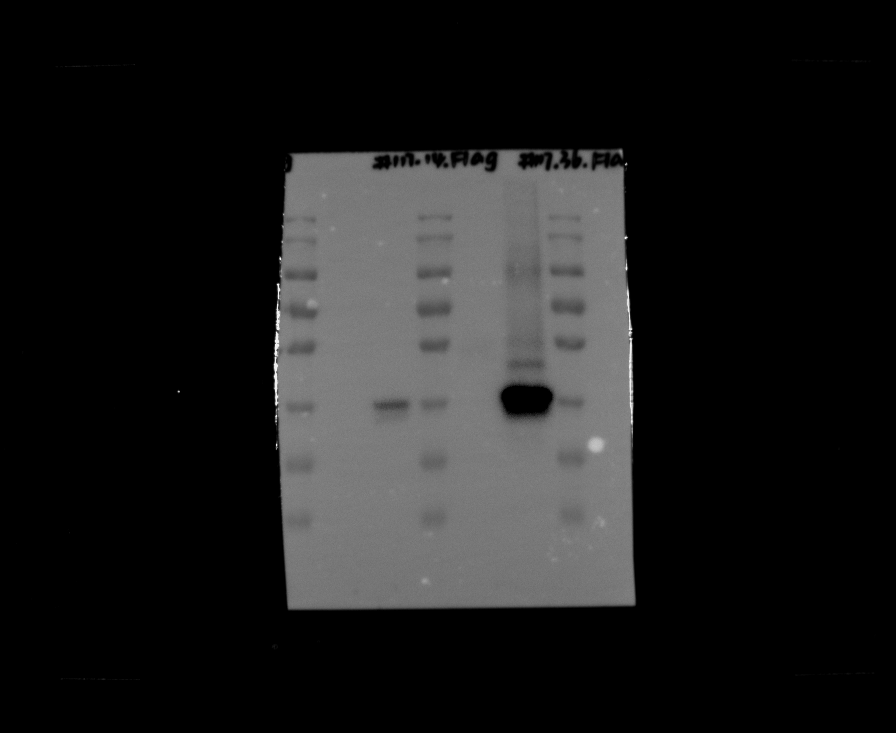


**TM7SF2**

**TM7SF2**

**CPT1A**

**CPT1A**

**Figure 2B**


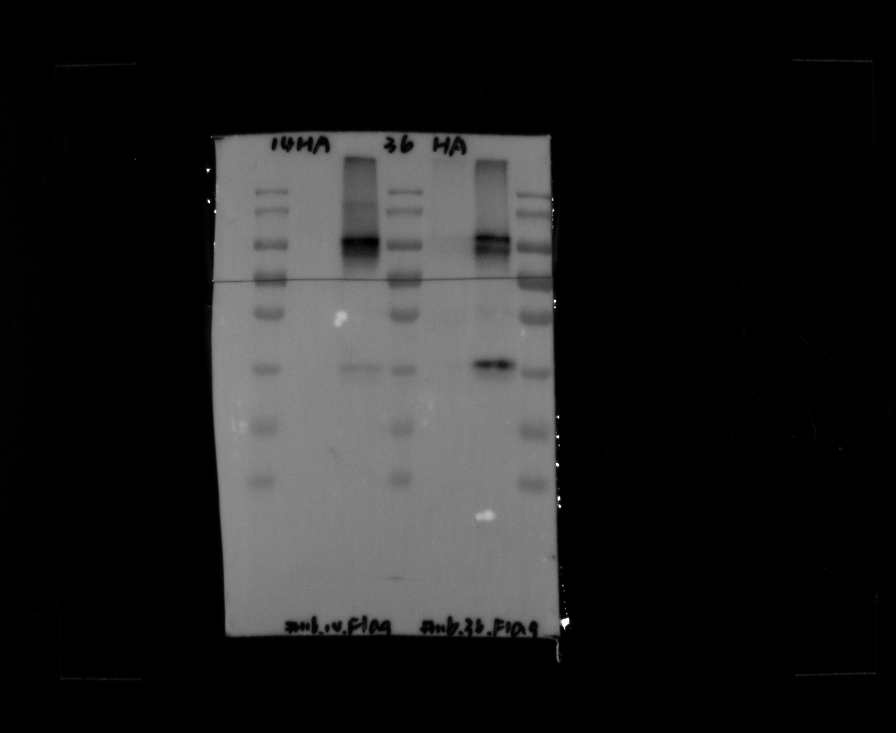

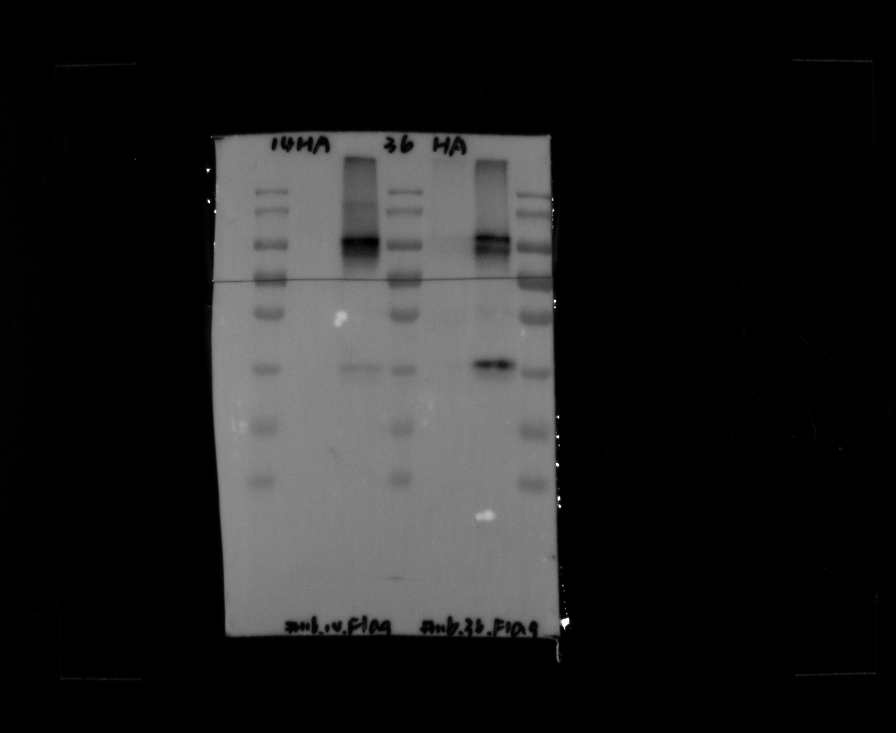


**CPT1A**

**CPT1A**

**TM7SF2**

**TM7SF2**


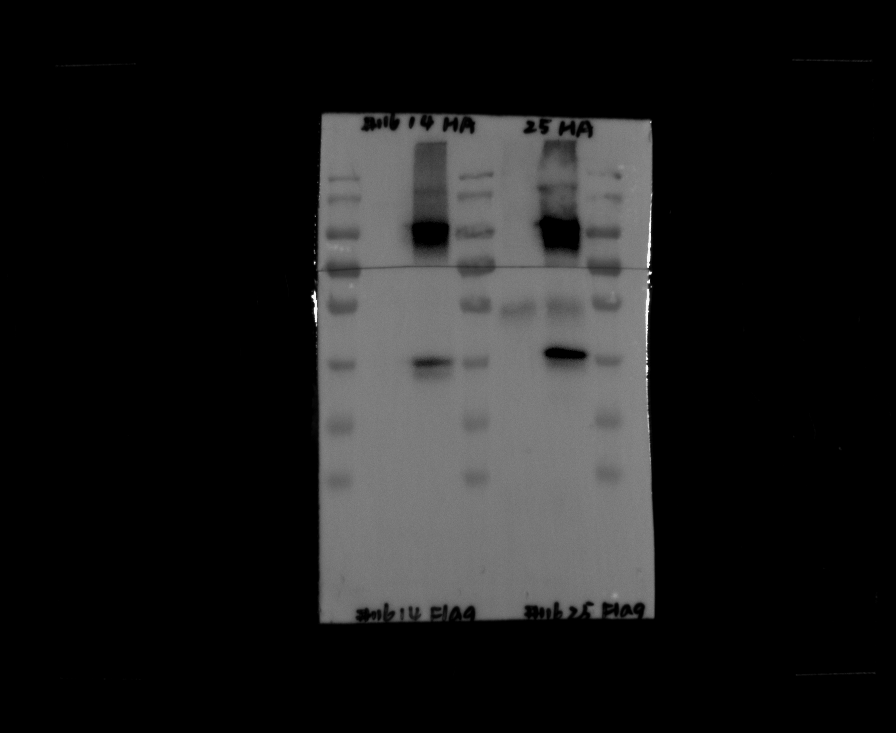

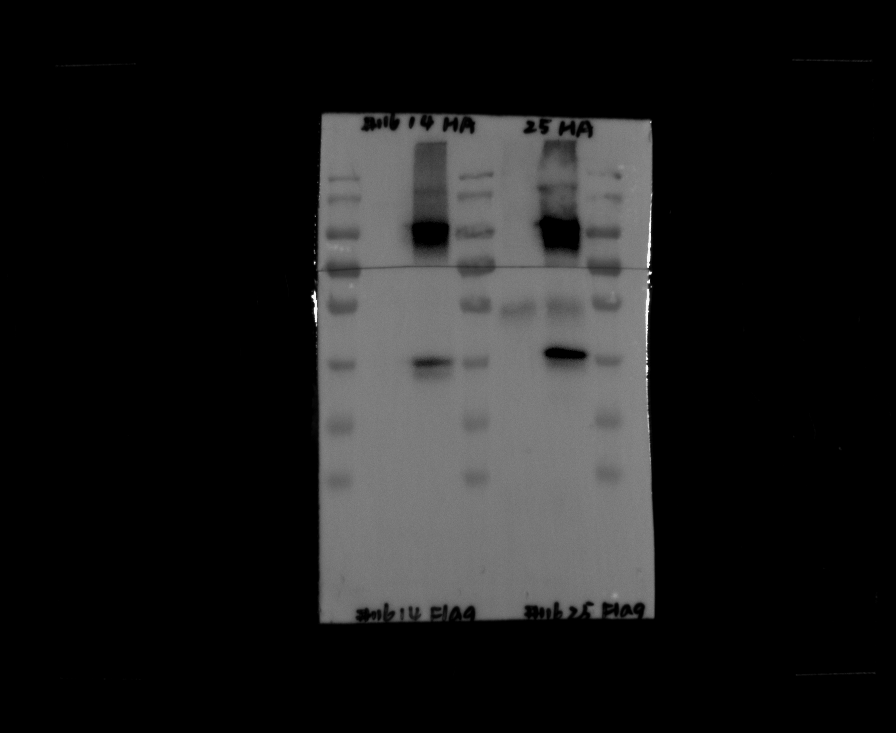


**CPT1A**

**CPT1A**

**TM7SF2**

**TM7SF2**

**Figure 2C**


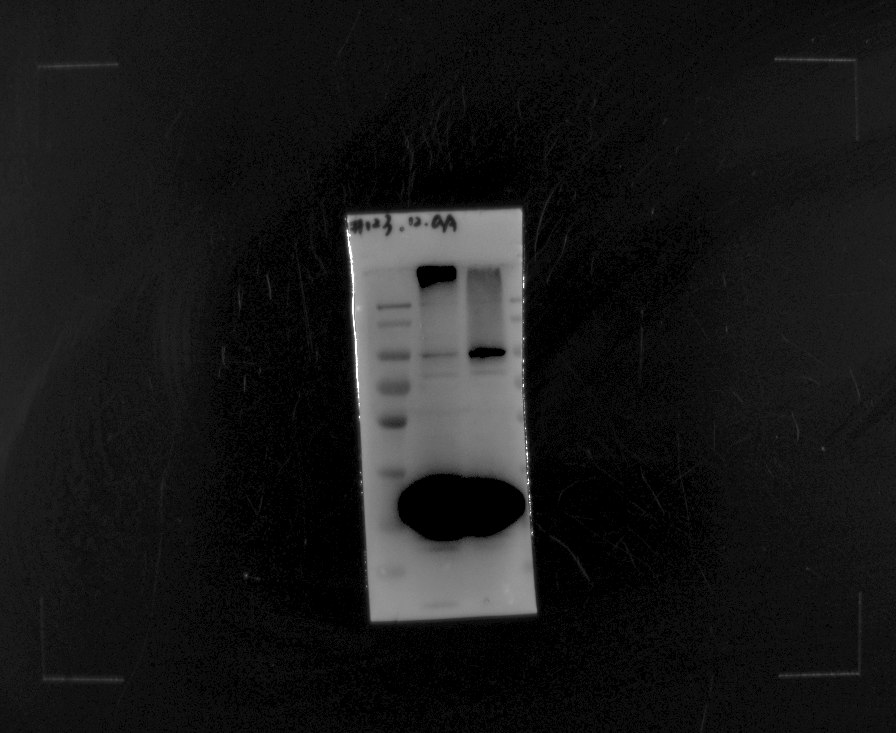

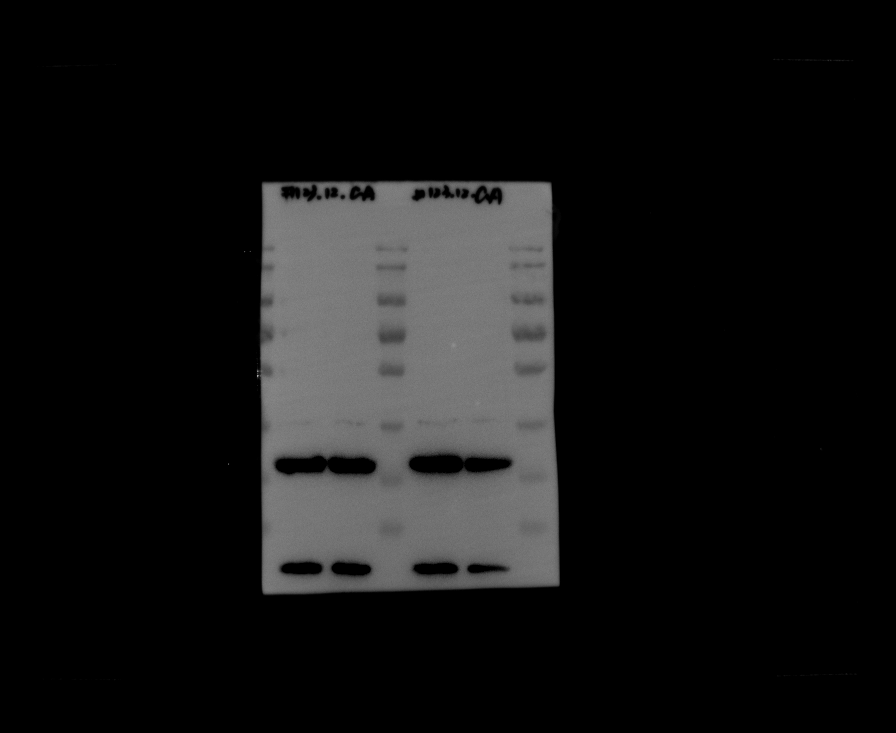


**GAPDH**

**CPT1A**

**Figure 2D**


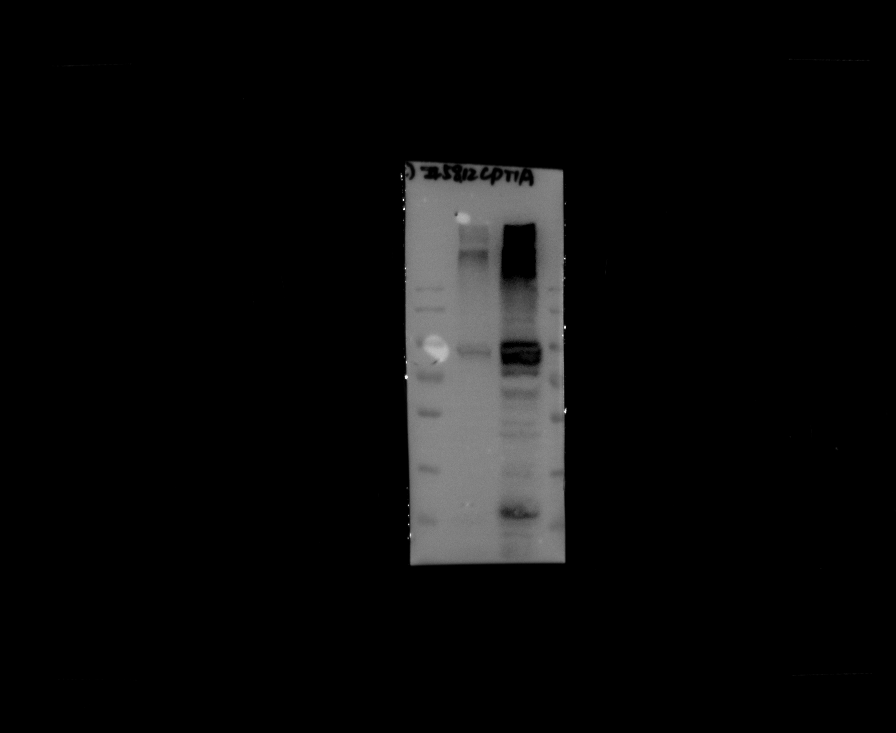

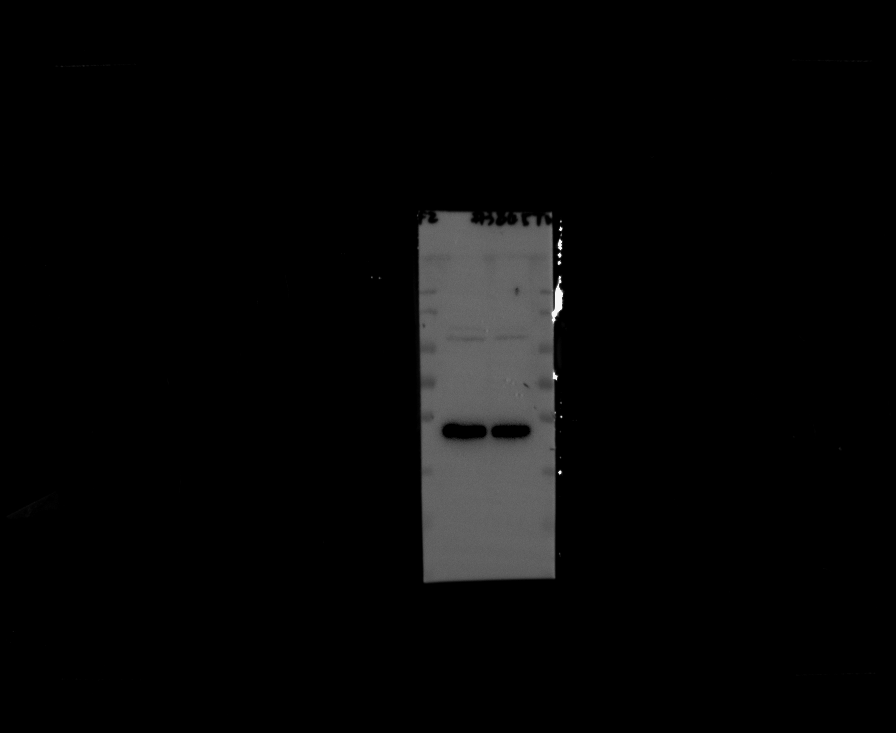


**TUBULIN**

**CPT1A**

**Figure 2E**


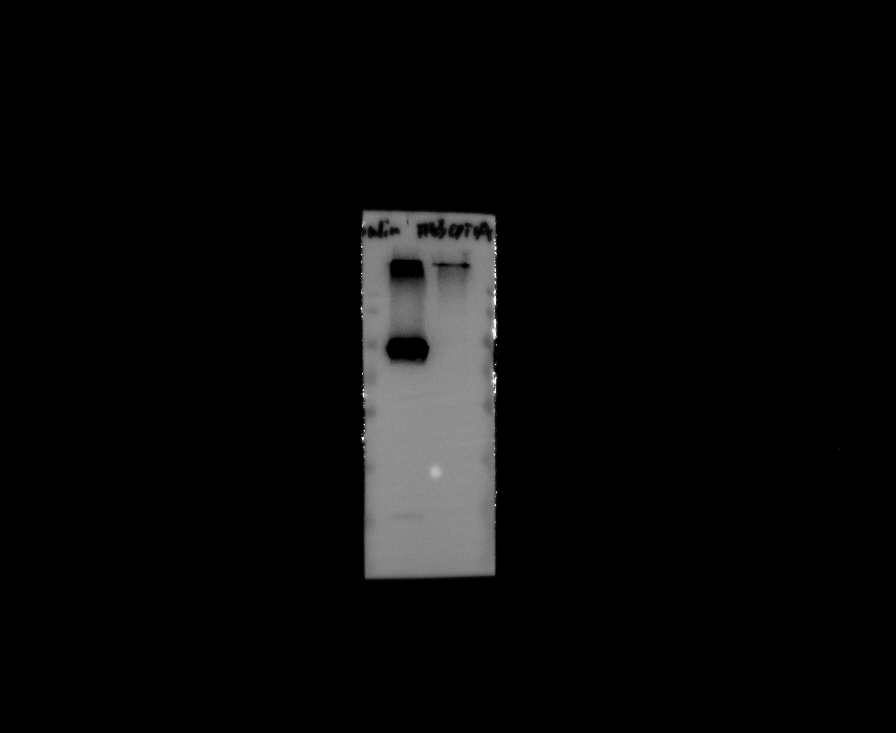

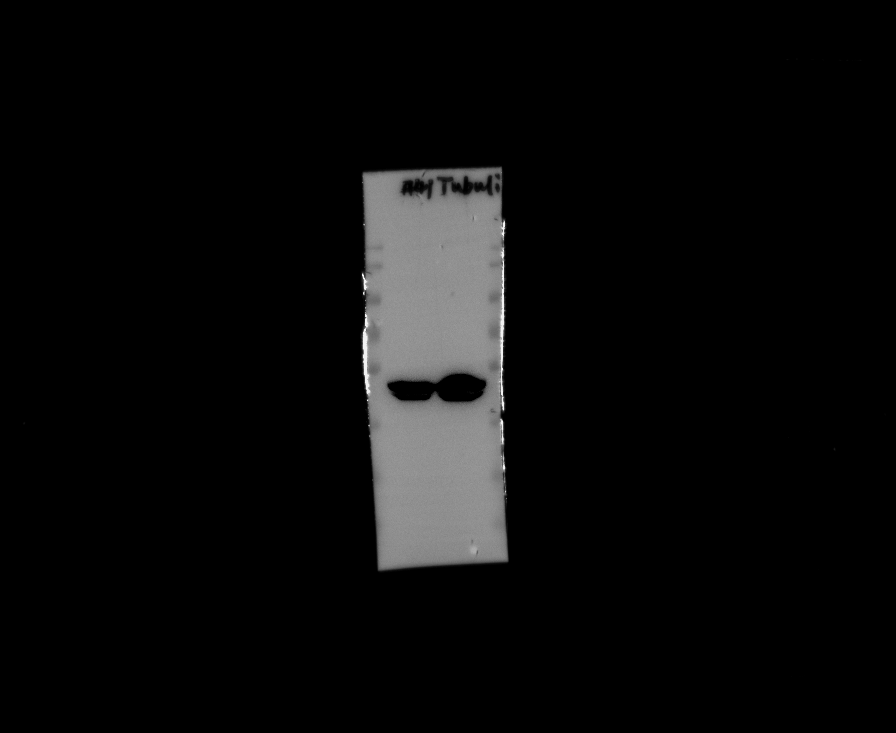


**GAPDH**

**CPT1A**

**Figure 2F**

**
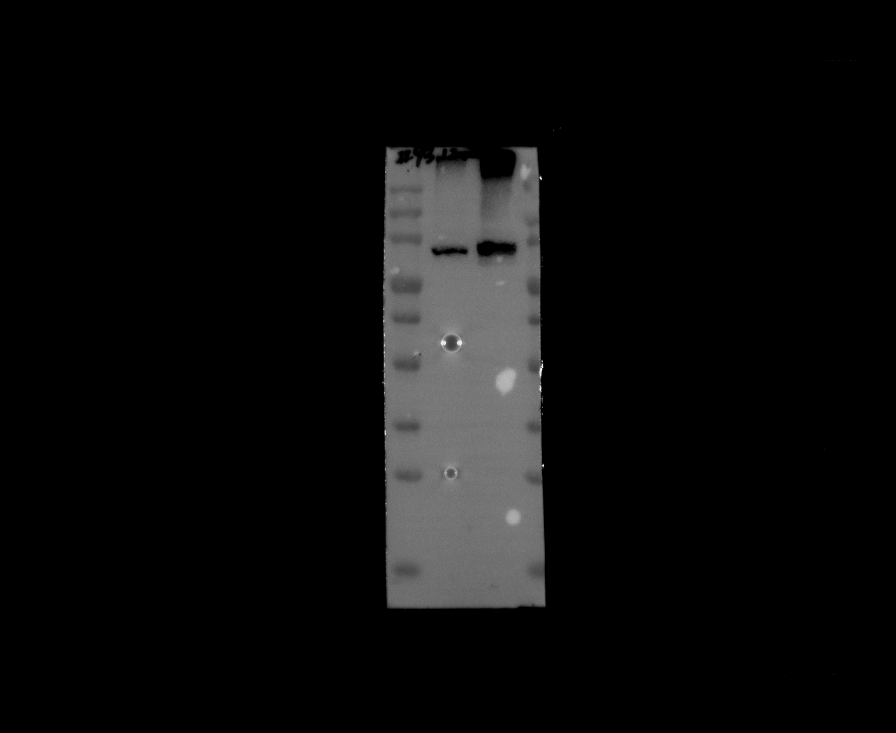

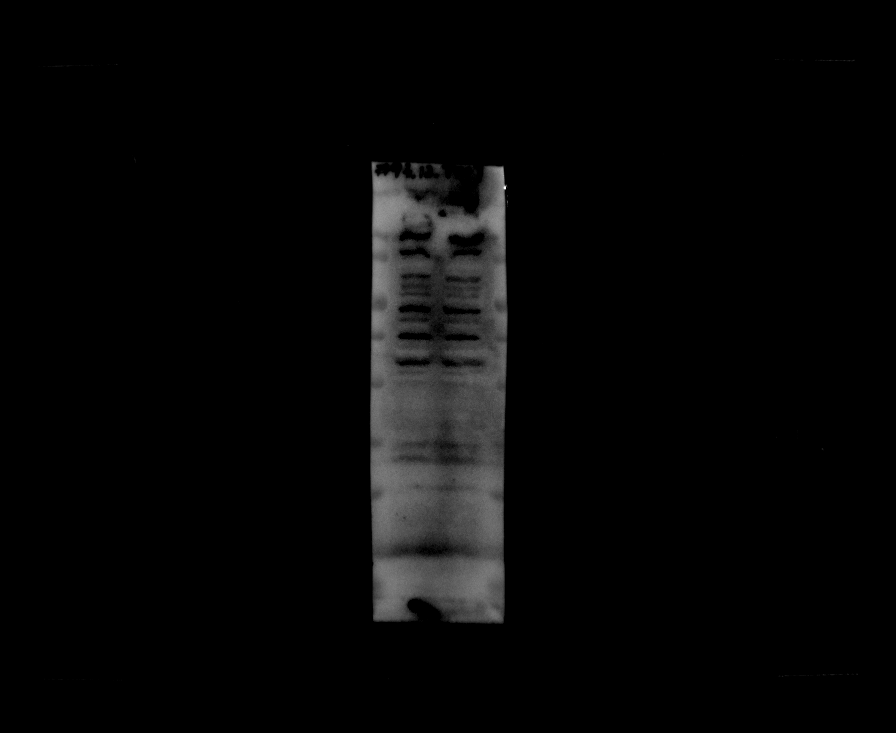

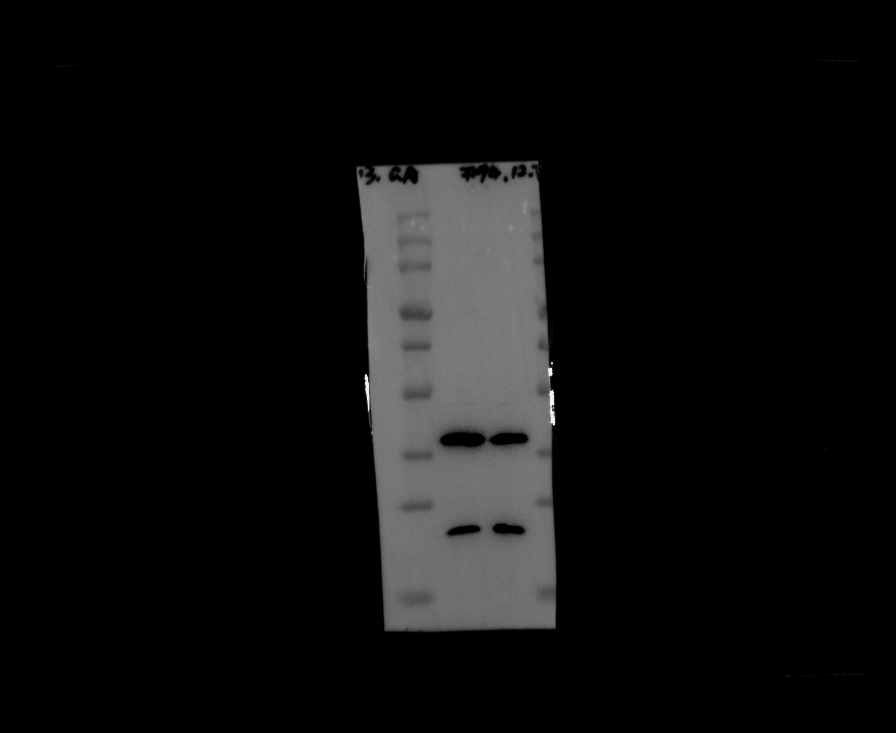
**

**GAPDH**

**TM7SF2**

**CPT1A**

**Figure 2G**

**
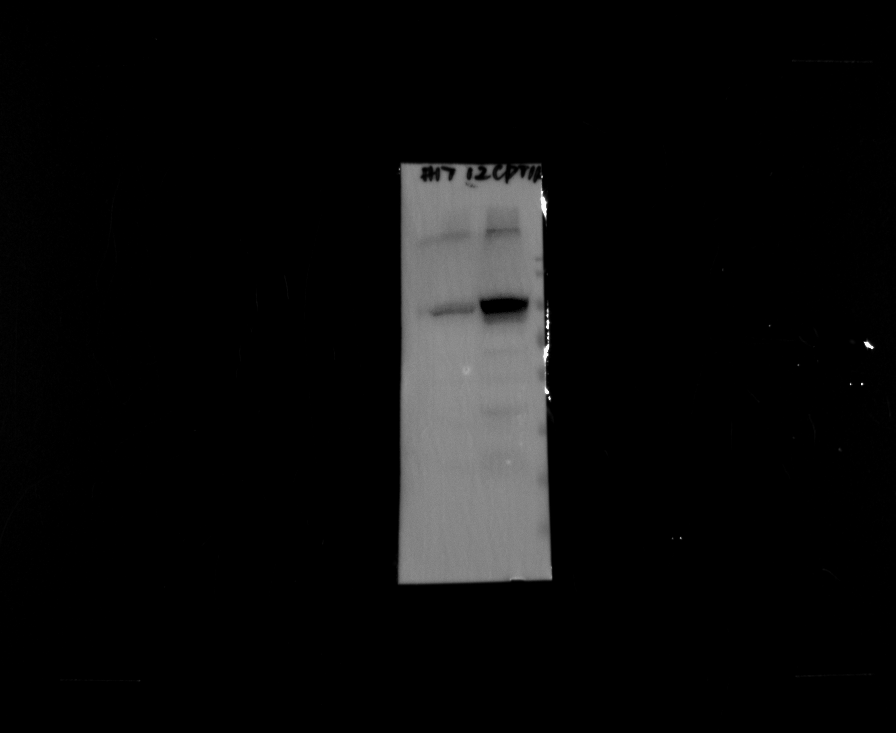

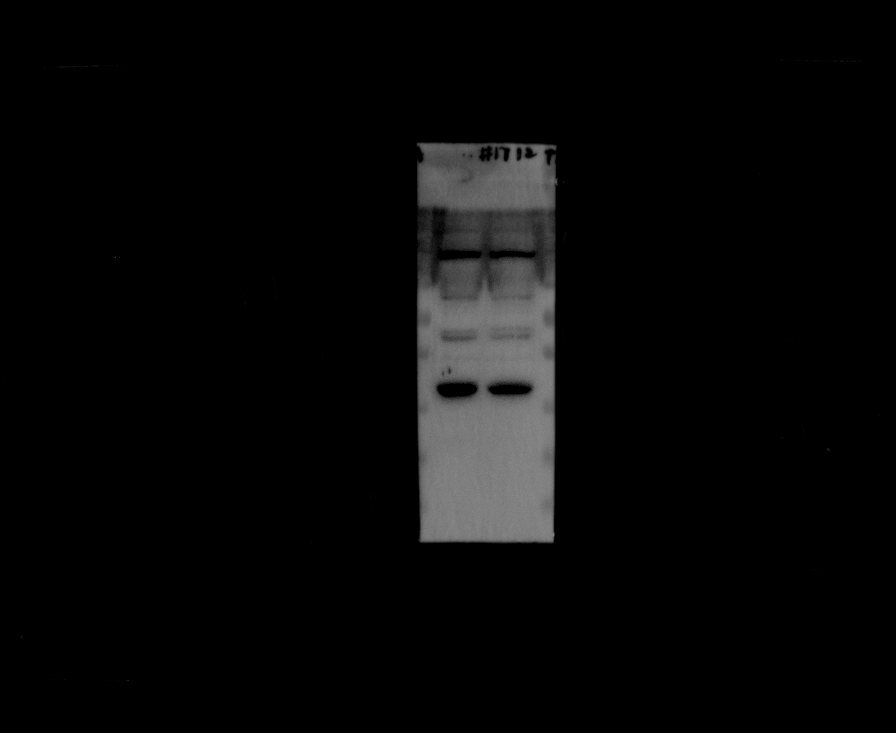

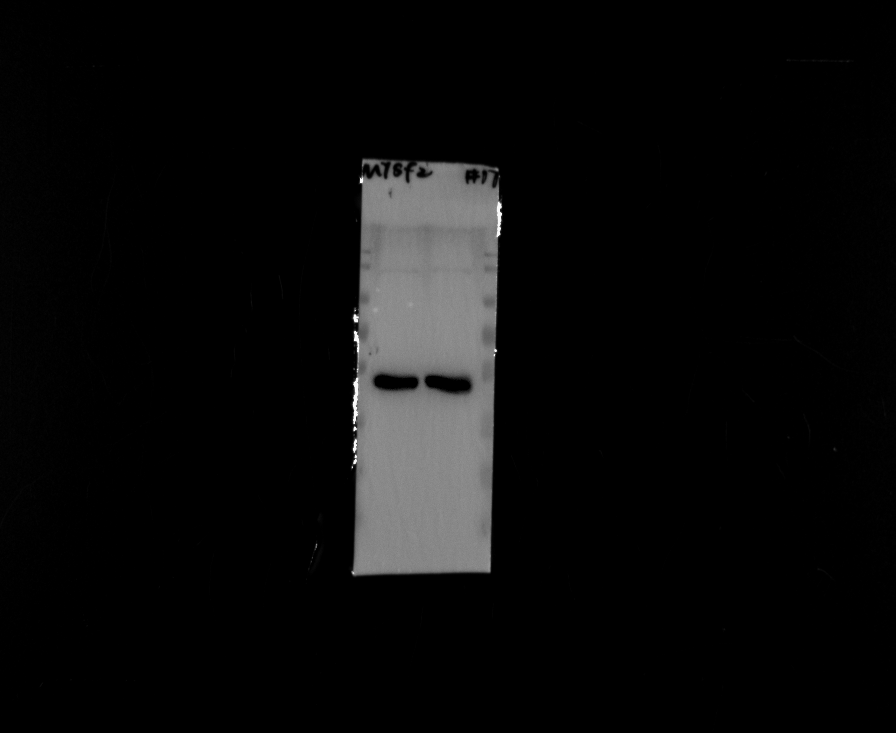
**

**GAPDH**

**CPT1A**

**TM7SF2**

**Figure 4A**

**
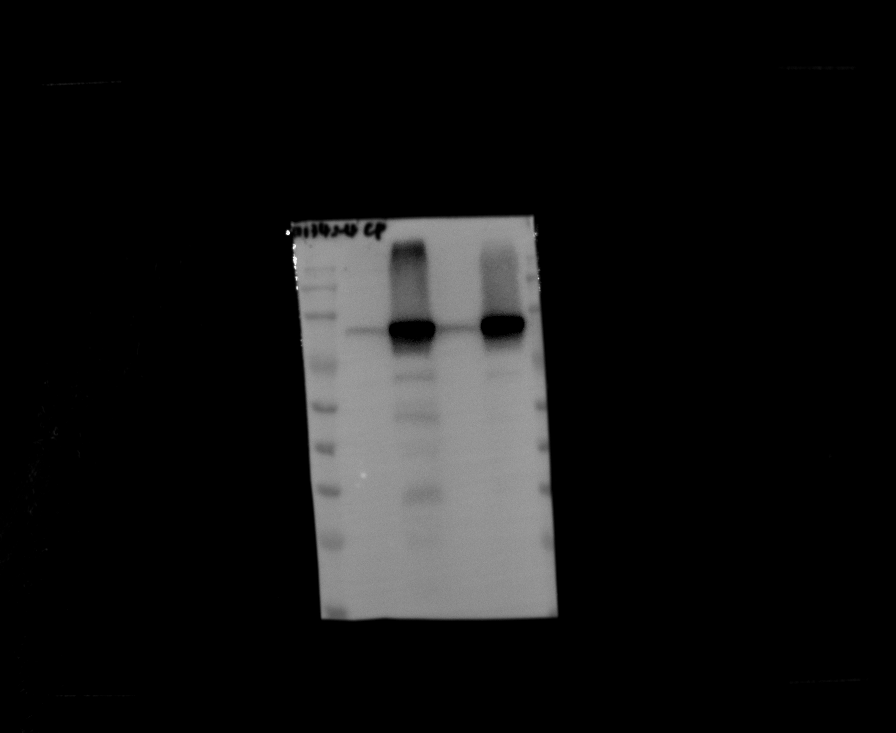

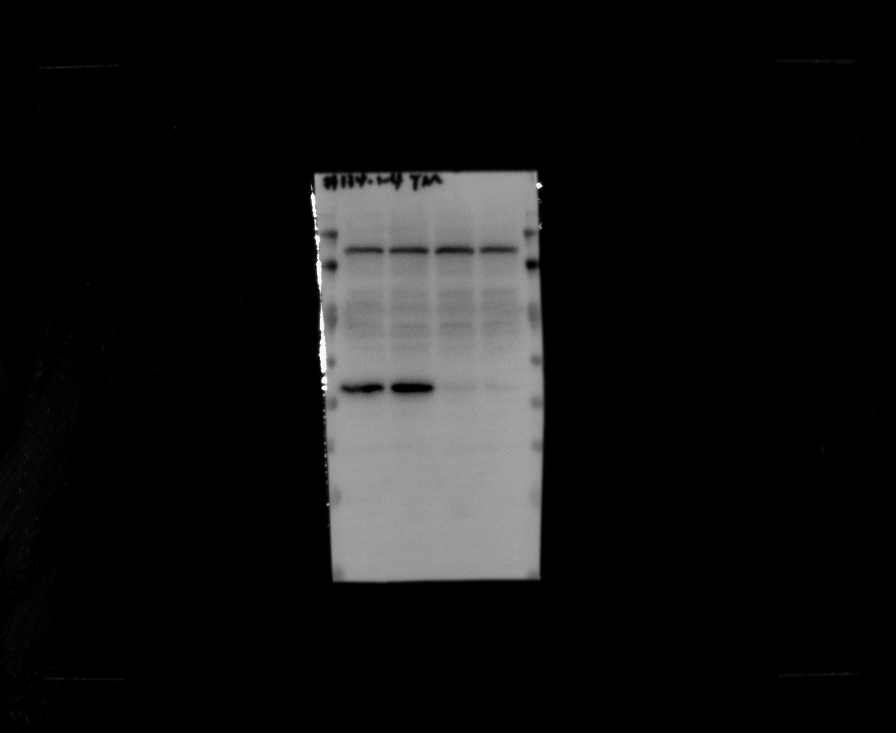

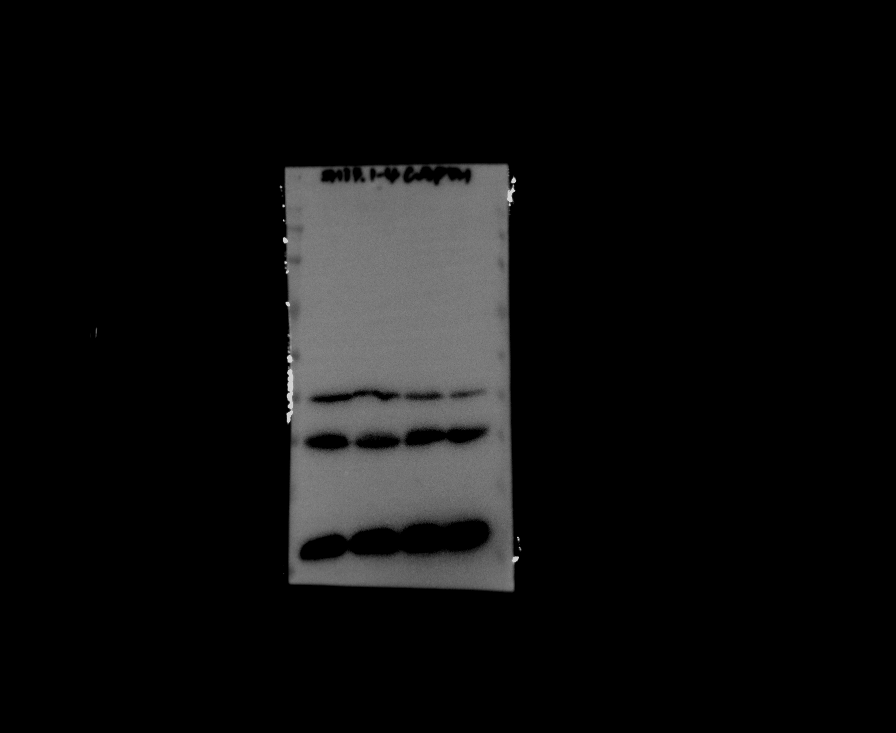
**

**GAPDH**

**TM7SF2**

**CPT1A**

**Figure 5A**

**
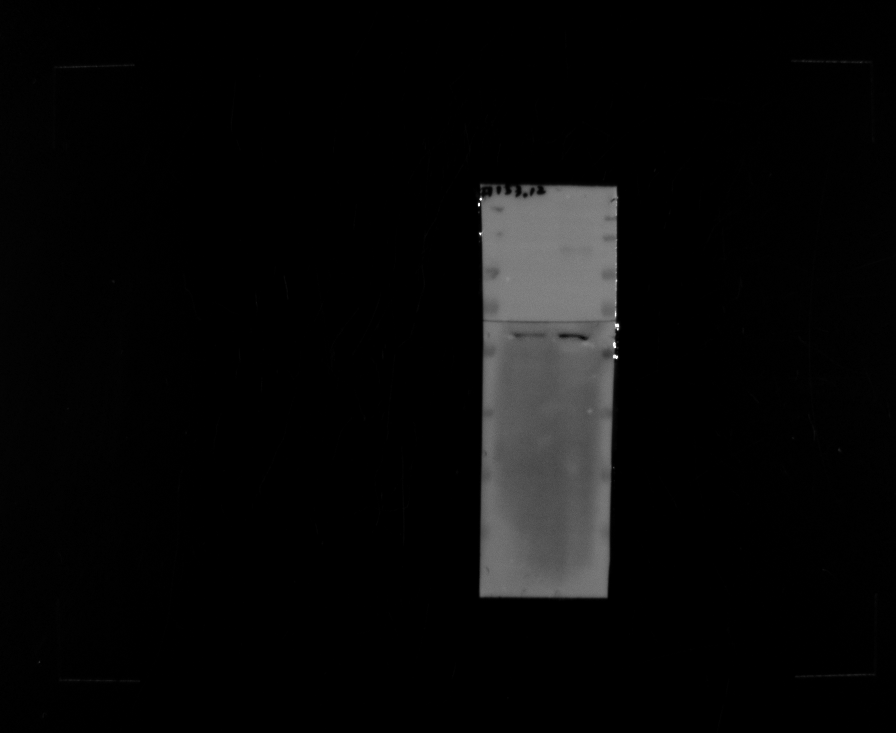

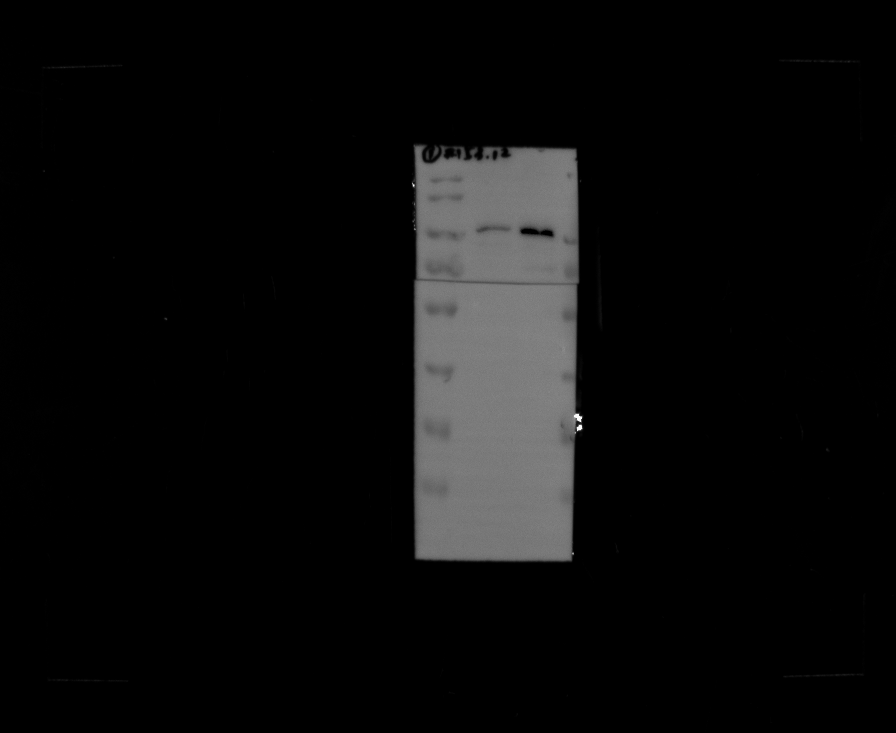

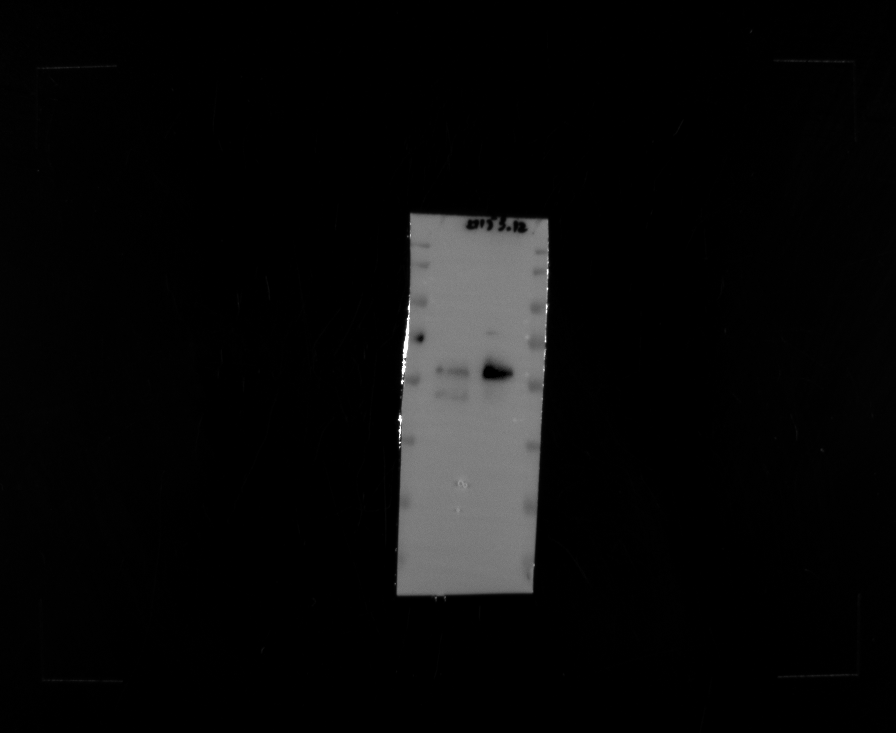
**

**c-Myc**

**β-catenin**

**WNT3A**

**
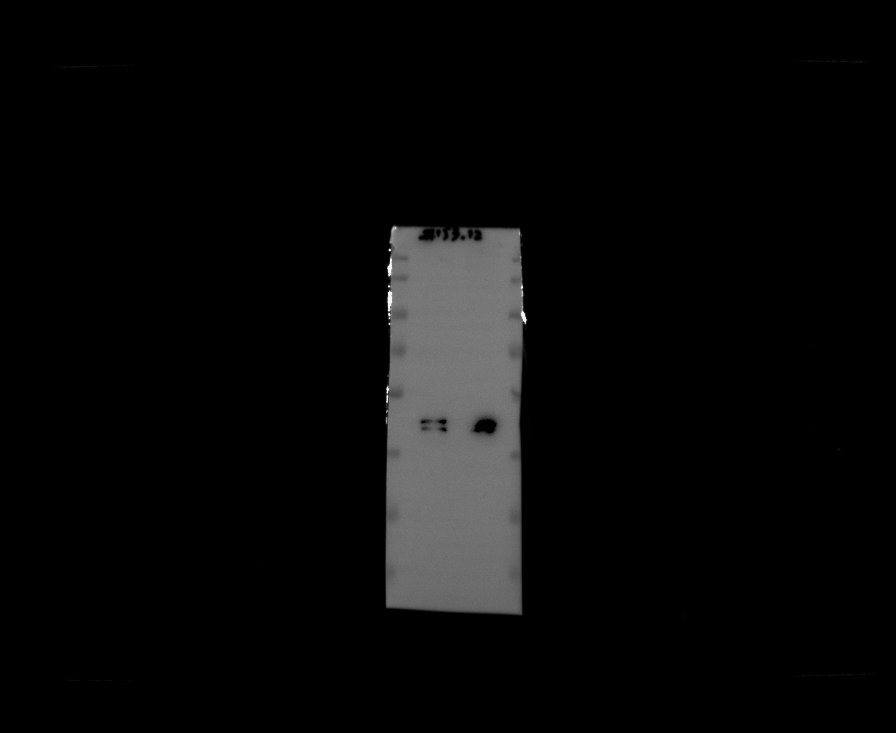

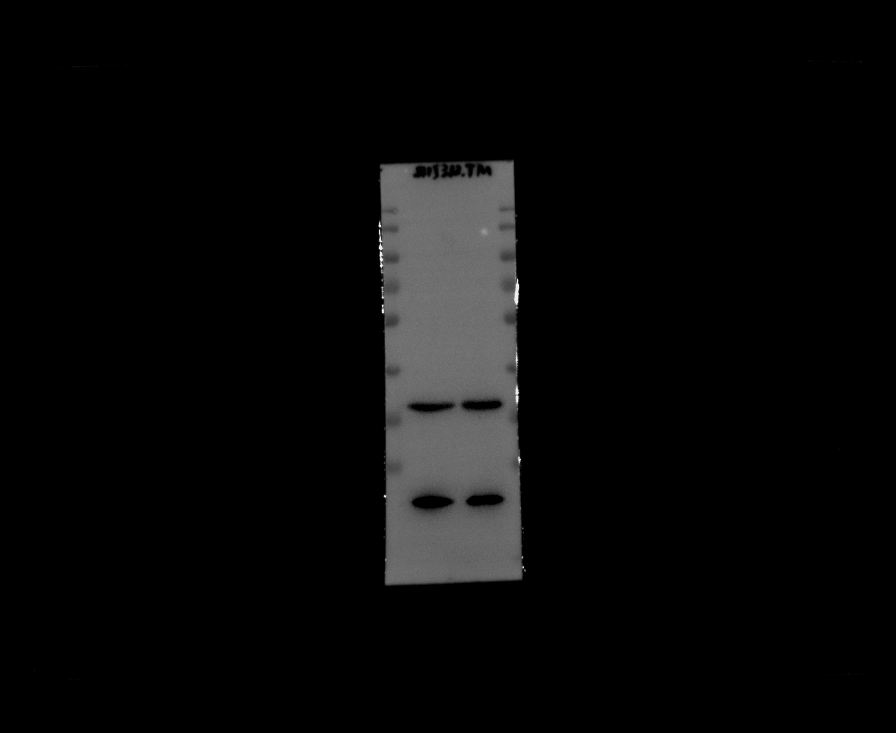
**

**GAPDH**

**TCF1**

**Figure 5B**

**
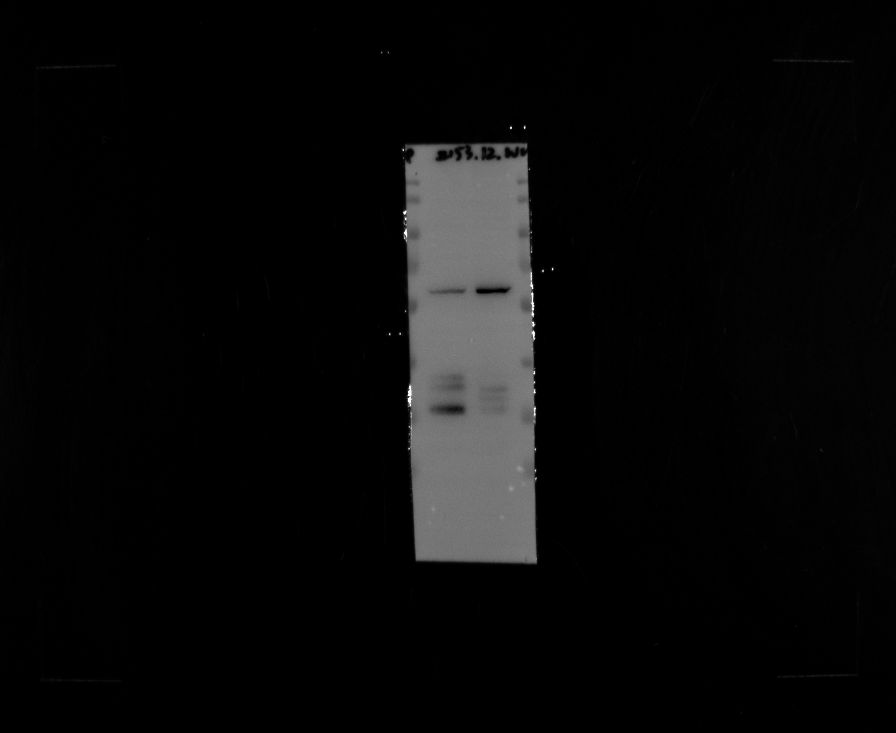

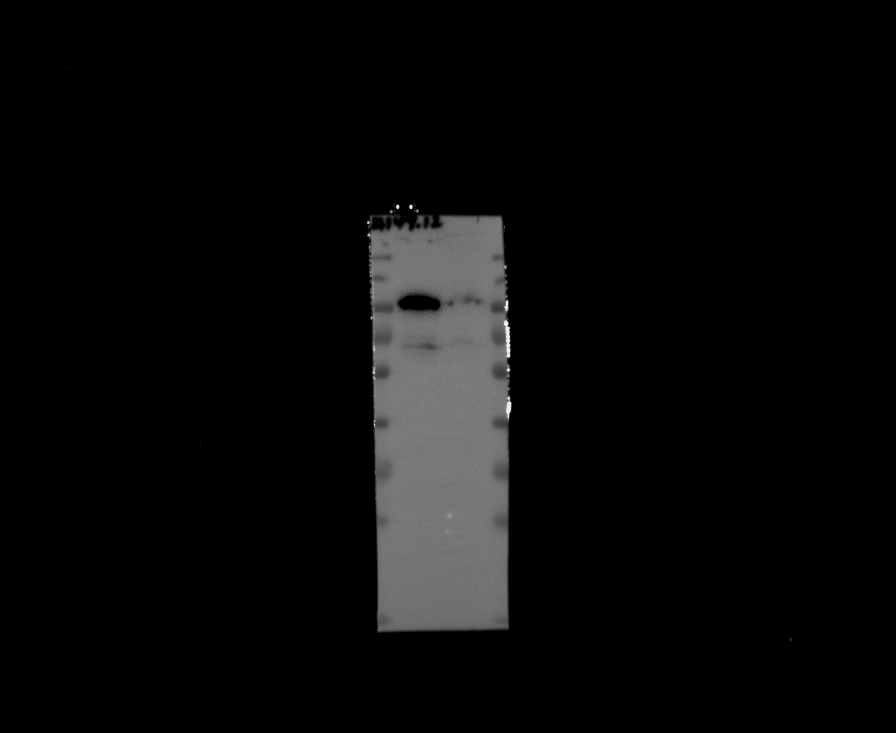

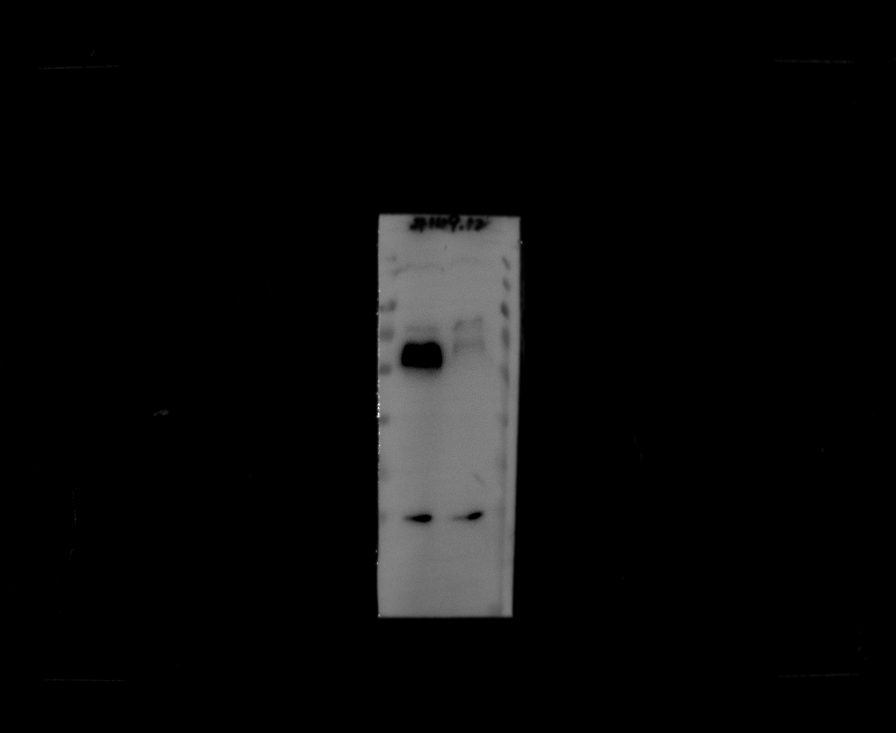
**

**WNT3A**

**c-Myc**

**β-catenin**

**
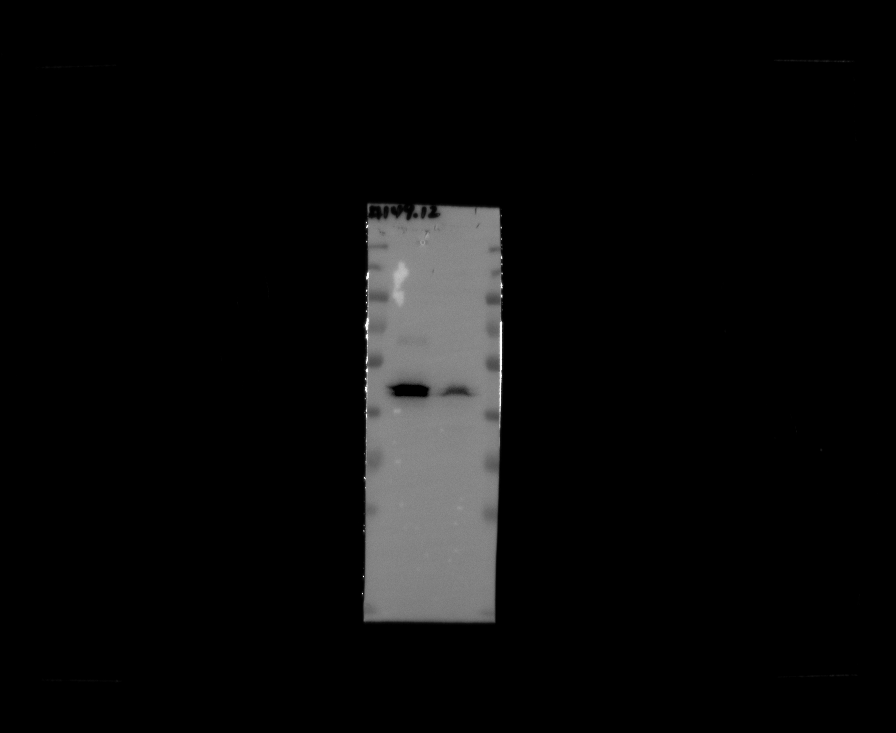

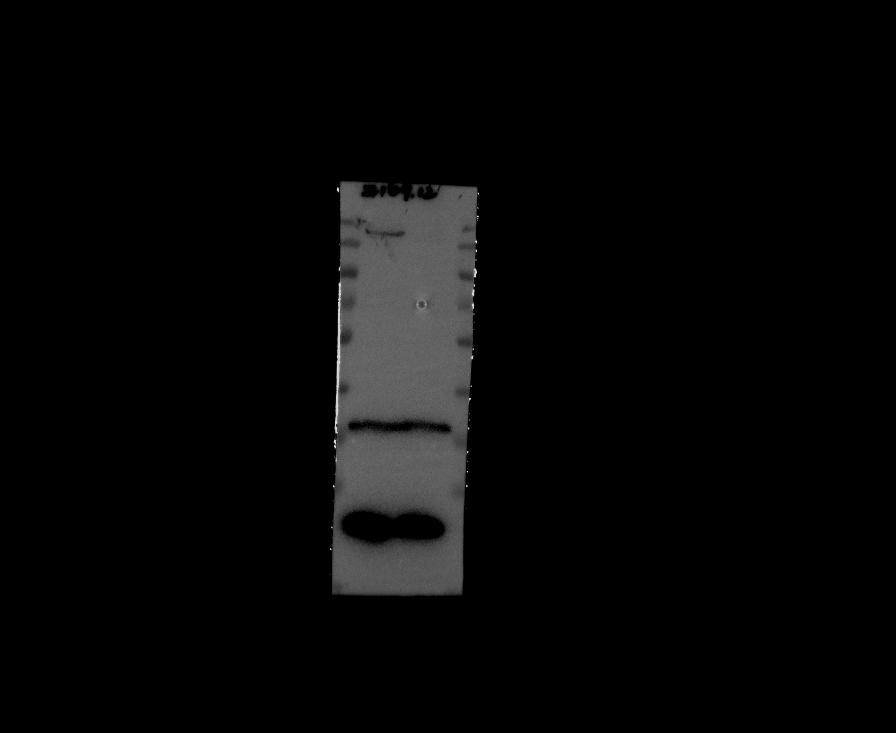
**

**GAPDH**

**TCF1**

**Figure 5C**

**
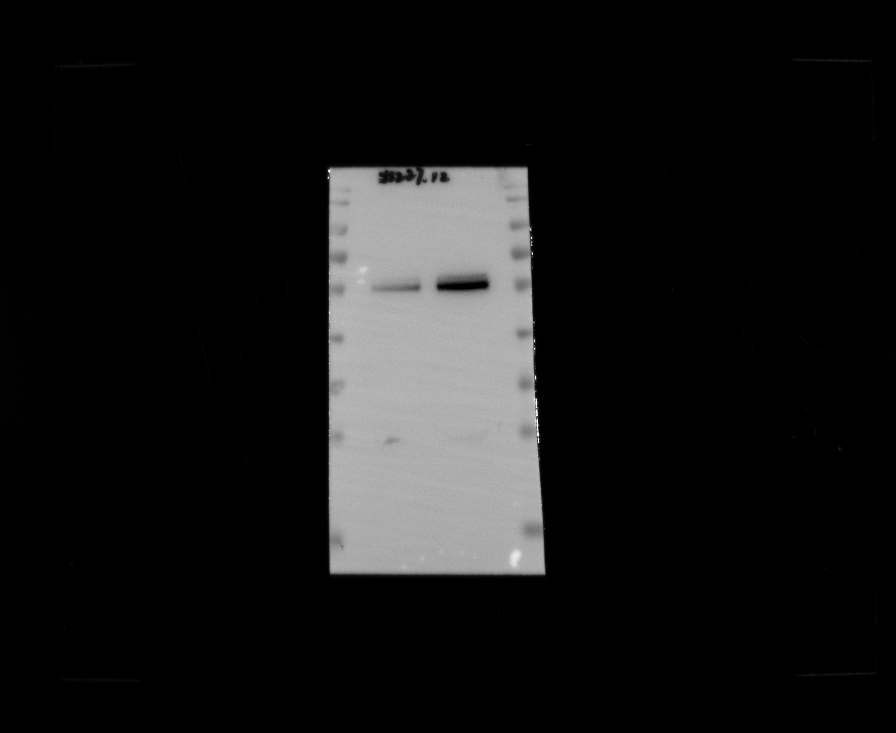

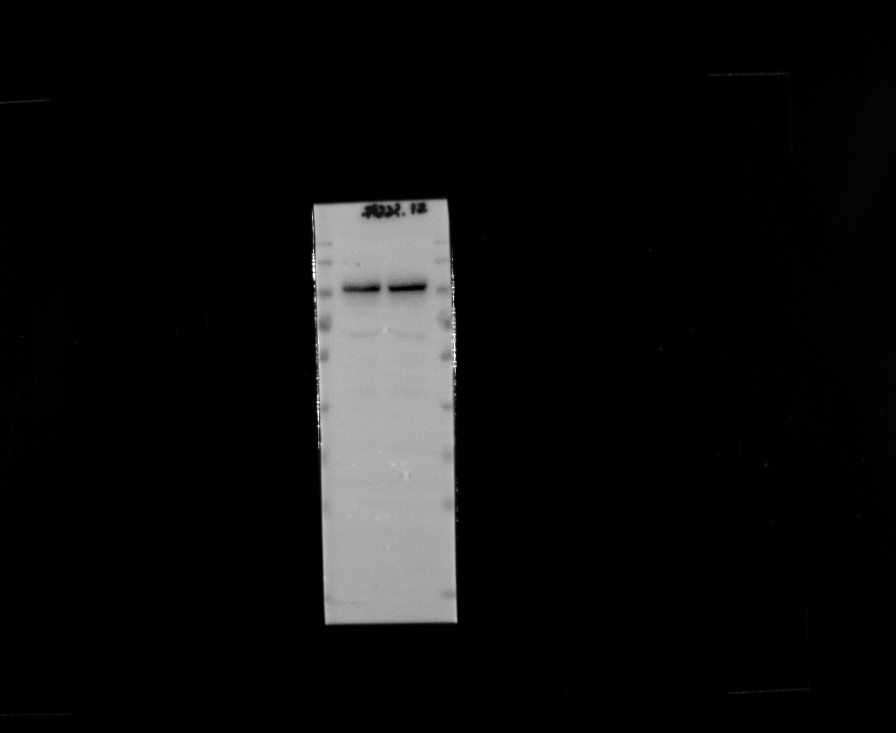

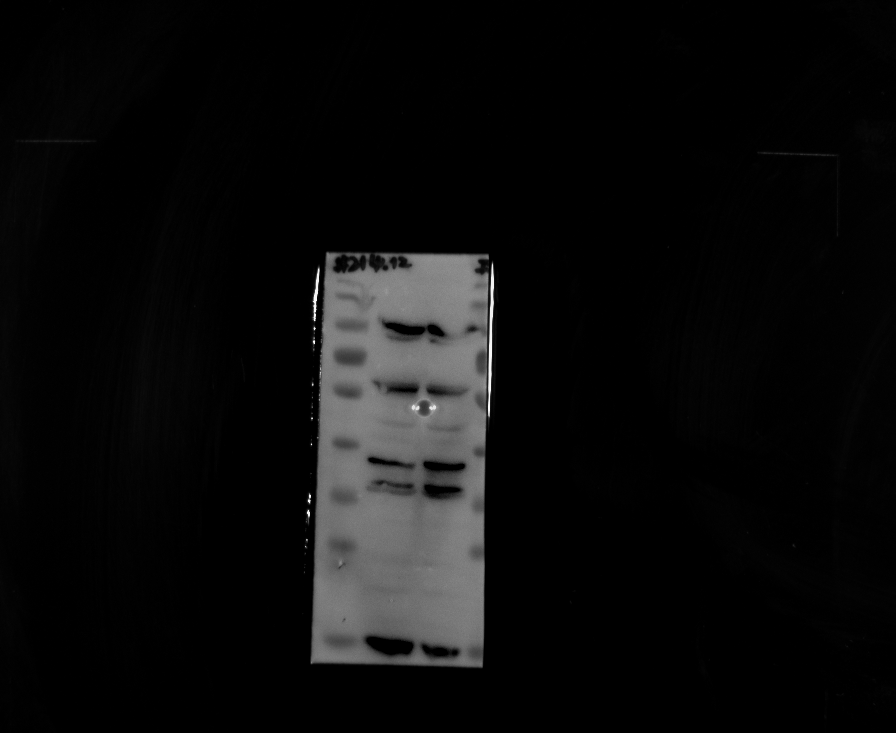
**

**WNT3A**

**β-catenin**

**c-Myc**

**
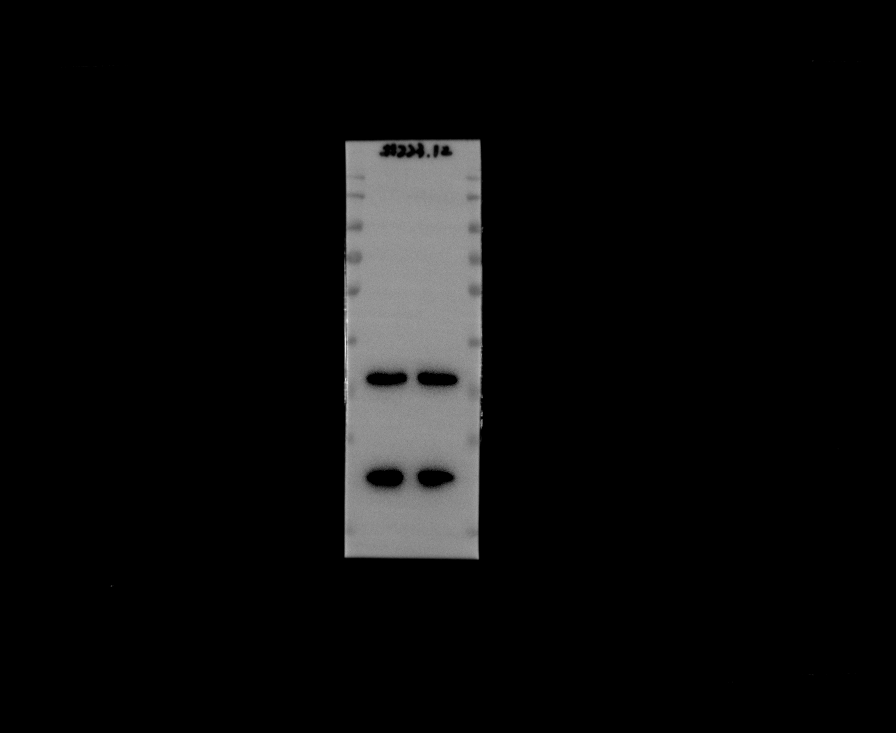

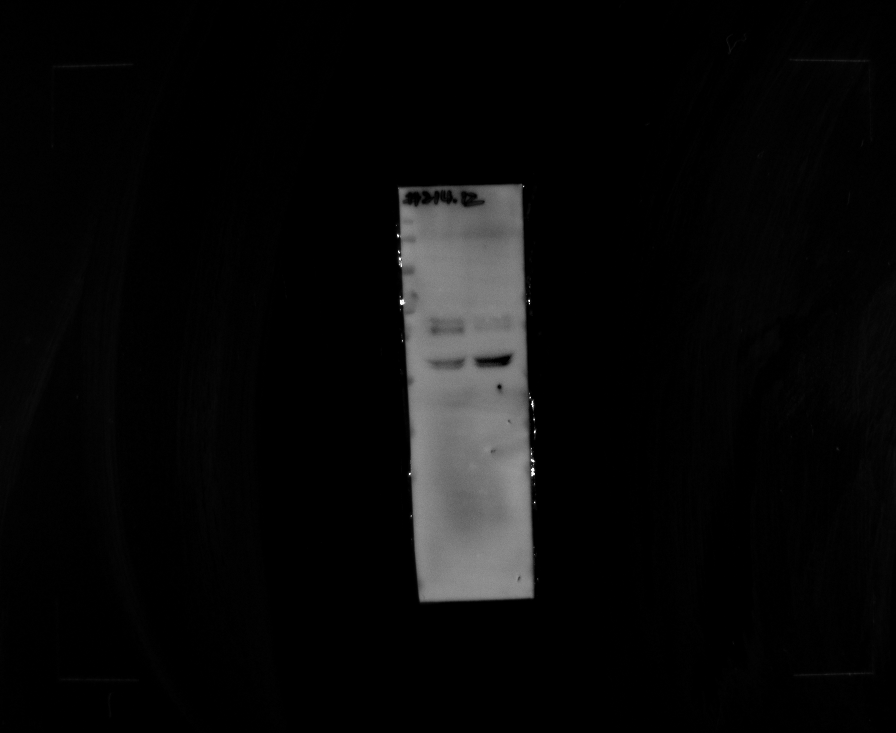
**

**TCF1**

**GAPDH**

**Figure 5D**


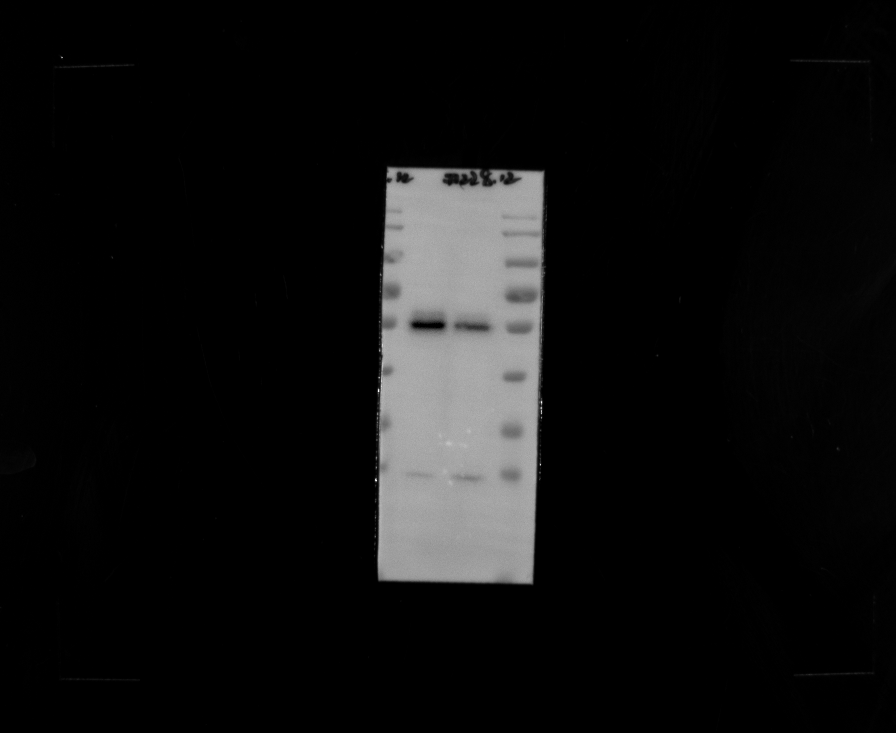

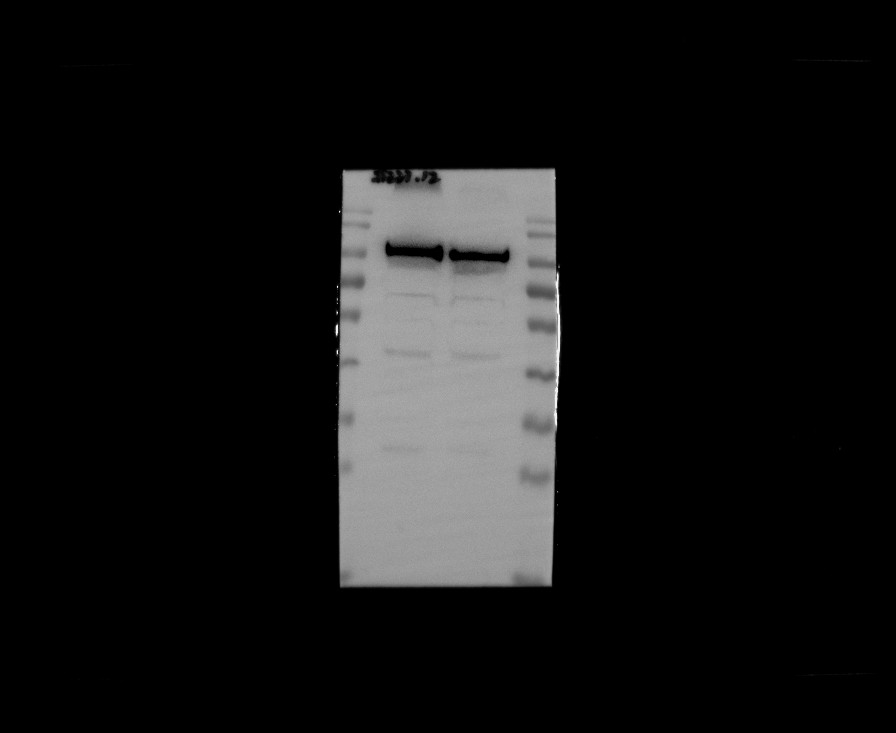

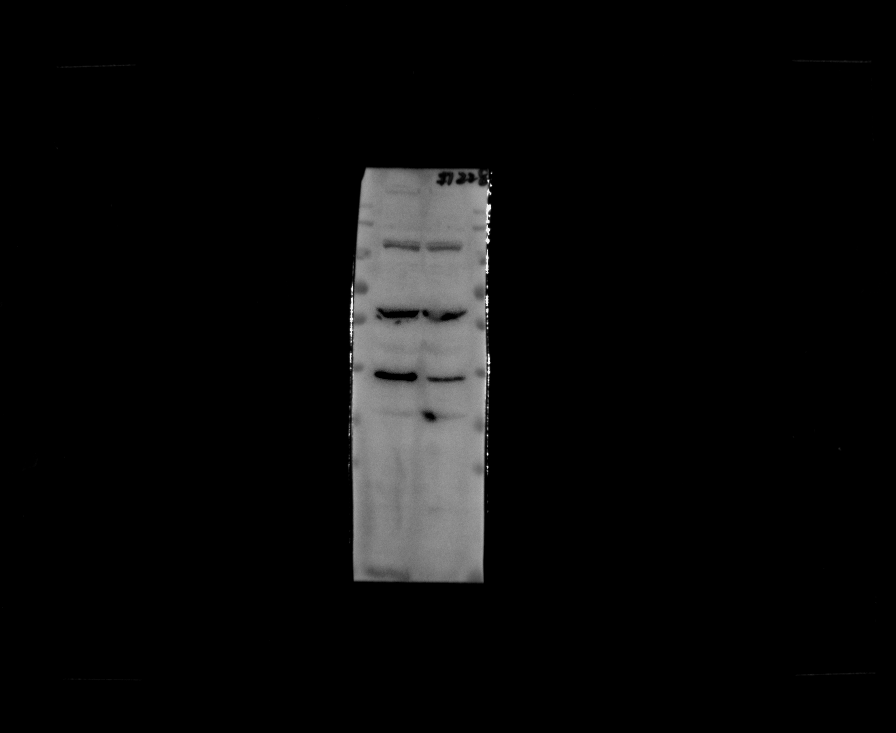


**WNT3A**

**β-catenin**

**c-Myc**


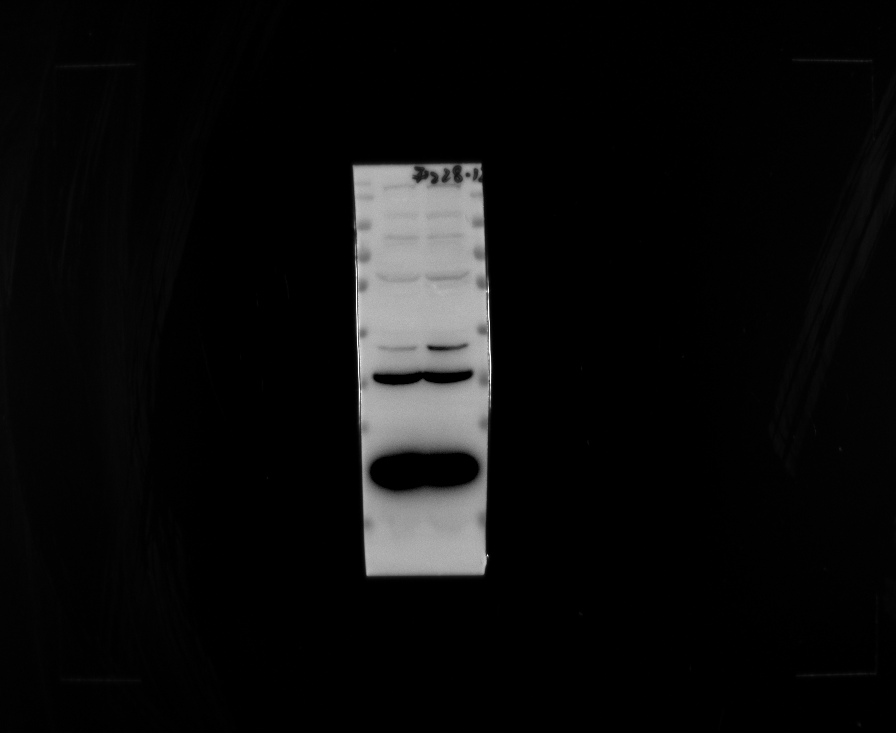

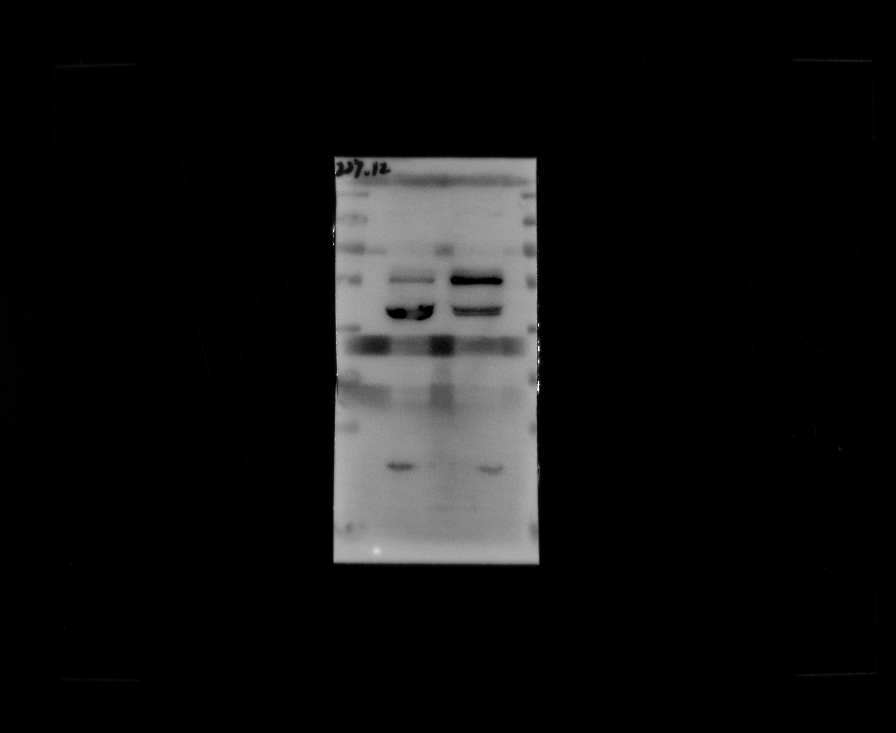


**GAPDH**

**TCF1**

**Figure 5F**

**
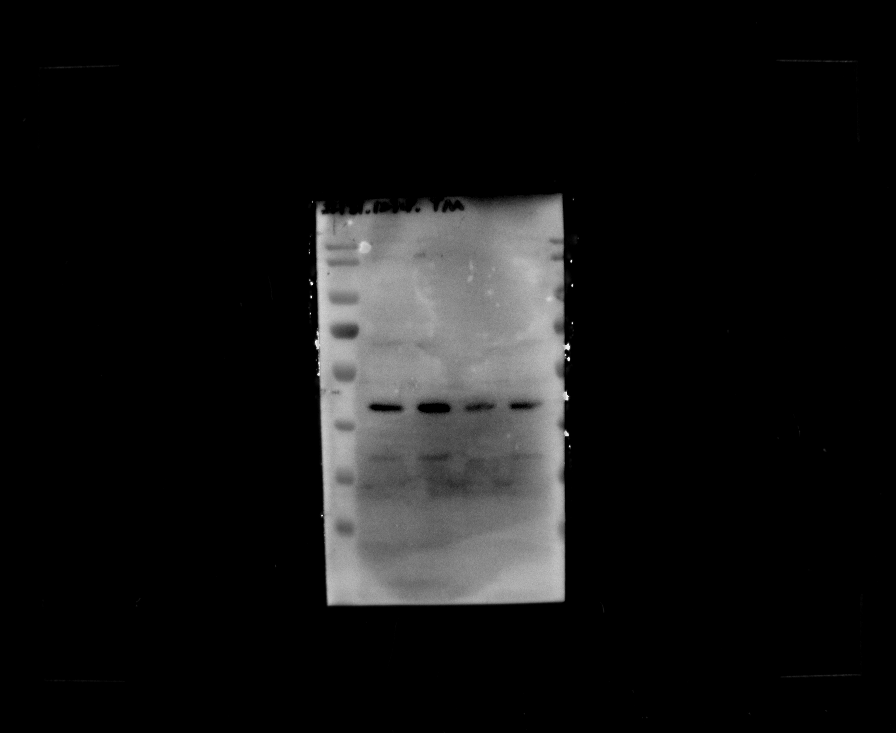

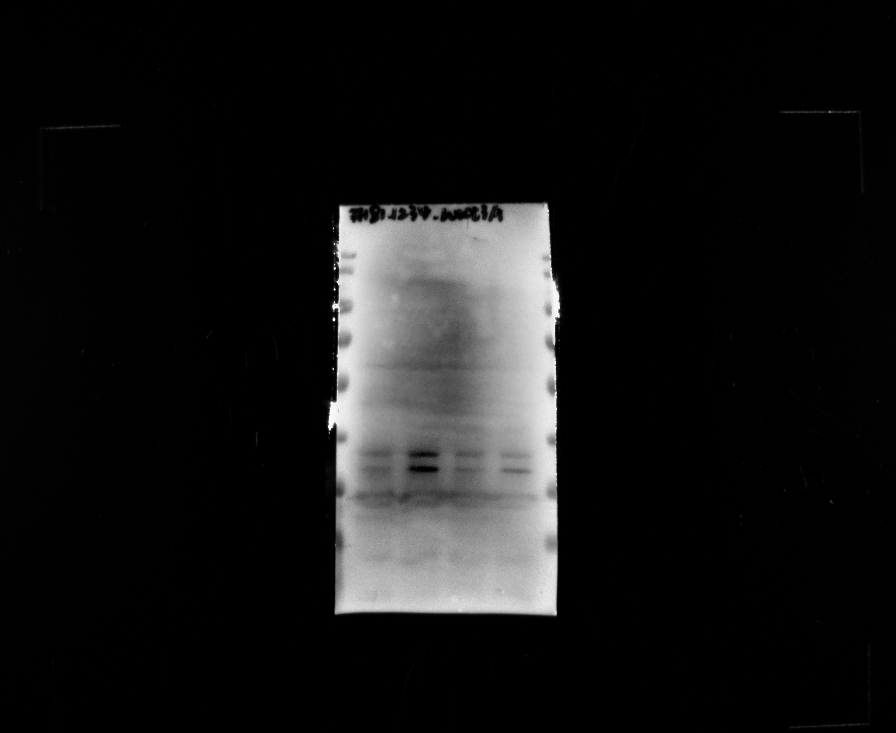
**

**WNT3A**

**TM7SF2**

**
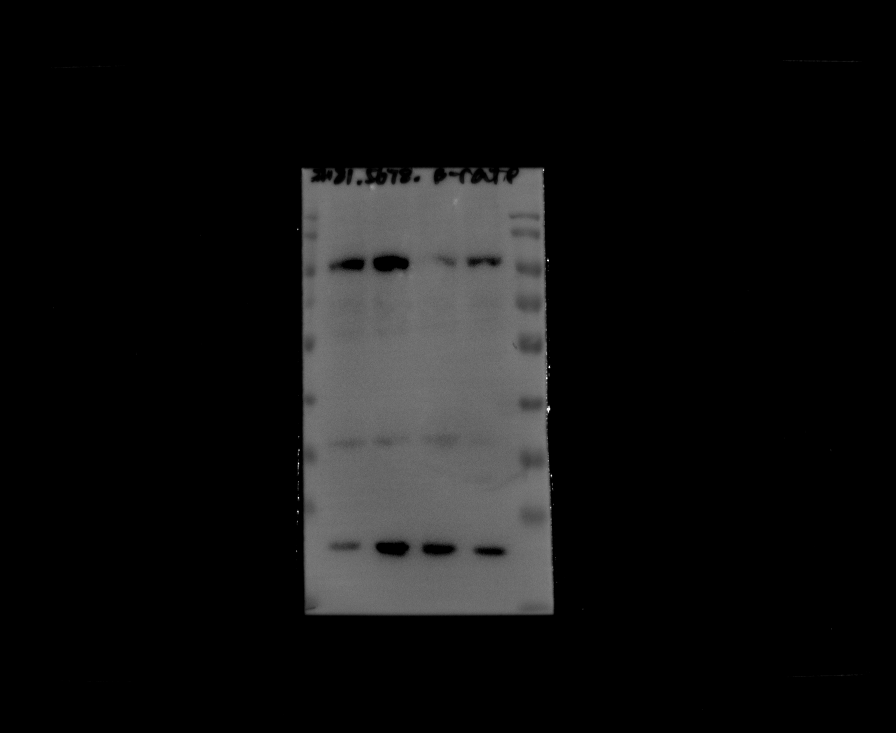

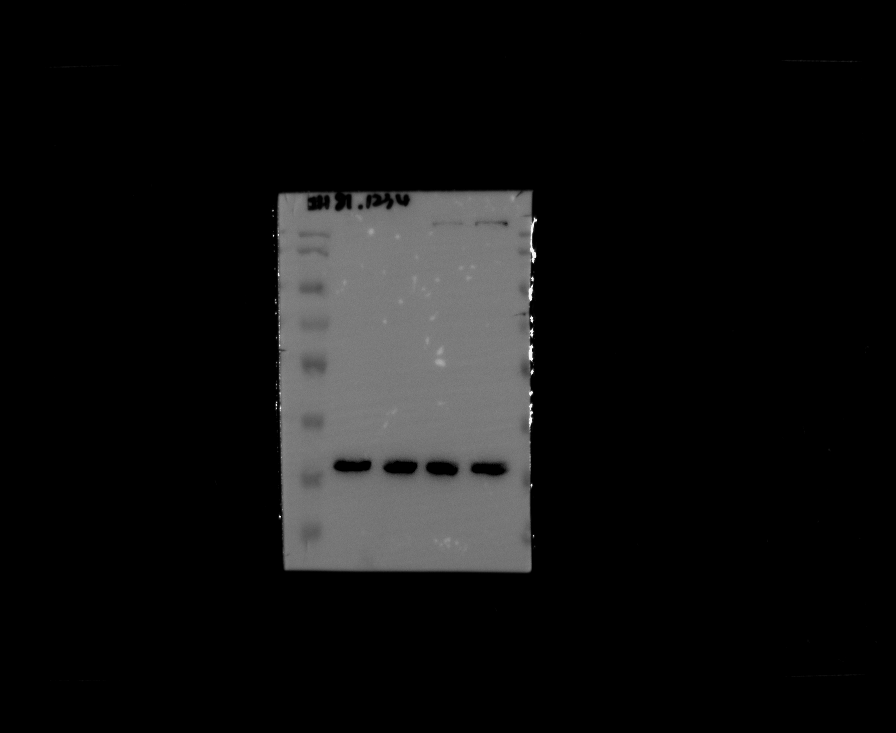
**

**GAPDH**

**β-catenin**

**Figure 5G**

**
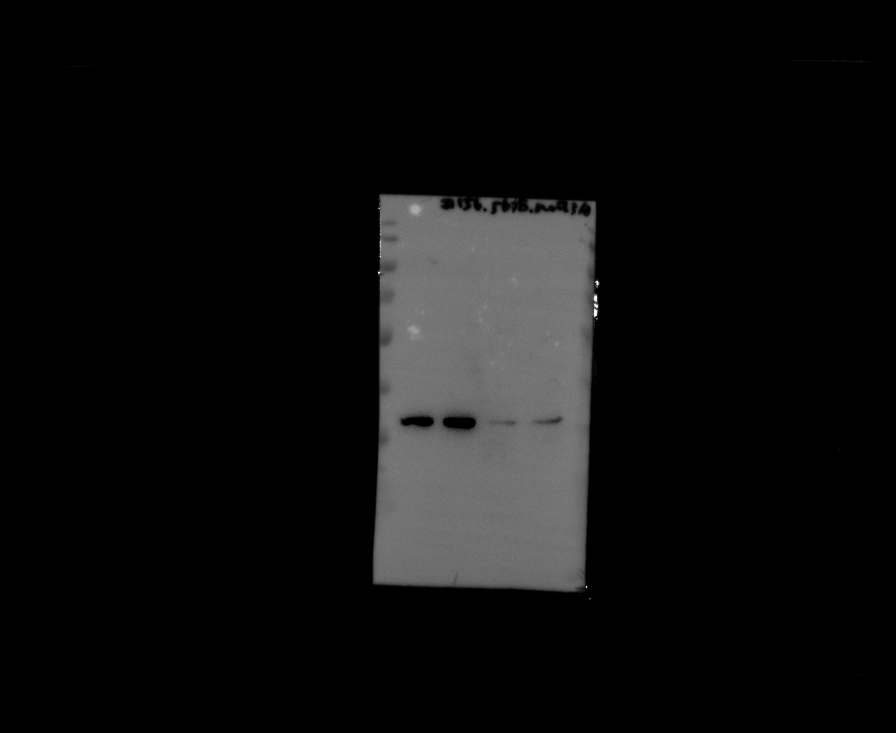

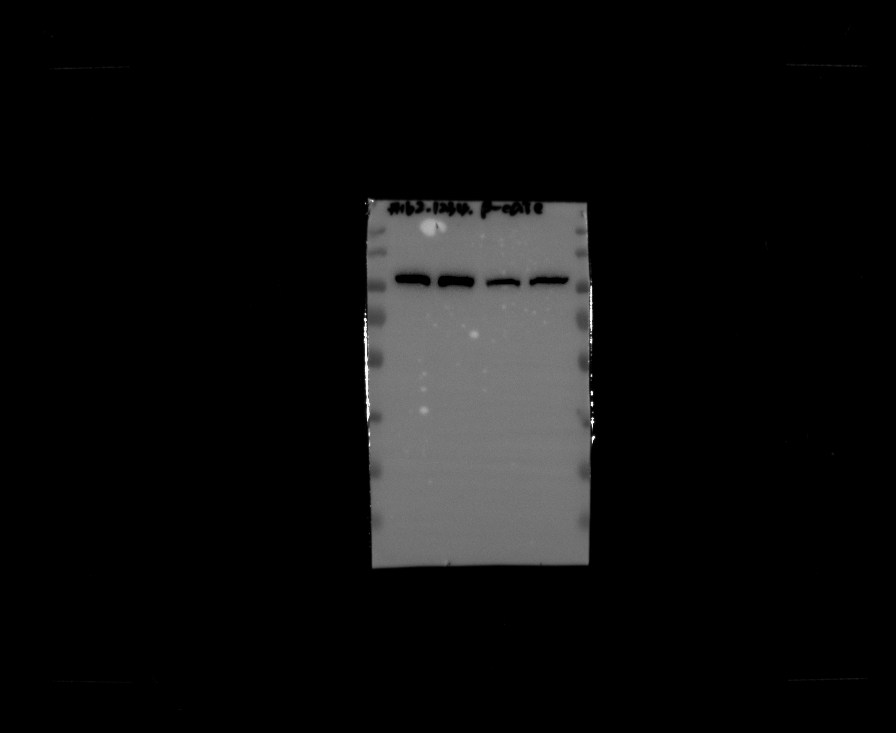
**

**β-catenin**

**WNT3A**

**
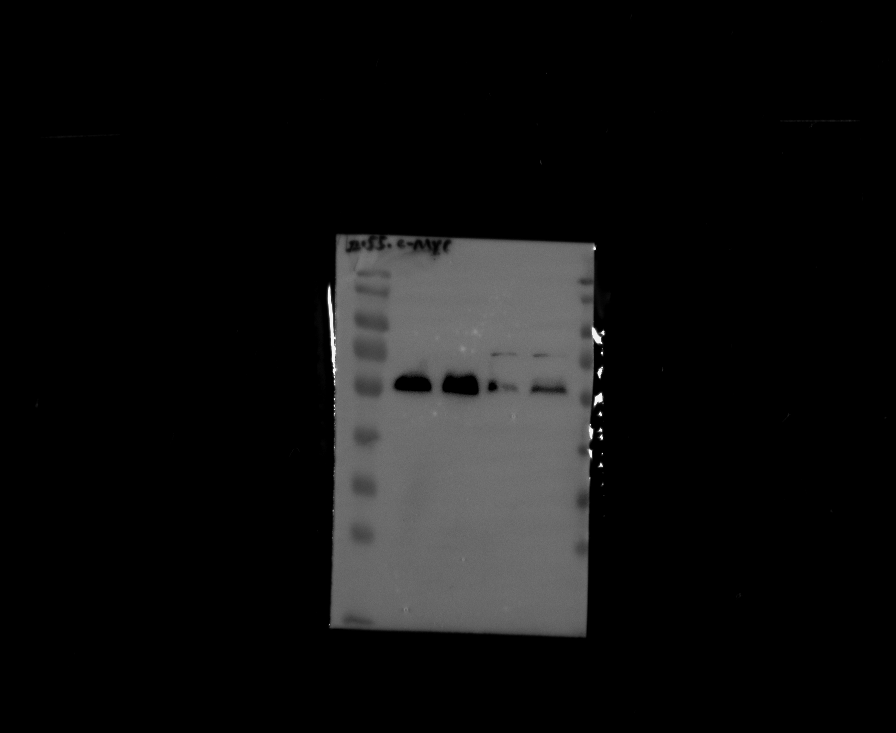

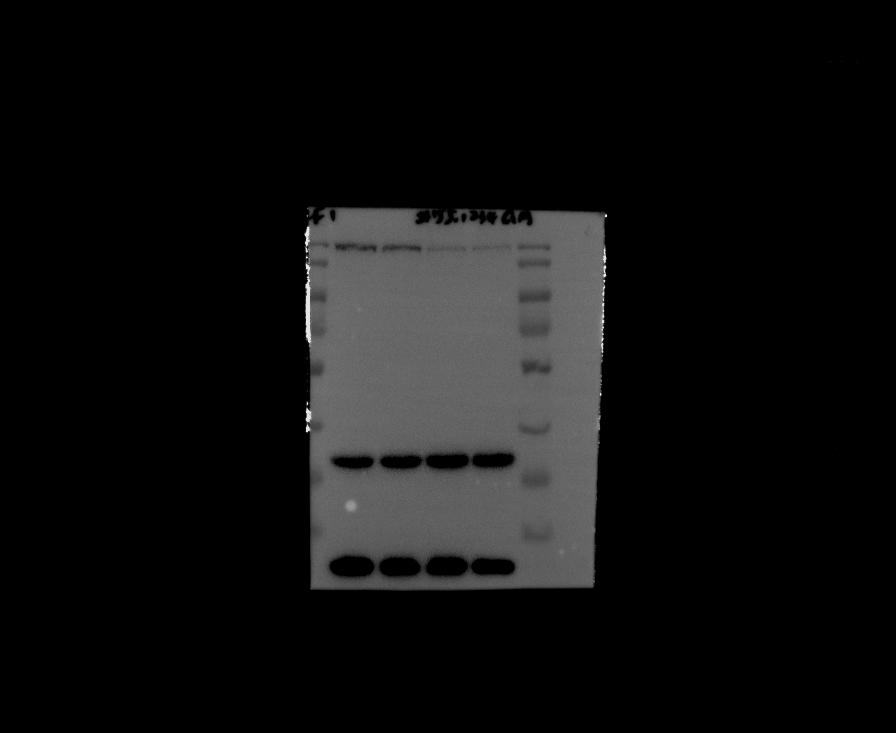
**

**GAPDH**

**c-Myc**

**Figure 5
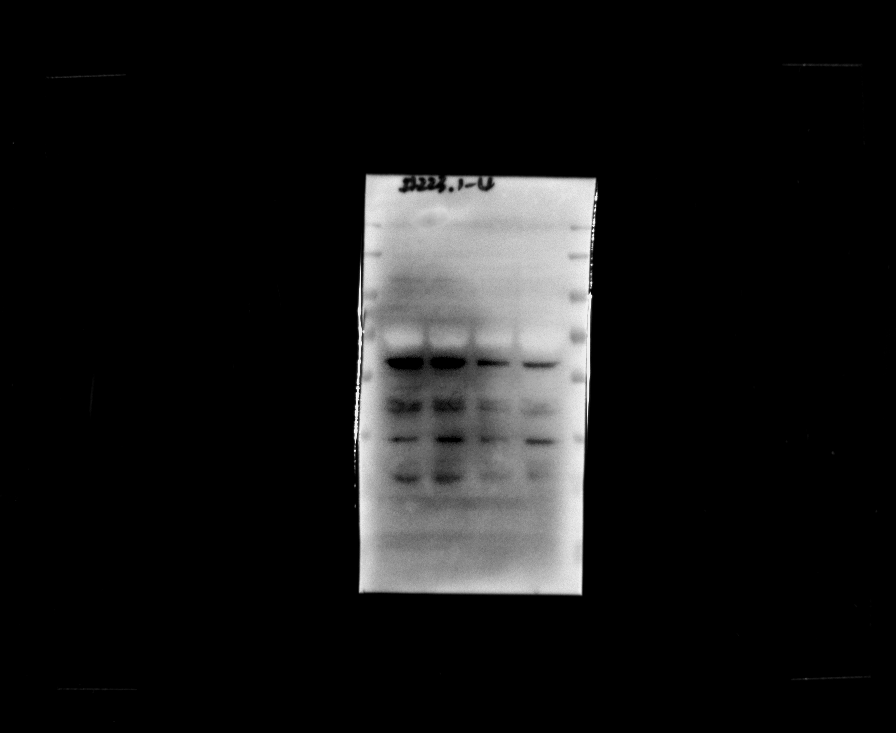

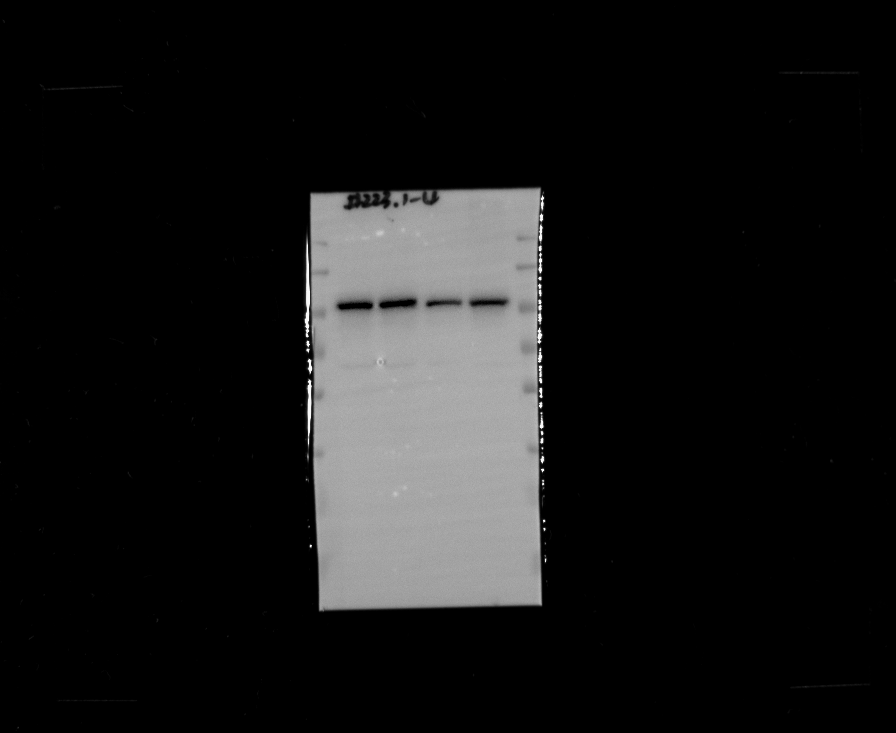

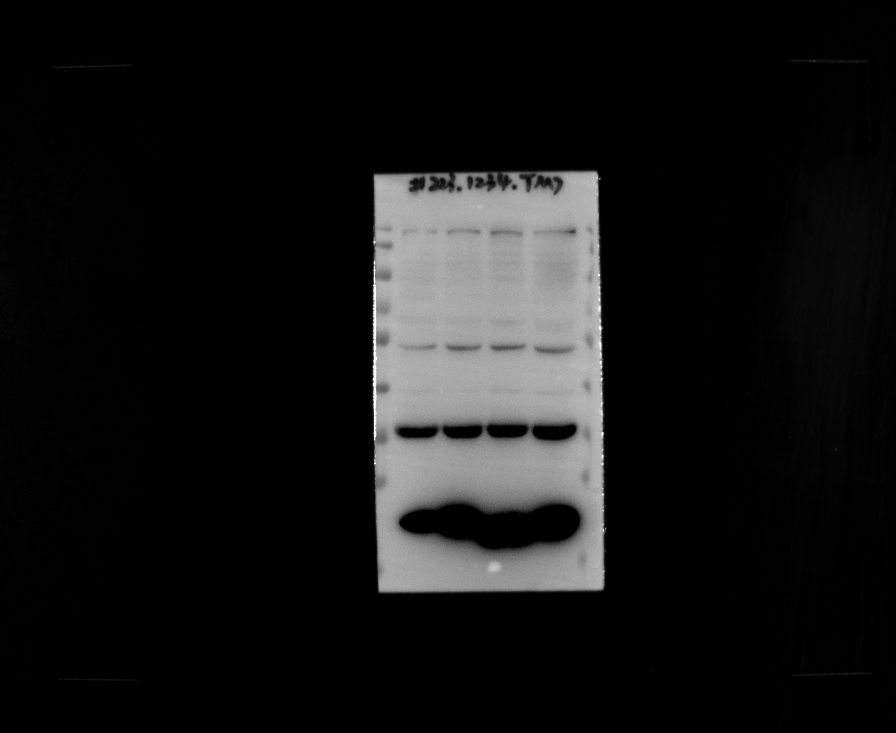

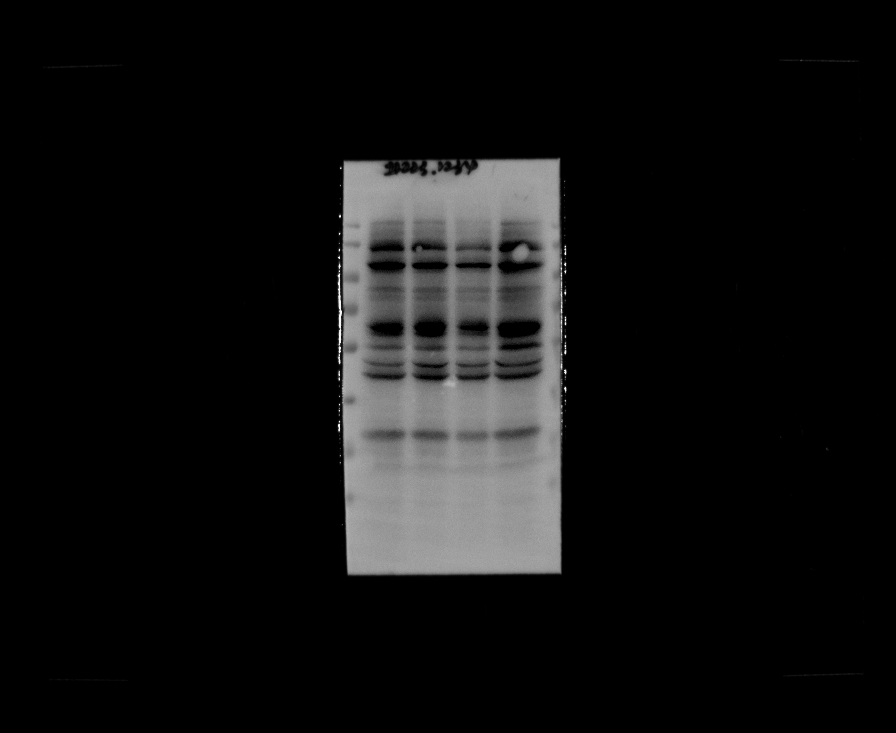
H**

**TM7SF2**

**GAPDH**

**β-catenin**

**WNT3A**

**Figure 5I**

**
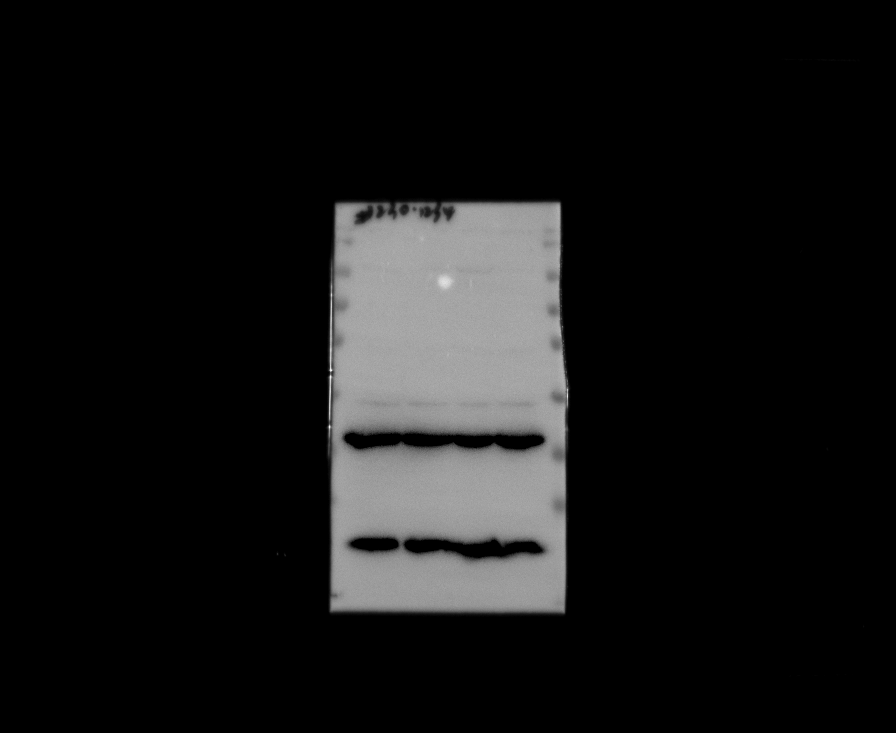

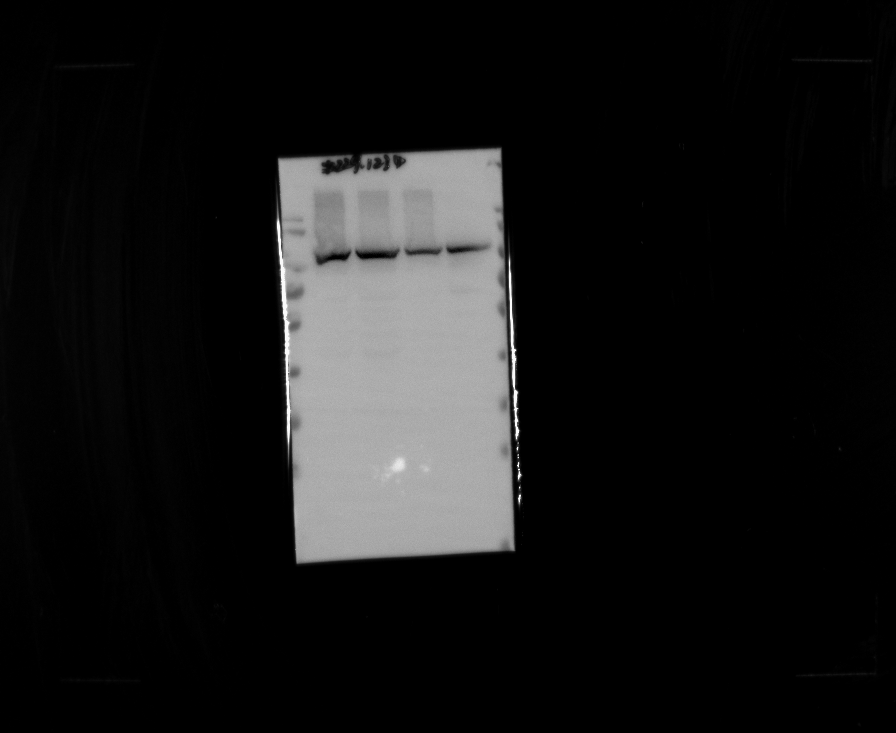

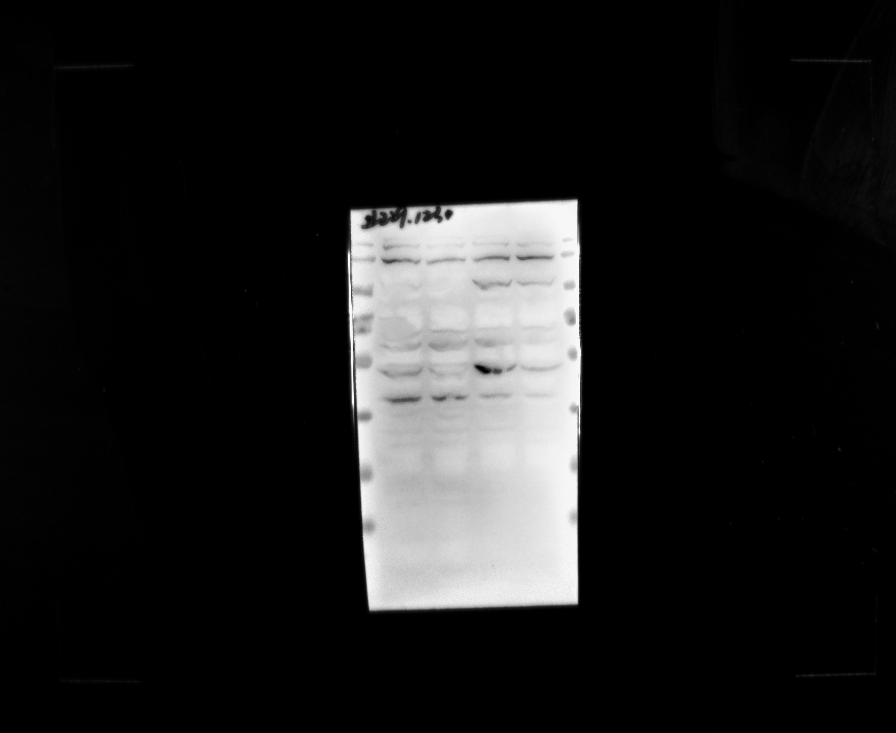

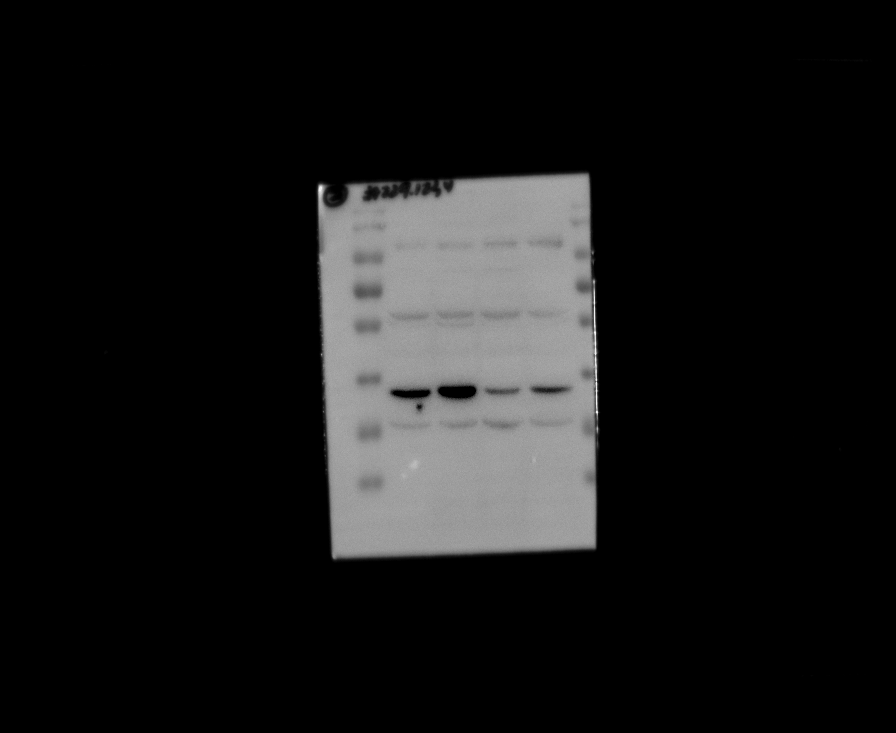
**

**β-catenin**

**GAPDH**

**TM7SF2**

**WNT3A**

**Figure 6A**

**
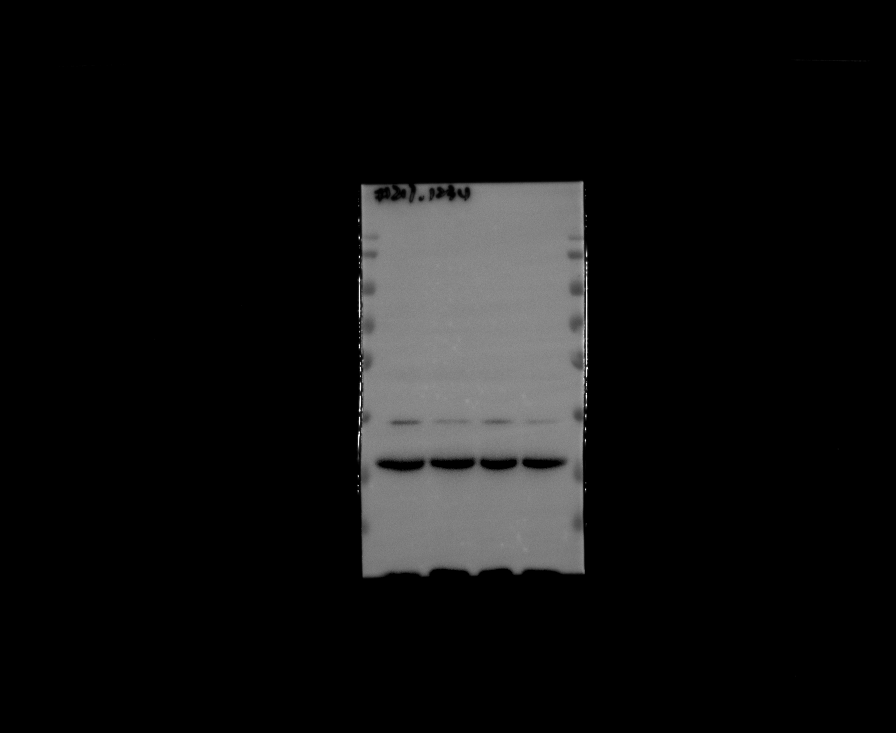

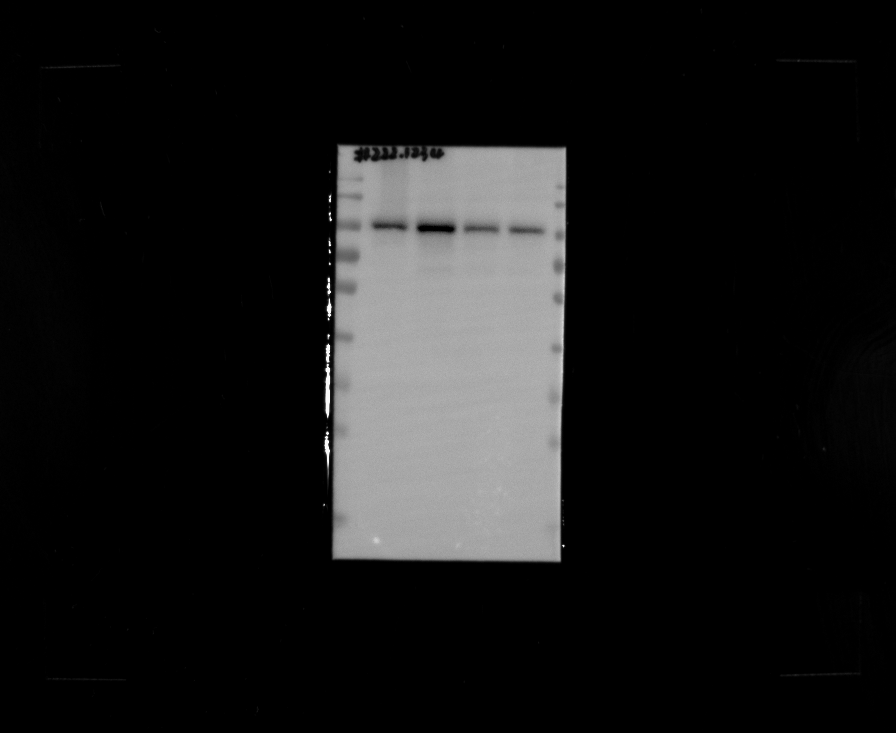

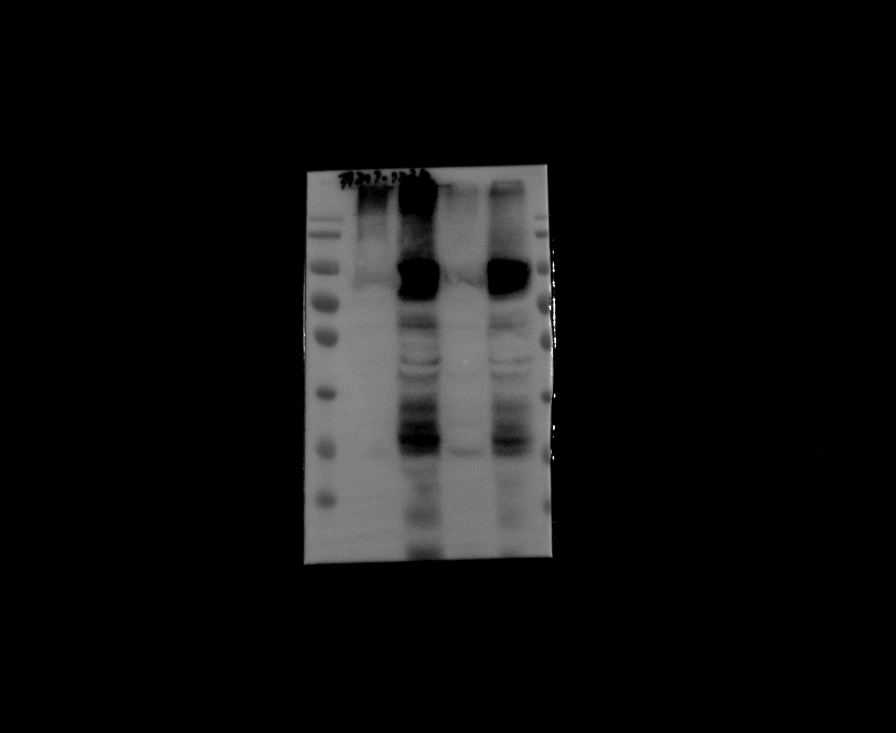
**

**β-catenin**

**CPT1A**

**GAPDH**

**Figure 6B**

**
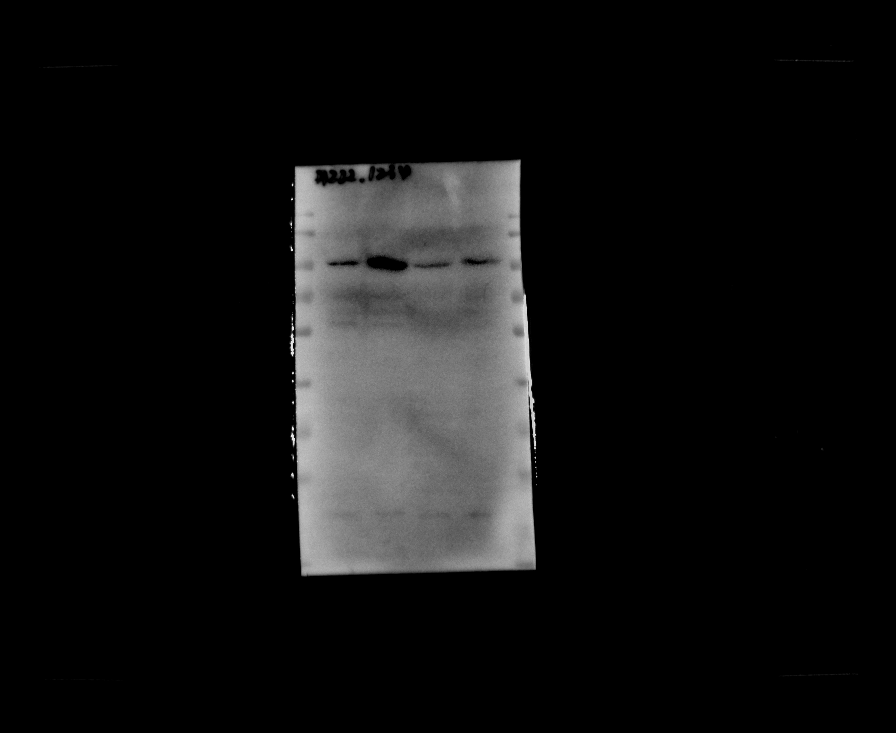

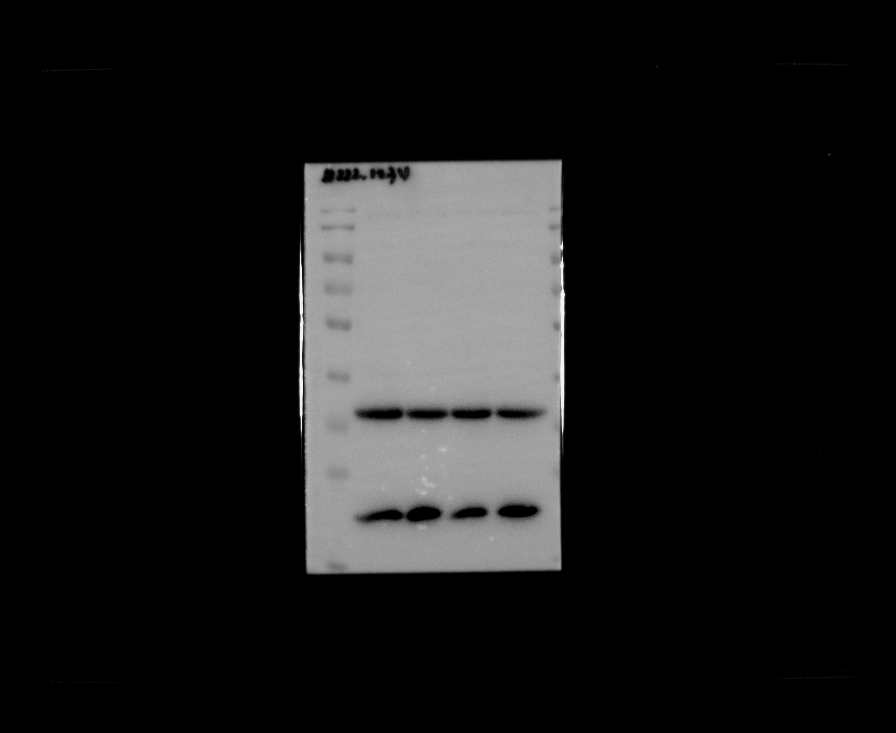

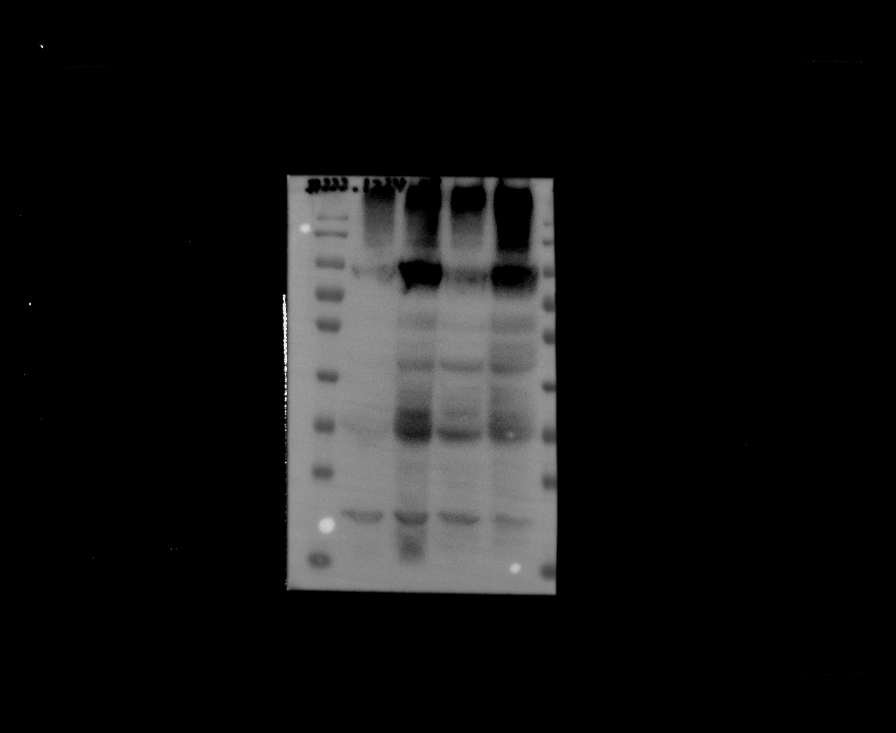
**

**CPT1A**

**β-catenin**

**GAPDH**

**Figure S1E**

**
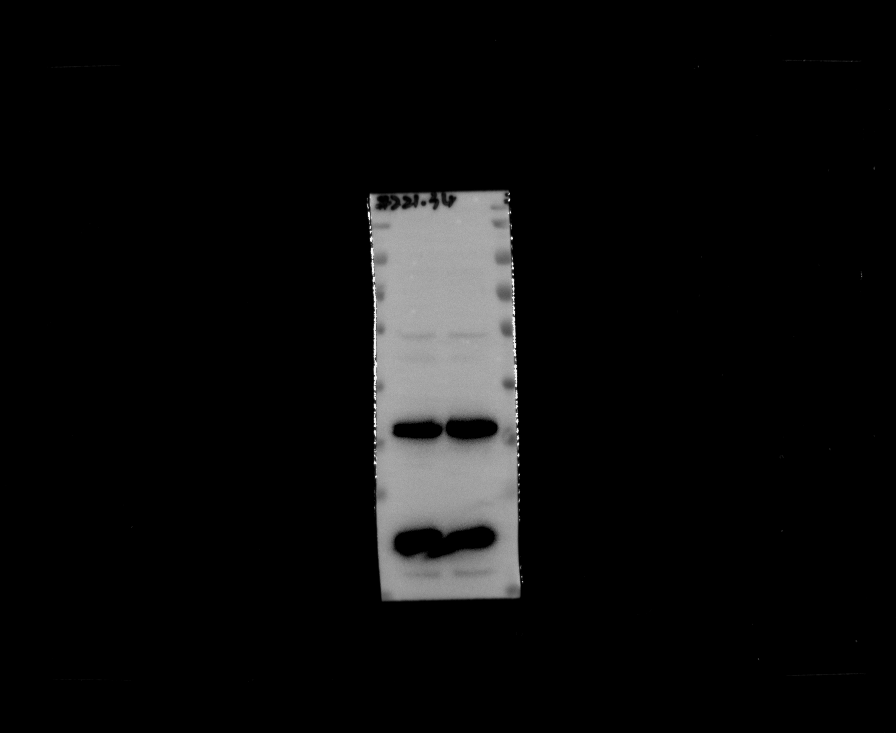

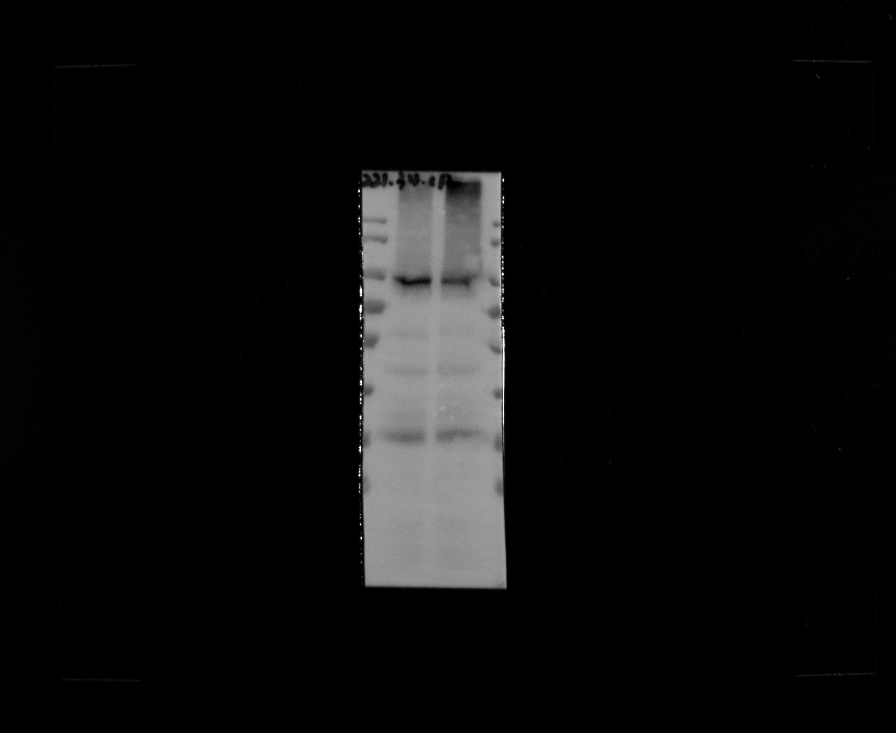
**

**CPT1A**

**GAPDH**

**
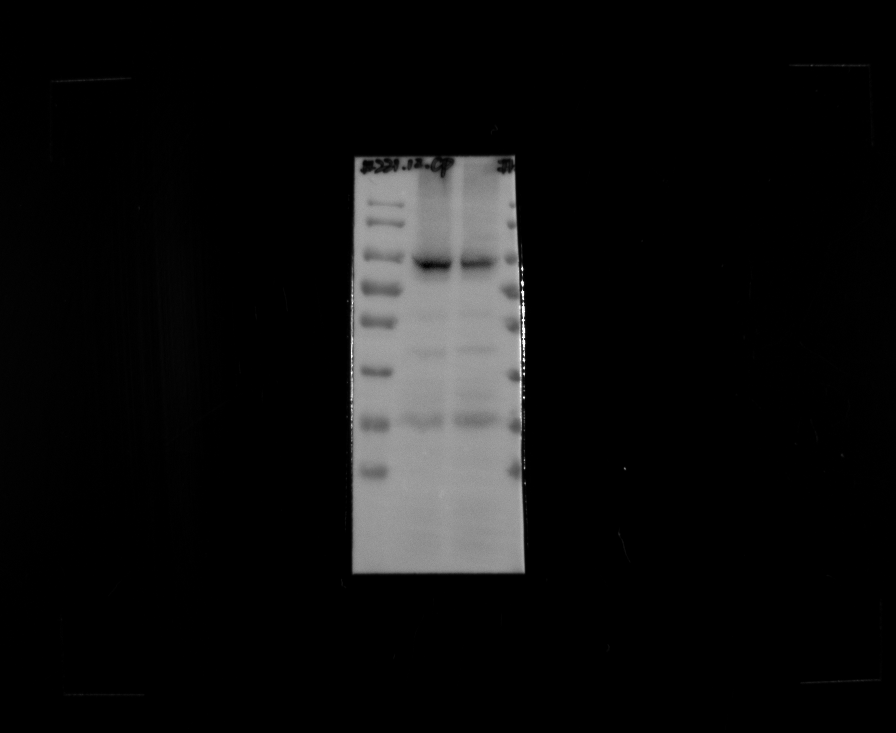
Figure S1F**

**
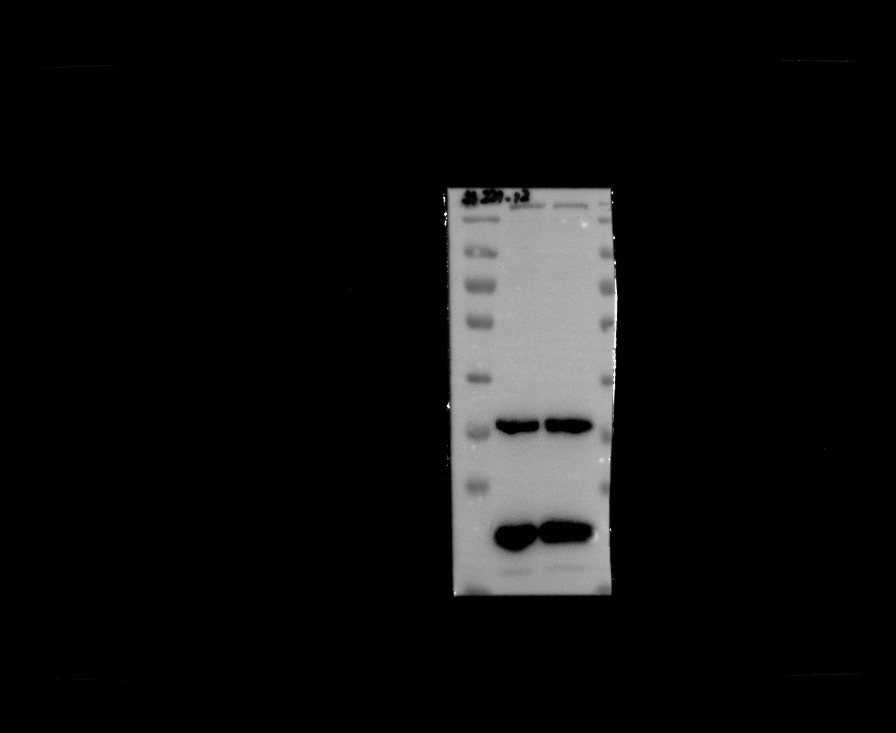
**

**CPT1A**

**GAPDH**

**Figure S1G**

**
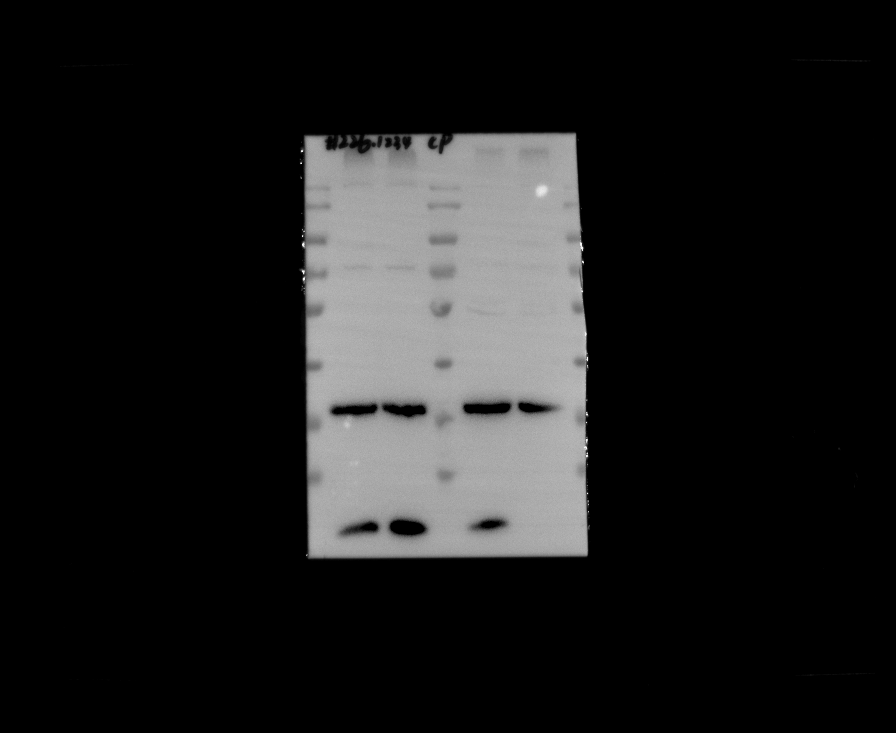

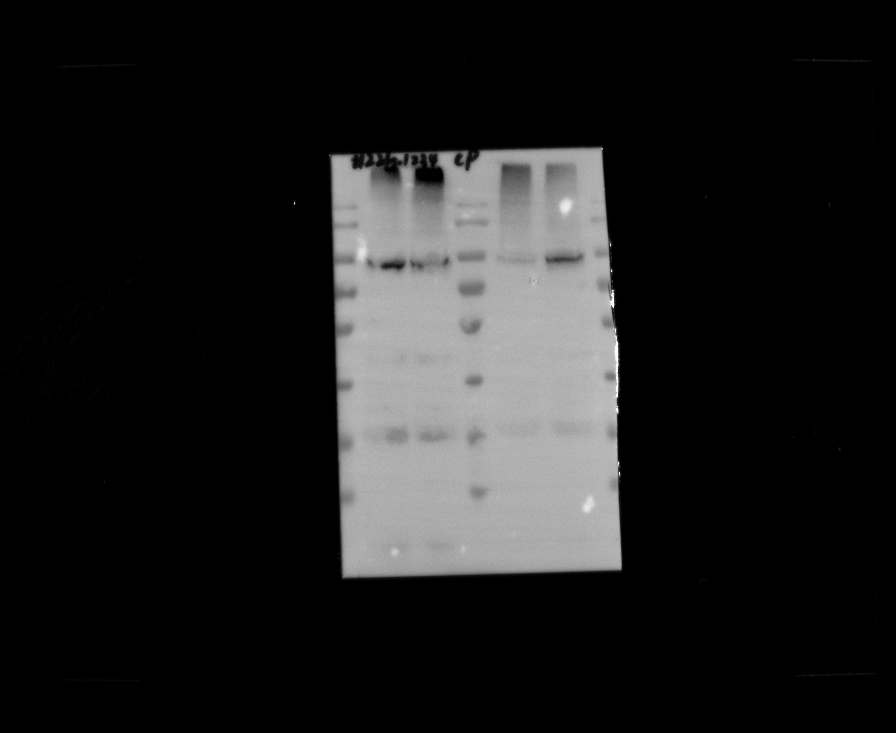
**

**CPT1A**

**GAPDH**

**Figure S1H**

**
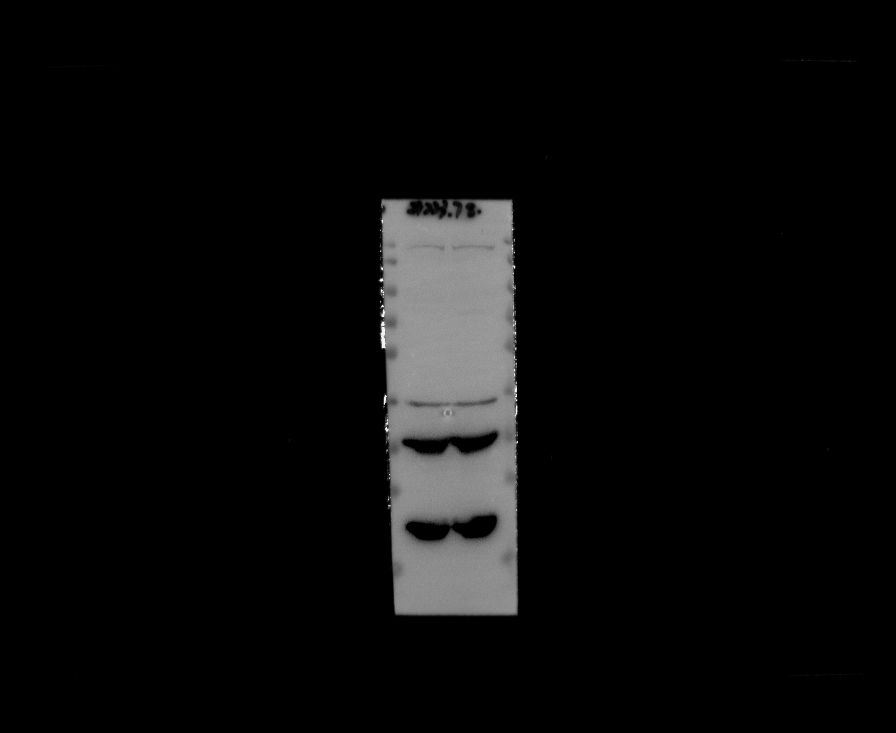

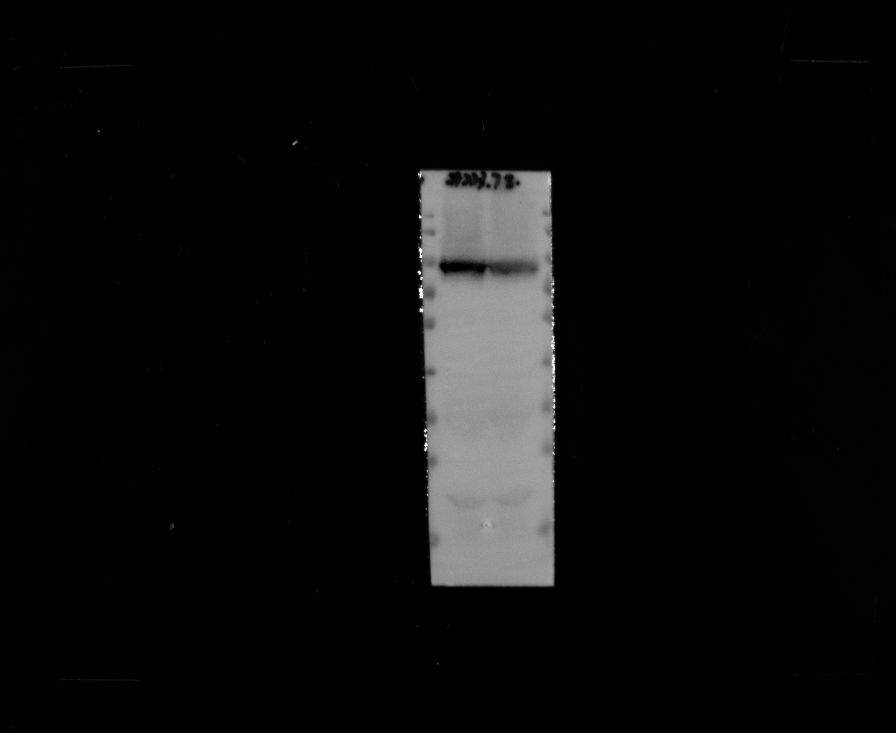
**

**CPT1A**

**GAPDH**
